# Effect of sugar-sweetened beverage taxation on sugars intake and dental caries: an umbrella review of a global perspective

**DOI:** 10.1186/s12889-023-15884-5

**Published:** 2023-05-27

**Authors:** Maryam Hajishafiee, Kostas Kapellas, Stefan Listl, Madhuri Pattamatta, Athanasios Gkekas, Paula Moynihan

**Affiliations:** 1 Adelaide Dental School, The University of Adelaide, Adelaide, Australia; 2 Department of Dentistry - Quality and Safety of Oral Health Care, Radboud University Medical Center, Radboud Institute for Health Sciences, Nijmegen, Netherlands; 3 York Trials Unit, Department of Health Sciences, University of York, York, UK

**Keywords:** Sugar-sweetened beverages, Taxation, Umbrella review, Oral health, Low-middle income countries, High-income countries

## Abstract

**Background:**

As part of the Global Strategy on Oral health, the World Health Organization (WHO) is exploring cost-effective interventions for oral health, including taxation on sugar-sweetened beverages (SSBs). To inform this process, this umbrella review aimed to identify the best available estimates pertaining to the impact of SSB taxation on the reduction of sugars intake, and the sugars-caries dose–response, such that estimates of the impact of SSB taxation on averting dental caries in both high (HIC) and low and middle (LMIC) countries be available.

**Methods:**

The questions addressed were: (1) what are the effects of SSB taxation on consumption of SSBs and (2) sugars? (3) What is the effect on caries of decreasing sugars? and (4) what is the likely impact of a 20% volumetric SSB tax on the number of active caries prevented over 10 years? Data sources included PubMed, Embase, Web of Science, Scopus, CINAHL, Dentistry and Oral Sciences Source, Cochrane Library, Joanna Briggs Institute (JBI) Systematic Review Register, and PROSPERO. The review was conducted with reference to JBI guidelines. The quality of included systematic reviews was assessed using AMSTAR to identify best evidence.

**Results:**

From 419 systematic reviews identified for questions 1 & 2, and 103 for question 3, 48 (Questions 1 & 2) and 21 (Question 3) underwent full text screening, yielding 14 and five included reviews respectively. Best available data indicated a 10% tax would reduce SSB intake by 10.0% (95% CI: -5.0, 14.7%) in HIC and by 9% (range -6.0 to 12.0%) in LMIC, and that a 20% tax would reduce free sugars intake on average by 4.0 g/d in LMIC and 4.4 g/d in HIC. Based on best available dose response data, this could reduce the number of teeth with caries per adults (HIC and LMIC) by 0.03 and caries occurrence in children by 2.7% (LMIC) and 2.9% (HIC), over a 10-year period.

**Conclusion:**

Best available data suggest a 20% volumetric SSB tax would have a modest impact on prevalence and severity of dental caries in both HIC and LMIC.

**Supplementary Information:**

The online version contains supplementary material available at 10.1186/s12889-023-15884-5.

## Introduction

In response to the World Health Assembly Resolution on Oral Health (WHA74.5) a Draft Global Strategy on Oral Health has been developed [1]. This includes the Strategic Objective *‘to enable all people to achieve the best possible oral health and target and reduce the social and commercial determinants and risk factors of oral diseases and conditions.’* The Global Strategy on Oral health, aims to recommend cost-effective oral health interventions by 2024. This will form part of the updated Appendix 3 of the WHO Global action plan on the prevention and control of non-communicable diseases (NCDs). WHO Member States will be guided in developing national responses to promote oral health and reduce oral health inequalities and diseases including dental caries; globally.

Dental caries is the most prevalent oral disease [2]. The role of dietary sugars in its aetiology is well established [3, 4]. A WHO recommendation, for minimising lifelong risk of dental caries, is to limit free sugars intake to below 5% of energy intake. This puts forth an important strategy for caries prevention [5]. Addressing the universally high free sugars intake, is an important part of the Global Strategy on Oral Health [1], yet public health measures to reduce sugars consumption are rare [6, 7]. To make a tangible difference, oral health policy and action plans need to move away from approaches that rely entirely on individual dietary behaviour change for free sugars reduction. Creating social and economic structures to support people to make behaviour changes will require taking bold action to focusing on upstream interventions to limit free sugars intake [8–10]. One such measure is the implementation of taxation on sugar-sweetened beverages (SSB).

SSB are a common source of dietary free sugars, with an average global consumption by children of 326 ml/day (ranging from 115 ml/day in Australia to 710 ml/day in China) [11]. In European countries SSB contribution to free sugars varies between 0–65% [12] and in the US SSB contribute to added sugars intake by 16–23% [13]. These data suggest that lowering SSB intake could significantly impact on free sugars intake. The WHO recognises, and provides guidance on, the implementation of taxes on SSB as an evidence-based policy to prevent obesity and non-communicable diet-related diseases [6]. SSB taxes have been introduced in several countries around the world to incentivise healthy beverage choice [14]. A wealth of evidence suggests that this has a positive impact on obesity prevention [15], though little attention has focused on the impact on the global impact on dental caries.

Within-country-based modelling studies aiming to predict the impact of taxation of SSB on subsequent levels of dental caries are largely from high income countries (HIC) [16–19]. However, a diversity of approaches and assumptions has led to vast between-study differences in effect sizes and irreproducible results. The impacts of SSB taxation on development of dental caries in low- and middle-income countries (LMIC) have not been broadly reported [20]. The challenges in publishing in mainstream journals of HIC faced by authors from LMIC, may explain this observation. With a view to assisting WHO in the identification of cost-effective interventions, an independent assessment of the evidence pertaining to the predicted impact of SSB taxation on dental caries in both HIC and LMIC on prevention of dental caries was conducted. For this purpose, an Umbrella Review approach was chosen. An umbrella review is a review of systematic reviews [21] that captures the vast amount of evidence contained in systematic reviews and studies to access research evidence and inform decision-making. They provide a summary of existing research syntheses related to a given topic or question and are also applied when there is a need for “fast” evidence in reduced timeframes. Umbrella reviews can be used to summarize more than one research synthesis e.g., for different populations or geographic locations. Using an umbrella review approach, the aims of this study were first, to identify the available data pertaining to the impact of SSB taxation on consumption of SSB (i.e., the price elasticity of demand (PED) and sugars, and of the impact of the reduced sugars intake on the development of dental caries; and second, to utilise these data as model parameters to assess the impact of SSB taxation on caries prevention in both LMIC and HIC. The objectives were first, to conduct an umbrella review to identify the best available evidence pertaining to the PED of SSB and the impact of taxation on sugars consumption, and of sugars consumption on the development of dental caries; and second, to use these data to estimate the potential impact of introducing a 20% SSB volumetric tax on averting dental caries.

## Methods

A preliminary search for previous umbrella reviews on the topic was conducted in PubMed and Web of Science and no existing reviews were identified. The protocol for this umbrella review was registered on PROSPERO in January 2022 (CRD 42022293187) [22]. The review was guided by the Methodology for Joanna Briggs Institute (JBI) Umbrella Reviews [21]. The following questions were addressed:

1. What are the effects of SSB taxation on SSB on PED/ consumption?
2. What is the effect of SSB taxation on consumption of sugars?
3. What are the effects of decreasing sugars consumption on levels of dental caries?
4. What is the likely effect of a 20% volumetric tax on averting dental caries over a 10-year period?

### Population

Studies of healthy populations (i.e., reviews that do not specifically target participants with disease), all age groups, race, gender and geographic locations were included. Data pertaining to the above questions was explored by age group (adults, children (for dental caries outcomes children were further classified according to whether outcomes related to the primary dentition, the permanent dentition or both), geographic location (country), income classification of included countries (high, middle, and low), socioeconomic status of participants and type of tax. To facilitate best-possible context-specificity, where possible, the evidence was mapped according to the WHO Regions.

### Intervention/exposure and outcomes

For Questions 1 and 2, systematic reviews of studies that measured the impact of any type of tax to SSB including *ad valorem*, volumetric tax, or nutrient based tax (based on the sugars content of the drink) were included. Different levels of taxes, including excise tax and sales tax, applied by government, manufacturer, or retailer, were considered.

For Question 1, the outcome was measure of SSB consumption measured as sales, household consumption level, and data on consumption from surveys of dietary intake. SSB included beverages with added sugars, e.g., carbonated and still beverages and sweetened fruit juices, excluding beverages sweetened exclusively with artificially sweeteners. Reports on change in consumption expressed as amount (e.g., mL/day, week, or year, or change in energy intake (EI) from SSB) or units of frequency from which amount can be derived, were included. Data on change in consumption of SSB derived from data on PED including own price elasticity (OPE) and cross price elasticity (CPE) of SSB were included.

For Question 2, the outcome measure was a quantitative change in the intake of sugars (free sugars) expressed as grams, kilograms or ounces per day, week, or year, or the amount of sugars expressed as a percent of EI. Outcomes also included data on the change in energy intake when expressed as Kcal or kJ, MJ per day, month or year, which allowed for conversion to a quantified amount of sugars using the Atwater Factor of 4 kcal/gram sugars [23].

For Question 3, systematic reviews were included if they reported data pertaining to a quantified measure of sugars intake or change in sugars intake. Sugars intake included total sugars (and any component of) i.e., free sugars, added sugars, sucrose, non-milk extrinsic sugars, expressed as g or kg/day or /year or as a percentage of EI, or a per capita population intake or availability. Systematic reviews that reported solely on the frequency of sugars consumption were excluded.

For Question 3, the outcome was a measure of dental caries. This included decayed, missing and filled teeth (DMFT (for permanent teeth), dmft (for primary teeth)), decayed, missing and filled surfaces (DMFS (for permanent teeth), dmfs (for primary teeth)), decayed, extracted due to caries, filled teeth (deft), or comparisons between caries and no caries or higher caries vs. lower caries.

### Sources of information

Online biomedical databases, including PubMed, Embase, Web of Science, Scopus, CINAHL, and Dentistry and Oral Sciences Source were searched. Registries for systematic reviews, including Cochrane Library, the JBI Systematic Review Register and the PROSPERO Library, were searched. Databases were selected from available sources through The University of Adelaide library, aiming for broad geographical coverage. There were no restrictions placed on language and study duration. The date limit was from 2000 to end 2021 (to broadly cover the period since the introduction of taxes on SSBs [24]. The search strategy is presented in Additional file 1. In addition to the search, known experts were contacted by email to identify any further systematic reviews. The inclusion/exclusion criteria and outcome measures for each review question are described below.

### Types of study included

The umbrella review included exclusively systematic reviews with or without evidence synthesis, e.g., meta-analysis. For Questions 1 and 2, systematic reviews which examined the effects of SSB taxes on SSB consumption, included both naturalistic (explore the impact of real-world taxes) and modelling studies (hypothetical tax) that used cross sectional or longitudinal (before after) or time series data on price and consumption, or data from experimental intervention studies. Systematic reviews of studies with exclusively qualitative data were excluded.

For Question 3, systematic reviews which exclusively examined the effects of amount of sugars intake or changing sugars intake on dental caries levels of change in dental caries development were included. Reviews that incorporate text and opinion as their primary source of evidence were excluded.

### Study selection

Articles identified by searches were initially screened independently in duplicate by two authors to exclude systematic reviews clearly outside of scope. Potentially eligible reviews underwent full text review by independent duplicate assessment for inclusion. Differences between reviewers’ results were resolved by discussion. Reviews that were excluded at full-text screening are presented in Additional file 2. One reviewer extracted relevant data using a modified version of the JBI data extraction form [25] and a second reviewer checked data. Completed data extraction forms for included reviews are presented in Additional file 3 (Questions 1 and 2) and Additional file 4 (Question 3). Where pooled analysis was not available to answer a review question, data from original studies within systematic reviews was extracted and summarised.

### Quality of included data

The quality of eligible systematic reviews and evidence syntheses was assessed in duplicate using AMSTAR (A Measurement Tool to Assess Systematic Reviews) which is based on an 11-point scale. An AMSTAR score of 8–11 is categorised as high quality, 4–7 is moderate quality and 0–3 low quality [26]. Any disagreement between reviewers was resolved by involvement of a third reviewer. Assessments were conducted independently in duplicate by two reviewers without conflicting interest (e.g., not an author of the review) and any discrepancy between reviewers were resolved by discussion.

### Data summary

The approach to the presentation of findings from included systematic reviews included a tabulated summary of identified reviews that was presented in reverse chronological order and included information on AMSTAR rating. This was supported by a narrative summary that used a ‘best available evidence’ approach where data from the most recent high quality systematic review was described first, followed by a comparison with data from moderate quality reviews or earlier published reviews. Evidence synthesis from the most recent best quality systematic review was used to inform subsequent outputs. To enable a more detailed assessment and interpretation of the evidence when meta-analysis from SRs were not available, further data extraction of the characteristics and findings of primary studies included in the SRs was carried out. In this instance, data extracted included: author year, country of data collection, sample size, age, objectives, intervention or exposure, outcome, and quality assessment.

#### Estimated impact on levels of dental caries

The impact of SSB taxation on intake of free sugars was estimated in two ways. First, from the identified estimates of the impact of SSB taxation on the consumption of SSB (PED data from Question 1) together with available information on the level of SSB consumption in HIC and LMIC [11]. Second, using the identified estimated of the impact of SSB taxation on intake of energy and sugars (Question 2). In the absence of data from meta-analysis pertaining to sugars intake and dental caries, estimates of sugars reduction and the best available data were used. This included identified data on the dose response between sugars and development of dental caries, (identified in Question 3) to estimate the impact of a 20% volumetric SSB tax on the number of caries prevented in both children and adults over a period of 10 years (Question 4). The identified data for HIC and for LMIC were applied separately.

## Results

**Fig. 1:**
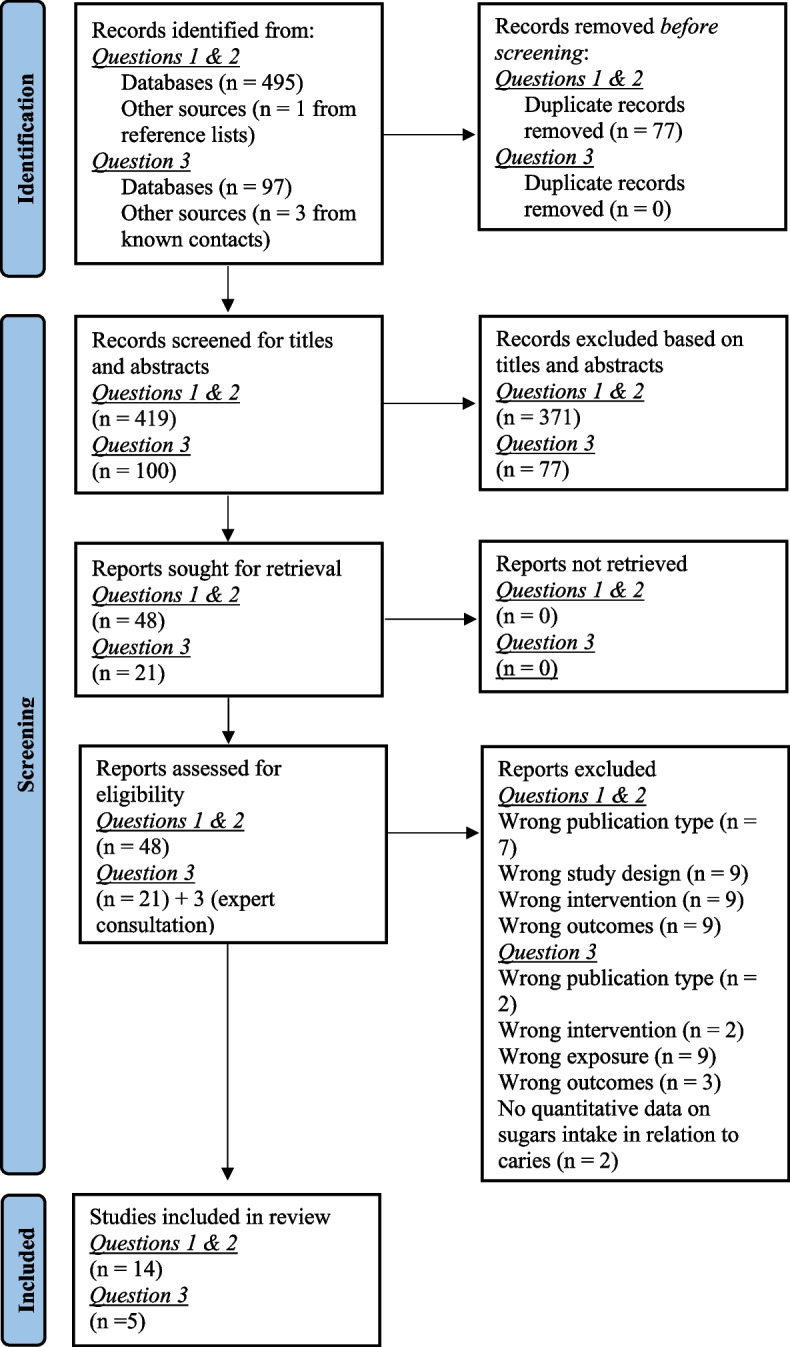
PRISMA flowchart of study selection and inclusion process

Figure 1 presents the PRISMA flow diagram. For Questions 1 & 2, from all databases combined, 419 systematic reviews were identified following de-duplication. Following title and abstract screening, 48 systematic reviews were retained for full text screening. Following full text screening, 14 systematic reviews were included. Four were rated as high quality [15, 27–29] nine as moderate quality [30–38] and one as low quality [39]. Two reviews did not make a declaration of conflict of interest [38, 39]. For question 3, from all databases combined, 100 systematic reviews were identified following de-duplication. Following title and abstract screening 21 reviews were retained for full text screening from which two systematic reviews were included; a further three systematic reviews were identified through known experts in the field giving five systematic reviews. Two reviews were rated as high quality [3, 4] and three as moderate quality [12, 40, 41]. One review did not make a declaration of conflicts of interests [40]. Reasons for exclusions of full texts are provided in Fig. 1 and Additional file 2.

### Question 1: Impact of taxation on SSB consumption and PED

Of the 14 systematic reviews identified that had data relevant to Question 1, eight included a narrative account only and six conducted evidence synthesis by pooling data or meta-analysis. Systematic reviews included data from the African, European, Southeast Asian, and Western Pacific WHO Regions as well as The Region of The Americas. A summary of included reviews along with the AMSTAR rating is provided in Table 1. Details of the AMSTAR rating for each included review is provided in Additional file 5. A summary of the original studies included in the 14 reviews, including the quality appraisal for these studies, is provided in Additional file 6.

**Table 1:** Summary of included systematic reviews with data pertaining to Question 1^a^

**Review objectives**	**Review study inclusion criteria**	**Countries (WHO regions) of included relevant studies**	**Method of evidence synthesis**	**Consumption related outcomes relevant to this umbrella review**	**Included relevant original papers and quality**
**Itria et al. **[15]**. AMSTAR Rating: High**					
To assess the effect of implemented SSB tax policies on reducing consumption, purchase and sales, overweight and obesity prevalence	Any type of tax Studies included modelling, non-experimental quasi-experimental or experimental Peer-reviewed and grey literature	Australia; Barbados; Chile; Ireland; Mexico; South Africa; UK; USA (African, European, Western Pacific, The Americas)	Narrative	Outcomes included changes in SSB consumption including purchase/sales as proxy for consumption. (See data from individual studies in Additional file 6)	16 papers: [42–57] Out of 7 elements of quality assessment, all included studies achieved scores of 4–5
**Sobhani et al. **[30]** AMSTAR Rating: Moderate**					
To assess evidence on the impacts of SSB taxation on purchase and consumption of SSBs	Excise tax/sales tax Empirical data excluded modelling studies. Included quasi-experimental (pre- post comparisons) and RCT designs	Mexico USA Holland (European, The Americas)	Narrative	Outcomes included consumption, purchase, or sales of SSB (See data from individual studies Additional file 6)	5 studies with data relevant to the question of this review: [44, 58–61] Out of 7 elements of quality assessment, all included studies achieved scores of 4–5
**Teng et al. **[27]** AMSTAR Rating: High**					
To meta-analyse data from studies measuring impact of real-world SSB taxes on SSB sales and intake before and after the tax, or in a taxed compared with an untaxed jurisdiction	Studies with data on the impact of implementation of real world SSB taxes All types of SSB taxes (excise, sales, volumetric, *ad valorem*, sugars content-based taxes	All were HIC except Mexico (high-middle) Chile Finland France Hungary Mexico Spain USA (European, The Americas)	Meta-analysis: the summary measure was a RR scaled for 10% *ad-valorem* tax (AVEs were applied to volumetric tax). Sub-analysis by tax type	Outcomes included purchase or dietary intake Meta‐analysis showed 10% SSB tax decreased consumption by 10.0% (95% CI: − 5.0, -14.7%), based on pre–post intervention comparisons and/or comparisons to an untaxed control jurisdiction. This corresponded to PED − 1.00 (95% CI: − 0.50, − 1.47) By tax type: 2.3% (95% CI: -11.2, 7.4%) decline for *ad valorem* tax; a 10.2% decline (95% CI: -4.1, -15.9%) for volumetric; 14% (95% CI: -7.5, -20.1%) for with a sugar concentration threshold (NS) By age group: all 10.2%, adults 6.4%, children 7.7% *p* = 0.91 By SES (NS)	Papers included in meta-analysis: [42, 44, 47, 51, 53, 56, 58, 60, 62–68] Risk of bias assessment classified 8/22 studies high, 6/22 moderate, and 8/22 low quality
**Bergallo et al. **[31]** AMSTAR Rating: Moderate**					
To assess the available evidence on impact of SSB taxation (and other regulatory strategies to reduce SSB) in Latin American countries	Excise tax, and VAT Included naturalistic studies (experimental and modelling excluded) Peer-reviewed studies only	Mexico, Barbados (The Americas)	Narrative	Outcomes included consumption, purchase, or sales of SSB (See data from individual studies Additional file 6)	4 papers: [53, 58, 69, 70] No appraisal of quality of included studies
**Redondo et al. **[32]** AMSTAR Rating: Moderate**					
To synthesize existing evidence related to the impact of SSB taxes on consumption, purchase, or sales	All types of taxes 5 Naturalistic Studies (excluded simulations/modelling studies	USA Mexico (The Americas)	Narrative	Outcomes included SSB sale, purchasing behaviour. (See data from individual studies Additional file 6)	5 papers: [44, 58–60, 62] Quality appraisal addressed 58 elements. Elements not met ranged from 2–6. Elements met ranged from 33–48,
**Afshin et al. **[28]**. AMSTAR Rating: High**					
To quantify the prospective effect of changes in food prices on dietary consumption	Included intervention and PCS on impact of change in price on consumption and adiposity Excluded modelling studies, cross-sectional studies, and laboratory experiments (hypothetical situations)	USA (The Americas)	Study-specific effect sizes were pooled using inverse-variance-weighted random-effect models	Each 10% price increase reduced SSB intake by 7% (95% CI: -3.0, -10.0%)	4 papers: [71–74] Quality scored out of 5 ranged from 3–5
**Nakhimovsky et al. **[29]** AMSTAR rating: High**					
To assess the effectiveness of SSB tax in MIC in comparison to HIC. To assess: (1) prices increase after introduction of excise tax; (2) impact on EI and differences across SES groups	Middle-income countries Any tax or price change on SSB consumption (and preferable body weight outcomes). All SSB categories included Modelling, non-experimental, quasi-experimental or experimental studies	Brazil, Ecuador India Mexico Peru South Africa (African, The Americas)	Standardised estimates for change in SSB intake to kJ/person/day for a 10% price increase	Analysis showed OPE (range: -0.6- to -1.2) equivalent to a 6.0% to 12.0% reduction in consumption with 10% tax. With 10% tax EI reduction of 18.0 (range: 5.0 to 39.0) KJ/ person/day (~ 1.07 (range: 0.3 to 2.3) g sugars /person/day). Assumed linearity between % change in price and intake Reduction in SSB purchase higher (9.1%) in lowest SES tertile than highest (5.5%). OPEs higher among lower income groups (no meta-analysis of these data: see individual studies in Additional file 6)	Nine studies, (three quasi-experimental [59, 75, 76] Six observational & modelling [55, 57, 59, 77–79] Assessed studies against 6 criteria checklist. Mode = 4. Range 1–5 out of 6. Used hierarchy of study design to inform findings
**Backholer et al. **[33]** AMSTAR Rating: Moderate**					
To clarify the differential impact(s) of SSB taxes on SSB purchase, intake, and body weight outcomes by SES	Studies that reported the impact of change in SSB price on purchase, intake, or EI and/or body weight related outcomes according to any marker of SES in HIC	USA UK Ireland New Zealand (European, Western Pacific, The Americas)	Narrative	Outcomes included SSB intake, EI, and body weight outcomes. (See data from individual studies Additional file 6)	8 papers with relevant data: [45, 46, 51, 52, 80–82] Quality of included studies was not appraised
**Niebylski et al. **[39]** AMSTAR Rating: Low**					
To evaluate published evidence to assess the effect of healthier food/beverage subsidies and less food/beverage taxation on diet	Included studies, reviews, and/or predictive models for adults and children in Western Europe, Canada, US, Australia, and New Zealand. Articles assessing tax (any type) effect on nutrition-related health outcomes and consumption of foods including SSB	Canada USA (The Americas)	Narrative	Outcomes included intake of healthier vs. less health (none core) foods, including SSB (See data from individual studies Additional file 6)	15 papers: [29, 37, 46, 52, 71, 73, 78, 83–90] GRADE method used to assess quality of body of evidence
**Thow et al. **[34]** AMSTAR Rating: Moderate**					
To assess the effectiveness of food tax and subsidies policies on consumption	Studies with empirical data on impact of food or nutrient tax (any type) on subsidy on consumption. Modelling and stated preference studies included	USA, Norway, Brazil, France (European, The Americas)	Narrative reporting the range of effect across studies	SSB taxes ranged from 5 to 30%. All showed a reduction in SSB consumption range: 5% to 48%. Overall change in consumption was proportional to the taxes applied SES: four modelled studies from Brazil, Finland and US found higher price sensitivity for low-income households (See data, including for cross price elasticity from individual studies Additional file 6)	Sixteen studies: [38, 42, 51, 52, 56, 73, 78, 81, 83, 87–93] Assessed quality with reference to: 1) Is study prospective 2) Data includes all SSB 3) Price and consumption data from same population 4) Considers potential substitution 5) Based on individual food consumption- yes 6) Study assesses an actual tax – no Scores ranged 1–4/6: 1 study 1/6, 6 studies 2/6, 6 studies 3/6, 1 study 4/6)
**Cabrera Escobar et al. **[35]** AMSTAR rating: Moderate**					
To evaluate the published evidence for impact of SSB taxes or price increases, on consumption levels and obesity, overweight and BMI	Studies with original evidence on the quantitative impact of SSB price changes on consumption or on weight change or BMI Articles in English 2000–2013	USA, Mexico, Brazil, France (European, The Americas)	Meta-analysis on OPE and cross-PED using random effects model	All the results show negative elasticity; OPE -1.3 (95% CI: -1.09, -1.51) Mexico -1.09 (SE 0.20) France -2.21 (SE 0.13) Brazil -0.85 (SE 0.43) Four studies (3 USA, 1 Mexico) reported cross-PED showing increased SSB price increased demand for other beverages, fruit juice 0.39 (95% CI: 0.01, 0.77), and milk 0.13 (95% CI: -0.09, 0.34), and reduced demand for diet drinks − 0.42 (95% CI: -0.63, -1.23) The evidence from LMICs consistent with HIC countries	9 Studies: [42, 52, 56, 77, 78, 83, 87, 88, 91, 94] Quality of studies not assessed
**Maniadakis et al. **[36]** AMSTAR Rating: Moderate**					
To assess the impact of tax policies and price increases on SSB consumption, EI and weight outcomes	Included were original studies in the four categories of: existing data; experiments; survey; and observational studies; – on the association between SSBs prices and taxes and corresponding intake, EI or obesity-related outcomes	USA, UK, Norway, Italy, Denmark, Germany, France, the Netherlands, Mexico, Brazil, Taiwan, Singapore, and Australia (European, SE Asian, Western Pacific, The Americas)	Narrative	Studies (*n* = 9) with data on prices/taxes and PED showed PED ranged from -0.5 to -1.6 with most < 1.0. PED was more regressive towards the lower SES 2 studies with data on SSB tax and EI showed a 10% increase in price/tax reduced EI by up to 50 kcal/day or. 450/month (equivalent to a reduction in sugars of ~ 12.5 g/d and 112.5 g/month)	Included in PED synthesis [71, 85, 95–101] Reported narrative: 27, 50, 51, 55, 73, 79, 80, 83, 90, 94, 98, 106 Quality of studies not assessed
**Powell et al. **[37]** AMSTAR Rating: Moderate**					
To assess PED for SSBs, fast food, and fruits & vegetables, and direct associations of prices/taxes with body weight outcomes	Included studies with US data, original quantitative data on effect of price/tax on consumption or weight change. Studies that assessed demand for product category (not brand) excluded intervention studies	USA (The Americas)	Derived an overall mean estimate of PED of SSB from available SSB estimates for all SSB/sub-categories	Each PED estimate was weighted by its relative consumption share of SSBs based on EI data from individual dietary data for those aged 2 + from the 2007–2008 NHANES Based on 12 available price elasticity studies, the overall OPE was -1.21 (95% CI: -0.71, -3.87) implying that a 10% tax would reduce consumption by 12% and a 20% tax by 24%	11 studies included [51, 52, 73, 81, 83, 87, 88, 96, 101–103] Quality of studies not assessed
**Andreyeva et al. **[38]** AMSTAR Rating: Moderate**					
To estimate effect of price change on consumer demand for major commodity foods, and to explored how altering SSB price alters consumption	Studies with data on SSB reporting on PED	USA (The Americas)	Mean PED estimates and variations in estimates by study design	Average PED 0.79 (95% CI: 0.33, 1.24), range 0.13 to 3.18) based on 14 estimates. A10% tax on SSB reduced consumption by 8–10%	14 studies with data on SSB available from authors

*AVE* Ad valorem equivalent, *BMI* Body mass index, *HIC* High-income countries, *CI* Confidence interval, *EI* Energy intake, *GRADE* Grading of Recommendations, Assessment, Development, and Evaluations, *Kcal* kilocalorie, *MIC* Middle-income countries, *NHANES* National Health and Nutrition Examination Survey, *OPE* Own price elasticity, *PCS* Prospective cohort study, *PED* Price elasticity of demand, *RR* Relative risk, *SE* Standard error, *SES* Socio-economic status, *SSB* Sugar sweetened beverage, *WHO* World Health Organization

^a^ Review Question 1: What are the effects of SSB taxation on SSB on price elasticity of demand/ consumption?

#### Data from high-income countries

Of the systematic reviews with quantitative evidence synthesis, Teng et al. [27], which included data from HIC, and had a high AMSTAR rating, showed that a 10% tax on SSB reduced consumption by 10.0% (95% CI: -5.0, 14.7%) (PED 1.0 (95% CI: -0.5, -1.47)). The analysis by Afshin et al. [28], also with a high AMSTAR rating, was based on pooled data from studies in the US only and showed a 10% tax would reduce consumption by 7.0% (95% CI: -3.0,-10.0%). The review of Powell et al. [37] had a moderate AMSTAR rating and was based on US-based price elasticity studies and showed the overall OPE was -1.21 (95% CI: -0.7, -2.26) implying that a 10% tax would reduce consumption by 7.1–22.6% (average reduction 12.1%), and a 20% tax by 14.2–45.2% (average reduction 24.2%). The review of Andreyeva et al. [38], with a moderate AMSTAR rating and based on US data only, showed, based on mean price elasticity estimates (95% CI: -0.8, 1.0) that a 10% tax would reduce SSB consumption by 8–10%.

#### Data from low- and middle-income countries

The best available evidence synthesis for LMIC was provided by the review of Nakhimovsky et al. [29] which had a Moderate AMSTAR rating. This synthesis standardised data across studies and showed that a 10% tax would reduce consumption by 6–12% (average reduction 9.0%, PED 0.90 (range: -0.6 to -1.2)). The review by Cabrera Escobar et al. [35], which had a moderate AMSTAR rating, included both HIC and LMIC and showed in a meta-analysis of data from the USA, Mexico, Brazil, and France that overall OPE was -1.3 (95% CI: -1.085, -1.509), thus indicating that a 10% tax would reduce consumption by 10.9, 15.1% (average reduction 13.0%). A summary of the data on percent change in SSB consumption is provided in Table 2.

**Table 2:** Summary of quantitative findings of the impact of SSB taxation on percentage change in consumption

**Intervention**	**Percentage change in SSB consumption resulting from taxation**		
	**Author year**	**Number of studies**	**Results**
**10% tax**	Teng et al. 2019 [27]	17	-10.0% (95% CI: -5.0, -14.7%),
**10% price increase**	Afshin et al. 2017 [28]	5	-7.0% (95% CI: -3.0, -10.0%)
**10% tax**	Nakhimovsky et al. 2016 [29]	9	-9.0 (range: -6.0, -12.0%)
**10% tax**	Maniadakis et al. 2013 [36]	17	Range (-5.0, -16.0% (most studies > -10%)
**10% tax**	Powell et al. 2013 [37]	12	-12.1% (95% CI: -7.1, -22.6%)
**20% tax**	Powell et al. 2013 [37]	12	-24.2% (95% CI: -14.2, -45.2%)
**5% to 30% tax**	Thow et al. 2014 [34]	16	Range: (-5.0% to -30.0%) (reduction in SSB consumption proportional to the level of tax applied)

#### Data by age group and SES

Only the review by Teng et al. [27] presented pooled data by age group, finding no significant difference. In an earlier review, Thow et al. [34] reported that the impact of taxes ranging from 5 to 30% on SSB consumption was proportional to tax applied. However, the impact on EI was higher in adults (range: 10.0 to 48.0%) compared with children (range: 5.0 to 8.0%) in due of considerable substitution (e.g. with milk). The systematic reviews of Maniadakis et al. [36], Thow et al. [34] (HIC), and of Nakhimovsky et al. [29] (LMIC) showed SSB taxes to be more regressive in lower income groups. However, difference by SES was not found in the analysis of HIC by Teng et al. [27], which included some studies where a 5% tax led to a greater reduction in consumption in higher SES groups. Further data on the impact of SSB tax on consumption by age and SES can be found in the summary of original studies in Additional file 6.

Only one systematic review provided data on percentage reduction of SSB consumption by type of tax, which varied but was not statistically significant [27]. Average reductions with a 10% tax level were 2.3% (95% CI: -11.2, 7.4) for an *ad valorem* tax, 10.2% (95% CI: -4.1, -15.9%) for volumetric; and 14.0% (95% CI: -7.5%, -20.1%) for a nutrient-based tax based on a sugars concentration threshold.

### Question 2: Systematic reviews that enabled estimation of the impact of SSB tax on sugars consumption

Two included systematic reviews reported data on the impact of SSB tax on EI, which enabled the estimation of the impact of SSB tax on sugars intake [29, 36]. Nakhimovsky et al. [29], by using data from LMIC reported that a 10% tax would reduce EI by a median of 18.0 (range: -5.0, -39.0) KJ/person/day or by 4.3 ( range: -1.2 to -9.3) Kcal/person/day. Based on the Atwater conversion factor (4.0 kcal/ (17.0 kJ)/g sugar) [23], this reduction is equivalent to 1.1 (range: 0.3 to 2.3) g sugars/person/day. Maniadakis, et al. [36] based on data from HIC, estimated that a 10% increase in price/tax would reduce EI by a maximum of 50 kcal/person/day or, 450 per month. This reduction is equivalent to 12.5 g/d and 112.5 g/month decrease in sugars intake. Of the original studies included in the identified systematic reviews, 16 had data that enabled the impact of SSB taxation on intake of sugars to be determined (summarised in Additional file 7). Nine of these studies provided data that enabled the impact of a 20% tax on SSB on free sugars intake to be estimated, showing decreases ranging from 1.8 g to 11.0 g grams sugars/person/day, with the average decrease in LMIC being 4 g/d and in HIC 4.4 g/d.

### Question 3. The effects of decreasing sugars consumption on levels of dental caries

The five included systematic reviews are summarised in Table 3. One review [4] was an update of an earlier review [3]. None of the included systematic reviews provided a meta-analysis pertaining to the impact of amount of sugars intake on the development of dental caries. However, data from included original studies relating to the dose response relationship are summarised in Table 4. The best available data, based on study design came from cohort studies with low risk of bias (RoB). For adults, this was provided by the analysis of Bernabé et al. [104], which showed that for each 10 g intake of total sugars DMFT increased 0.09 over the 11-year follow up period. For children, the best available data for the primary dentition came from the analysis of Turck et al. [12] (Moderate RoB), which showed an increase in dmft of 1.64 between ages 3 and 6 for each 10 g/day sucrose consumption. The best available data for the permanent dentition of children came from the Michigan Study (low RoB) [105, 106] which showed a 1% increase for each 5 g of sugar in children over a three-year follow-up period.

**Table 3:** Summary of systematic reviews with data pertaining to Question 3^a^

**Review objectives**	**Review study inclusion criteria**	**Countries (WHO regions) of included relevant studies**	**Method of evidence synthesis and dental caries related outcomes**	**Included references data on dose response**^b^
**Moynihan and Kelly **[3]**. AMSTAR Rating: High**				
To systematically review all available published data on amount of sugars consumption and levels of dental caries and to report the findings for both adults and children	Studies published 1950- 2011 RCTs, non-randomised trials, cohort, case controlled, cross sectional, ecological, reviews with new data. Studies reporting both amount of sugars and data on dental caries incidence, prevalence, count (DMFT/dmft, DMFS/dmfs, RCI,) Excluded theses, abstracts, and preprints All countries All languages	Argentina, Brazil, Denmark, Finland, Germany, Iceland, Iraq, Japan, Norway, Philippines, South Africa, Sweden, Switzerland, Turkey, UK, USA (African, Eastern Mediterranean, European, Southeast Asia, The Americas)	Meta-analysis and Vote Counting: 42/50 and 5/5 studies in children and adults respectively showed at least one positive sugars-caries association GRADE profile analysis classified evidence as *moderate* quality to support free sugars intake < 10% EI. *Very low* quality evidence to support free sugars < 5% EI. Log-linear relationship between sugars and caries increment between 0.2 kg and 5.0 to 7.5 kg/ person/year in teeth erupted for 7–8 yrs., *r* = 0.8 Meta-analysis indicated large effect size [SMD for DMFT 0.82 (95% CI: 0.67, 0.97)] Evidence for dose response and large size effect from individual studies (see Table 4)	Three papers [105, 107, 108] Quality of body of evidence assessed using GRADE method
**SACN **[40]**. (Grey literature) AMSTAR Rating: Moderate**				
To review the evidence in respect to dietary carbohydrates and oral health	Peer-reviewed PCS and RCT studies in humans. Exposure all categories of dietary CHO including sugars as amount, frequency or dietary sources. Studies to have data to enable HR, RR or OR and measure of uncertainty (CI, SD or *P* value). Clinical assessment of caries. DMFT/dmft, DMFS/dmfs, RCI, visible caries with dentine involvement	UK, USA (WHO regions: European, The Americas)	Narrative account of included studies. Two included studies with data on a quantified amount of sugars intake and dental caries in permanent dentition (see Table 4)	Three papers [106–108] Quality of studies not assessed
**Mahboobi et al. **[41]** AMSTAR Rating: Moderate**				
To assess the association between free sugars and dental caries (incidence and prevalence) in 6- to 12-year-old children from longitudinal evidence	January 1, 2004 and September 22, 2019 Cohort studies only Children 6–12 years only	Finland (WHO Region European)	Narrative report only. Two studies with relevant data to this review: Ruottinen et al. [109] Karjalainen et al. [110] (both positive association between sucrose and caries)	None reporting doses response
**Turck et al. **[12]** AMSTAR Rating: Moderate**				
To deliver a Scientific Opinion UL for sugars based on available data on dental caries (and other diseases and metabolic endpoints). To identify data on dose–response relationship and/or level of intake at which the risk of dental caries is not increased	RCT, non-randomised trials, PCS	Finland, UK USA (WHO regions: European, The Americas)	Reported findings of individual studies (pooling of data across studies was not possible). Notes dose-responses in individual studies in adults [104] and children [106–108, 111] Table 4 New analysis of data from STRIP cohort study showed caries incidence at 6 years was ~ 4 × higher in the highest quartile of sucrose intake at aged 3 yrs. (mean sucrose intake 44 (range: 34.5 to 65.9) g/day) (~ 16 (12 to 24) % E) vs. lowest quartile (mean sucrose intake 15.9 (range: 7.4 to 20.9) g/day) (5.8 (2.6 to 7.6) % E), (OR: 4.32 (95% CI: 1.31, 14.25) Risk increased by 1.64 (95% CI: 1.13, 2.37) for each 10 g/day increase in sucrose intake at 3 years. NS when new caries was expressed as dmft increment Sucrose intake at 12 years and caries increment 12–16 years NS (low participant number) New analysis of data from Iowa Fluoride Study found no relationship between sugars intake over the study period and dental caries between the ages of 5 and 9 years (mixed dentition) after controlling for relevant confounders, Mean intake of sugars high: 114 g/day (range: 53 to 216 g/day)	Three studies (7 papers) with dose response data [104–108, 110, 111] RoB assessed using OHAT (see Table 4
**Moores et al. **[4]** AMSTAR Rating: High**				
To report an update of the systematic review by Moynihan & Kelly [3] of data published 2011–2020 on amount of sugars consumption and dental caries in both adults and children	Epidemiological studies published since November 2011. Reviews with new data. Excluded theses, abstracts, and preprints Included intervention studies altering sugars in-group compared with control with different sugars, and which included information on caries, or comparisons of higher vs lower caries as an outcome. Timescale > / = 1 yr Observational studies reporting quantity of sugars or change in sugars and information on dental caries were included All timescales were included All countries All languages	Australia, Brazil, Denmark Finland, Japan, Kenya, Malaysia, Netherlands, Poland, Puerto Rico, Thailand UK, USA (WHO regions: African, European, SE Asian, Pan Pacific, The Americas)	Vote counting and Harvest Plots supported by narrative. 11/15 studies in children and 6/8 studies in adults reported at least one positive association between sugars and caries. Balance of data supported lower caries with sugars < 10% E and also < 5% E compared with > 5% E 5/7 studies reporting dose response found a positive relationship (adjusted analysis). 2/7 (both with serious RoB) reported no correlation in unadjusted bivariate analysis (unadjusted) see Table 4 for details	Seven studies with doses response data [104, 112–117] Quality of studies assessed using OHAT (see Table 4)

*CHO* Carbohydrate, *CI* Confidence interval, *DMFT/dmft* Decayed missing and filled teeth permanent/primary, *DMFS/dmfs* Decayed missing and filled tooth surfaces permanent/primary, *HR* Hazards ratio, *OR* Odds ratio, *UL* Tolerable Upper Level, *RCI* Root caries index, *RCTs* Randomised controlled trials, *RR* Relative risk, *SACN* Scientific Advisory Committee on Nutrition (UK), *SD* Standard deviation, *SMD* Standard mean difference, *WHO* World Health Organization

^a^ Review Question 3: What are the effects of decreasing sugars consumption on levels of dental caries?

^b^ See Table 4 for details of studies

**Table 4:** Identified studies^a^ with data enabling estimation of the dose response between sugars and dental caries

**Author (year)/** **Country**	**Objectives**	**Study population, sugars exposure and dental outcome**	**Data on dose response**	**Quality appraisal**
**Cohort Studies**				
Rugg-Gunn et al. (1984;1987) [107, 108] England	Cohort study To study dietary habits and the development of dental caries over the same period (2 years)	Children (*n* = 405) aged 12–15 years. Total sugars intake. DMFS increment over 2 years	Sugar intake related to fissure caries increment after adjusting for confounders. Each 30 g/day increased DMFS by 0.36 (95% CI: 0.07, 0.80) over 2-year follow up	Quality appraised by Turuk et al. [12] using OHAT: Tier 2-Probably high RoB Quality of studies not assessed by SACN/ Moynihan and Kelly [3]
Burt et al. (1988; 1994) [105, 111] Szpunar et al. (1995) [106] USA	Cohort study To investigate the relationship between total sugars intake and development of dental caries over 3 years. The study also aimed to relate sugars consumption to the probability of experiencing caries increment	Children (n =) 499 initially aged 10–15 years in Michigan USA. Living in rural non-fluoridated areas Total sugars DMFS over 3 years	Each + 5 g sugars led to a 1% increase in the probability of developing caries over 3-year follow up Higher sugar (% EI and g/d) increased probability of caries on all surfaces but only a higher % EI from sugars significantly increased probability of pit, fissure and aproximal caries	Quality appraised by Turuk et al. [12] using OHAT: Tier 1, probably low RoB Quality of studies not assessed by SACN/ Moynihan and Kelly [3]
Bernabe et al. (2016) [104] Finland	Cohort study Explored the shape of the association of frequency and amount of sugars intake with caries in adults, 2) the relative contribution of frequency and amount of sugars intake to caries levels, and 3) whether the association of frequency and amount of sugars consumption with caries varies according to exposure to fluoride	Finish adults (*n* = 1702) followed up 11 years. Total sugars intake	A linear dose response relationship observed between sugars intake from 13.7 g/d (~ 2% E) to 442 g/d and caries increment. For every 10-g/d sugars intake, DMFT increased by 0.09 (95% CI: 0.02,0.15), *p* = 0.14	Quality appraised by Turuk et al. [12] and Moores et al. [4] using OHAT: Tier 1, probably low RoB
**Cross sectional studies**				
Saw et al. (2012) [116] Malaysia	Cross sectional study To investigate the dietary intake of adults in dental clinic and to evaluate their dental caries experience using DMFT scores. The relationship between total dietary intake and dental caries experience was also investigated	Adults (*n* = 168) 20–59 years Sugars (not defined) g/day and % EI) DMFT by WHO methods	NS correlation between sugars and DMFT index (*r* = 0.055, *P* = 0.476). Correlations assessed using Spearman’s rho correlation test and Pearson correlation test. No apparent adjustment for confounders	Quality appraised by Moores et al. [4] using OHAT: Tier 3, definitely high RoB
Chi et al. (2015) [114] USA (Alaska)	Cross sectional study Evaluated the feasibility of collecting hair samples from Yupik children and tested the association between the hair biomarker-based measure of added sugar intake and tooth decay	Native Alaskan Children Added sugars assessed using biomarker of intake % carious surfaces assessed	Age-adjusted linear regression: 40 g/day increase in added sugars intake associated with a 6.4% absolute increase in the proportion of carious tooth surfaces (95% CI: 1.2% to 11.6%; *P* = 0.02) Log-linear regression model: 40 g/day increase in added sugars associated with a 24.2% relative increase in the proportion of carious tooth surfaces (95% CI: 10.6% to 39.4%; *P* < 0.01)	Quality appraised by Moores et al. [4] using OHAT: Tier 2, Probably high RoB
Mitrakul et al. (2016) [117] Thailand	Cross sectional study To examine the association between dental caries and 2 factors: BMI and diet	Children aged 6–12 years (*n* = 100) Total sugars intake DMFT	Correlation between total sugars and DMFT score: *R* = -0.128, *P* = 0.205. Graphical data show total sugars intake ranged from 0-140 g/day (reported as mg/day, but this was assumed to be g/day)	Quality appraised by Moores et al. [4] using OHAT: Tier 3, definitely high RoB
Rosier et al. (2017) [113] Netherlands	Cross sectional study To comprehensively describe the early stages of caries in a healthy young adult population free of cavities and the relationship with behavioural caries risk factors e.g., diet, oral hygiene	Adults (*n* = 268) Total sugars % EI Enamel caries (ICDAS 1–6)	Correlation coefficient for enamel caries and percent energy from sugars was 0.21 (*P* < 0.01) and for any caries was 0.19 (*P* < 0.01)	Quality appraised by Moores et al. [4] using OHAT: Tier 2, Probably high RoB
Barrington et al. (2019) [112] Australia	Cross sectional study investigating association of overweight/obesity, dental caries experience and diet in a nationally representative sample of Australian adults	15–60 years old (*n* = 4170) Added sugars intake DMFT from National Survey of Adult Oral Health	Positive association between dental caries experience (DMFT), and sugars consumption. Added sugars and total sugars were significantly associated with decayed and missing teeth in adults. Multivariate regression Added sugars • DMFT, 1.0002 (95% CI: 0.999, 1.004), *P* = 0.145 • D, 1.01 (95% CI: 1.00,1.02), *P* < 0.05 • M, 1.01 (95% CI: 1.00, 1.01), *P* < 0.001 • F, 0.999 (95% CI: 0.996, 1.003), *P* = 0.744 Total sugars • DMFT, 1.0003 (95% CI: 0.99, 1.00), *P* = 0.254 • D, 1.003 (95% CI: 1.001, 1.003), *P* < 0.001 • M, 1.001 (95% CI: 1.0004, 1.002), *P* < 0.05 • F, 0.999 (95% CI: 0.999, 1.001), *P* = 0.659	Quality appraised by Moores et al. 2022 using OHAT: Tier 2, Probably high RoB
Ecological studies				
Olczak-Kowalczyk et al. (2016) [115] Poland	Ecological study To assess the relationship between dental caries incidence and general consumption of sucrose in 12-year-old children	Children aged 12 (no further description) Sucrose intake per capita	An increase in sucrose intake by 1 kg/year resulted in an increase in caries frequency by almost 0.92% and an increase in DMFT value by over 0.2%	Quality appraised by Moores et al. [4] using OHAT: Tier 2, Probably high RoB

*CI* Confidence interval, *D3MDT* Decayed (into dentine) missing and filled permanent teeth, *DMFS* Decayed missing and filled permanent tooth surfaces, *EI* Energy intake, *ICDAS* International Caries Detection and Assessment System, *OR* Odds ratio, *RoB* Risk of Bias. OHAT https://ntp.niehs.nih.gov/whatwestudy/assessments/noncancer/riskbias/index.html, *OR* odds ratio

^a^ Original studies identified in systematic reviews addressing Question 3

### Question 4. The likely effect of a 20% volumetric tax on averting dental caries over a 10-year period

Table 5 tabulates the best available data pertaining to the impact of taxation on SSB consumption, sugars intake, and the dose response between amount of sugars intake and development of caries. Estimates for the impact of a 20% volumetric SSB tax on caries development for both HIC and LMIC are presented.

**Table 5:** Summary of the best available evidence and estimated impact of a 20% volumetric tax on the development of dental caries over a ten-year period

	**LMIC**	**HIC**
**Question 1: Impact of tax on SSB consumption and PED**	10% tax led to 9.0% reduction PED 0.9	10% tax led to 10.0% reduction PED 1.0
**Question 2: Impact on free sugars consumption**	20% tax reduces intake by 6.0 g/ day^b^ 20% tax reduces intake by 4.0 g/ day^c^	20% tax reduces intake by 6.2 g/day^b^ 20% tax reduces intake by 4.4 g/day^c^
**Question 3: dose response between amount of free sugars and caries development**^a^	Adults: Each 10 g/d increases DMFT by 0.09 over 11 years Children (caries counts). Each 30 g increased DMFS by 0.36 over 3 years Children (caries occurrence) Each 5 g/day increased DMFT by 1.0% over 3 years	
**Question 4: impact of a 20% SSB tax on dental caries over a 10-year period**		
Adults	With a 6 g/d decrease DMFT is reduced by 0.048 With a 4.0 g/d decrease DMFT is decrease by 0.032	With a 6.2 g/day decrease DMFT is reduced by 0.049 With a 4.4 g/d decrease DMFT is reduced by 0.035
Children (caries counts)	With a 6 g/d decrease, DMFS is reduced by 0.24 With a 4.0 g/d decrease, DMFS is reduced by 0.16	With a 6.2 g/d decrease DMFS is reduced by 0.25 With a 4.4 g/day decrease, DMFS is reduced by 0.18
Children (caries occurrence)	With a 6 g/d decrease, caries occurrence is reduced by 4.00% With a 4 g decrease, caries occurrence is reduced by 2.67%	With a 6.2 g/d decrease, caries occurrence is reduced by 4.13% With a 4.4 g decrease, caries occurrence is reduced by 2.93%

*DMFT* Decayed, missing and filled teeth, *DMFS* Decayed missing and filled (tooth) surfaces, *HIC* High-income countries, *LMIC* Low middle income countries, *PED* Price elasticity of demand, *SSB* Sugar-sweetened beverage

^a^ Available data on the dose response between intake of sugars and development of dental caries are from high-income countries only

^b^ Based on range of intake from Question 2 and Ooi et al. [11] for mean consumption in high-income countries (312.3 ml/d) and middle-income countries (334.4 ml/d)

^c^ Based on average values for HIC and LMIC from original studies identified in included systematic review (Additional file 7)

## Discussion

Through an umbrella review of the best available evidence, the findings of this study suggest that over a ten-year period, a 20% volumetric tax to SSB would reduce the per capita caries count (number of teeth affected by caries) in adults in both HIC and LMIC by approximately 0.03. In children the per capita caries count (number of tooth surfaces affected by caries) would reduce by 0.16 and 0.18, and the caries occurrence by 2.7% and 2.9% in LMIC and HIC respectively.

It has been recognised that no single action will be effective in reducing sugars intake to recommended threshold levels and that this is unlikely to be achieved by interventions that rely on individuals changing behaviour alone [118]. This umbrella review has indicated that SSB taxation alone would have a modest impact on disease levels. Moreover, these reductions in dental disease would likely have notable cost benefits due to the high direct costs incurred in treating dental caries and the indirect costs associated with the disease [119].

### Findings in context of previous findings

Most modelling studies of the effect of SSB taxation on caries have reported on the impact on consumption of SSB but not reported the impact on quantitative measures of dietary sugars consumption per se [18–20]. However, Schwendicke et al. [17] using consumption data from the German National Nutrition Survey indicated a 20% tax had variable impacts across gender and SES groups. The most affect sugars intake being in lower income males (up to an average of 13.7 g/day in males aged 20–29 and 5.7 g/day in females aged 15–19 years). Three [29, 34, 36] of the four [27, 29, 34, 36] systematic reviews identified in this umbrella review that included analyses by SES also showed taxes to be more regressive in lower SES groups.

It has been suggested that taxes are most effective when price change is passed to the consumer [8]. However, few data were identified on the impact of different types of taxation. Only one high quality systematic review showed that nutrient based taxes were most effective, an observation also noted by the WHO [120]. Nutrient based taxes are not, however, always passed to the consumer; SSB taxation in the UK [121], which was a tiered system, based on different sugars thresholds, drove product reformulation to lower the sugars content of drinks available for purchase. The UK SSB has thus resulted in a dual benefit of lowering sugars of products available and deterring consumers from buying drinks with higher sugars content.

### Limitations

Despite the advantage of umbrella reviews in being a method of review to capture large amounts of evidence in a short time frame, there are several limitations to be acknowledge. First, despite a broad search strategy, potentially relevant studies may have been omitted for example the databases selected did not include all databases available in LMIC e.g. African Index Medicus (AIMS), Index, Medicus for the Eastern Mediterranean Region (IMEMR). Moreover, the process of screening, data extraction and analysis take time meaning that any relevant systematic reviews published after the search cut-off date and before publication of the umbrella review will be omitted. In this review, screening, extracting and quality appraisal of systematic reviews were carried out independently in duplicate. However, inadvertent systematic error during selection, appraisal or extraction cannot be ruled out. Umbrella reviews, by nature, are subject to limited coverage of evidence because if a study has not been included in a published systematic review, an umbrella review will not include it. In extracting data from this umbrella review, the estimates for caries reduction for LMIC may be conservative because the identified data on the dose response between sugars intake and development of dental caries came exclusively HIC. Sugars exposure may have a greater effect on caries in populations with less exposure to fluoride [122] and increased likelihood of undernutrition [123]. This umbrella review identified systematic reviews with data from most regions of the world, including the African, European, Southeast Asian, Western Pacific and The Region of the Americas. The data sources used in included systematic reviews covered a broad range of databases including those capturing publications from both HIC and LMIC for example Web of Science, SciElo and LILACS, However, some database capturing data from LMIC e.g. the African Index Medicus, Index Medicus for the Eastern Mediterranean Region were not searched by any review. Future systematic and umbrella reviews should select databases to ensure optimum geographic representation and to ensure all available data from LMIC are captured.

Most systematic reviews reported the impact of SSB taxation on EI with few studies reporting the direct impact on intake of sugars. Restricting included data to original studies identified in the included systematic reviews only may have missed some original data on the impact of taxation on sugars intake. However, to conduct a systematic review of original studies was beyond the scope of this analysis. In the present analysis, Atwater Factors were used to derive intake of sugars from reported changes in EI. This approach assumes that any change in EI is accounted for by a change in intake of sugars, which would underestimate the impact on sugars in scenarios where SSB were replaced with drinks containing energy from nutrients other than sugars (e.g., with milk substitution). For example, although Fletcher et al. [42] reported no impact of taxation on the intake of energy, due to substitution with other sources of energy, this does not equate to no reduction in intake of sugars.

In addition to data from the original studies on the average effect of SSB taxation on sugars intake, published pooled estimates of the mean consumption for HIC and for LMIC [11] were also used to estimate the impact on sugars intake. However, it must be noted that there is considerable variation in SSB intake between HIC and LMIC, which was not captured in this analysis.

### Future research

Based on published data, this umbrella review has provided an indication of the reduction in dental caries that might result from SSB taxation in HIC and LMIC. Further cost-effective analysis based on these data will determine the likely impact on cost benefit to healthcare services in LMIC and HIC. The current study has also identified few data from LMIC exploring the dose response between amount of sugars intake and development of dental caries showing that identified data from LMIC pertaining to amount of sugars and dental caries are cross sectional. There is a need for more, well-conducted cohort studies, especially from LMIC, to explore the dose–response relationship between amount of sugars intake and development of dental caries.

## Conclusion

Through an umbrella review, the best available evidence pertaining to the impact of SSB taxation on sugars intake and levels of dental caries in both HIC and LMIC has been identified. Evidence indicates a 20% tax would reduce sugars intake by 20.0% in HIC and 18.0% in LMIC, and per capita sugars intake by 4.0 g or more a day. This one intervention alone has a modest positive impact on oral health by reducing caries counts in both adults in both HIC and LMIC by 0.03, and caries prevalence in children by 2.7% and 2.9% for HIC and LMIC respectively.

## Supplementary Information

**Additional file 1.** Search Strategies.

**Additional file 2.** Excluded studies.

**Additional file 3.** Data extraction forms for included systematic reviews addressing Questions 1 and 2.

**Additional file 4.** Data extraction forms for included systematic reviews addressing Question 3.

**Additional file 5.** AMSTAR results for included systematic reviews and evidence syntheses.

**Additional file 6.** Summary of original studies identified in included reviews addressing Question 1.

**Additional file 7.** Details of original studies with data on sugars intake.

## Data Availability

All data generated or analysed during this study are included in this published article [and its supplementary information files].

## Abbreviations

AMSTAR: A Measurement Tool to Assess Systematic Reviews
CI: Confidence interval
CINAHL: Cumulated Index to Nursing and Allied Health Literature
CPE: Cross price elasticity
DMFS: Decayed missing and filled tooth surfaces (permanent teeth)
dmfs: Decayed missing and filled tooth surfaces (primary teeth)
DMFT: Decayed missing and filled permanent teeth
dmft: Decayed missing and filled primary teeth
EI: Energy intake
G: Grams
HIC: High-income countries
JBI: Joanna Briggs Institute
Kcal: Kilocalorie
Kj: Kilojoule
LMIC: Low- and middle-income countries
MJ: Mega-joule
NCDs: Non-communicable diseases
OPE: Own price elasticity
PED: Price elasticity of demand
PRISMA: Preferred Reporting Items for Systematic Reviews and Meta-Analyses
PROSPERO: International Prospective Register of Systematic Reviews
RoB: Risk of bias
SSB: Sugar-sweetened beverages
UK: United Kingdom
WHA: World Health Assembly
WHO: World Health Organization
