# Supplementary material for: Effect of sugar-sweetened beverage taxation on sugars intake and dental caries: an umbrella review of a global perspective
Source: BMC Public Health. 2023 May 27;23:986. doi: 10.1186/s12889-023-15884-5 (PMC10224604; doi:10.1186/s12889-023-15884-5)
Supplement: Supplementary file 1 — Additional file 1. Search Strategies. [file 12889_2023_15884_MOESM1_ESM.pdf]

Additional file 1: Search Strategies

a) **Review 1 (Questions 1 and 2)**

**Medline**	(exp sugar-sweetened beverage/ or exp sweetened drink/ or exp sugar-added beverage/ or exp carbonated beverage/ or exp soft drink / or (sugar-sweetened beverage$ or carbonate beverage$).ti,ab.) AND (exp taxation or tax*) AND (systematic review or meta-analy* or metaanaly* or systematic literature review or meta synthesis or metasynthesis)
**Scopus**	(TITLE-ABS-KEY (sugar-sweetened beverage*) OR TITLE-ABS-KEY (sugar-sweetened beverages*) OR TITLE-ABS-KEY (sugar-added beverage*) OR TITLE-ABS-KEY (sweetened drink*) OR TITLE-ABS-KEY (soft drink*) OR TITLE-ABS-KEY (carbonated beverage*)) AND (TITLE-ABS-KEY (taxation) OR TITLE-ABS-KEY (taxation*) OR TITLE-ABS-KEY (tax*)) AND (TITLE-ABS-KEY (systematic review) OR TITLE-ABS-KEY (systematic*) OR TITLE-ABS-KEY (metaanalys*) OR TITLE-ABS-KEY (meta-analys*))
**EMBASE**	('sugar-sweetened beverage':ab,ti OR 'sugar-added beverage':ab,ti OR 'sweetened drinks':ab,ti OR 'carbonated beverages':ab,ti OR soft drinks':ab,ti) AND ('taxation':ab,ti OR 'tax':ab,ti) AND ('meta analysis'/de OR 'systematic review'/de)
**Web of science**	((ALL=('sugar sweetened beverages' OR 'sugar added beverages':ab,ti OR 'sweetened drinks':ab,ti OR 'carbonated drinks':ab,ti)) AND ALL=('taxation' OR 'tax':ab,ti)
**Cochrane library**	("sugar sweetened beverages":ti,ab,kw OR "sugar-sweetened beverages":ti,ab,kw OR "sugar-added beverages":ti,ab,kw) AND ("taxation":ti,ab,kw OR "tax":ti,ab,kw OR "price change":ti,ab,kw OR "fiscal policy":ti,ab,kw) AND ("systematic review":ti,ab,kw OR "meta-analysis":ti,ab,kw)
**Dentistry & Oral sciences Resourc**	((DE "SUGAR-sweetened beverage" OR DE "SUGAR-sweetened beverages" OR DE "SUGAR-added beverage" OR DE "SWEETENED drink" OR DE "SOFT drink" OR DE "CARBONATED beverage") AND (DE "TAX" OR DE "TAXATION") AND (DE "META-analysis" OR DE "SYSTEMATIC reviews"))
**CINAHL**	(MH "sugar-sweetened beverages" OR MH "sugar-sweetened beverage" OR MH "sugar-added beverage" OR MH "sweetened drink" OR MH "soft drink" OR MH "carbonated beverage") AND (MH "taxation" OR MH "taxation" OR MH "tax" OR MH "tax") AND (MH "systematic review" OR MH "systematic review" OR MH "meta-analysis" OR MH "meta-analysis")

b) **Review 2 (Question 3)**

**Medline**	(exp dental caries/ or exp DMF index/ or (dmf or dmft or dmfs or dft or deft or defs or caries or tooth decay or dental decay).ab,ti.) AND (exp dietary sugars/ or exp monosaccharides/ or exp oligosaccharides/ or exp disaccharides/ or exp candy/ or exp honey/ or exp molasses/ or exp carbonated beverages/ or exp diet, cariogenic/ or (sweet$ adj3 (food$ or drink$ or beverage$ or diet$)).ti,ab. or (juice$ adj3 (fruit$ or apple$ or orange$ or grape$)).ti,ab. or (sugar$ or syrup* or honey or sucrose* or glucose* or fructose* or lactose* or maltose* or galactose* or soft drink* or fizzy drink* or carbonated drink* or carbonated beverage* or confectionary or candy or sweet*).ti,ab.) AND (systematic review or meta-analy* or metaanaly* or systematic literature review or meta synthesis or metasynthesis)

**Scopus**	(TITLE-ABS-KEY ("dental caries" OR "oral health" OR dmf* OR dft OR def* OR caries OR "dental health" OR "dental status")) AND (TITLE-ABS-KEY(dietary sugars OR monosaccharides OR oligosaccharides OR disaccharides OR candy OR honey OR molasses OR "carbonated beverage" OR "cariogenic diet")) AND (TITLE-ABS-KEY (sweet* W/3 food* OR drink* OR beverage* OR diet* OR juice*)) AND (TITLE-ABS-KEY (sugar* OR syrup* OR honey OR sucrose* OR glucose* OR fructose* OR lactose* OR maltose* OR galactose* OR "soft drink*" OR "fizzy drink*" OR "carbonated drink*" OR "carbonated beverage*" OR confectionary OR candy OR sweet*))
**EMBASE**	'dental caries' OR 'oral health':ab,ti OR dmf:ab,ti OR dmft:ab,ti OR dmfs:ab,ti OR dft:ab,ti OR deft:ab,ti OR defs:ab,ti OR caries:ab,ti OR 'dental health':ab,ti OR 'dental status':ab,ti AND dietary sugars OR monosaccharides OR oligosaccharides OR disaccharides OR candy OR honey OR molasses OR 'carbonated beverage' OR 'cariogenic diet' OR (('sweet$ adj3 ':ab,ti AND (food$:ab,ti OR drink$:ab,ti OR beverage$:ab,ti OR diet$:ab,ti) AND .ti,ab:ab,ti OR ('juice$ adj3 ':ab,ti AND (fruit$:ab,ti OR apple$:ab,ti OR orange$:ab,ti OR grape$:ab,ti))) AND .ti,ab:ab,ti) OR sugar$:ab,ti OR syrup*:ab,ti OR honey:ab,ti OR sucrose*:ab,ti OR glucose*:ab,ti OR fructose*:ab,ti OR lactose*:ab,ti OR maltose*:ab,ti OR galactose*:ab,ti OR 'soft drink*':ab,ti OR 'fizzy drink*':ab,ti OR 'carbonated drink*':ab,ti OR 'carbonated beverage*':ab,ti OR confectionary:ab,ti OR candy:ab,ti OR sweet*:ab,ti AND ('demineralization'/dm OR 'dental caries'/dm) AND ('meta-analysis'/de OR 'systematic review'/de)
**Web of science**	((ALL=('dental caries' OR 'oral health':ab,ti OR dmf:ab,ti OR dmft:ab,ti OR dmfs:ab,ti OR dft:ab,ti OR deft:ab,ti OR defs:ab,ti OR caries:ab,ti OR 'dental health':ab,ti OR 'dental status':ab,ti)) AND ALL=(dietary AND sugars OR monosaccharides OR oligosaccharides OR disaccharides OR candy OR honey OR molasses OR 'carbonated beverage' OR 'cariogenic diet' OR (('sweet$ adj3 ':ab,ti AND (food$:ab,ti OR drink$:ab,ti OR beverage$:ab,ti OR diet$:ab,ti) AND .ti,ab:ab,ti OR ('juice$ adj3 ':ab,ti AND (fruit$:ab,ti OR apple$:ab,ti OR orange$:ab,ti OR grape$:ab,ti))) AND .ti,ab:ab,ti) OR sugar$:ab,ti OR syrup*:ab,ti OR honey:ab,ti OR sucrose*:ab,ti OR glucose*:ab,ti OR fructose*:ab,ti OR lactose*:ab,ti OR maltose*:ab,ti OR galactose*:ab,ti OR 'soft drink*':ab,ti OR 'fizzy drink*':ab,ti OR 'carbonated drink*':ab,ti OR 'carbonated beverage*':ab,ti OR confectionary:ab,ti OR candy:ab,ti OR sweet*:ab,ti))
**Cochrane library**	("dental caries":ti,ab,kw OR "oral health":ti,ab,kw OR "dmf":ti,ab,kw OR "dmft":ti,ab,kw) AND ("dietary sugars":ti,ab,kw OR "monosaccharides":ti,ab,kw OR "oligosaccharides":ti,ab,kw OR "carbonated beverages":ti,ab,kw) AND ("systematic review":ti,ab,kw OR "meta-analysis":ti,ab,kw)
