# Supplementary material for: Effect of sugar-sweetened beverage taxation on sugars intake and dental caries: an umbrella review of a global perspective
Source: BMC Public Health. 2023 May 27;23:986. doi: 10.1186/s12889-023-15884-5 (PMC10224604; doi:10.1186/s12889-023-15884-5)
Supplement: Supplementary file 2 — Additional file 2. Excluded studies. [file 12889_2023_15884_MOESM2_ESM.pdf]

Additional file 2: Excluded studies

a) **Review 1 (Questions 1 & 2)**

**Wrong publication type (n = 7)**
1. Backholer K, Sarink D, Beauchamp A, Keating C, Loh V, Ball K, Martin J, and Peeters A. The impact of a tax on sugar-sweetened beverages according to socio-economic position: a systematic review of the evidence. *Public Health Nutr* 19: 3070-3084, 2016.
2. Carmona D. Examining the effectiveness of sugar-sweetened beverage taxation on chronic non-communicable oral and systemic diseases. *Ontario Dentist* 97: 26-30, 2020.
3. Jain V, Crosby L, Baker P, and Chalkidou K. Distributional equity as a consideration in economic and modelling evaluations of health taxes: A systematic review. *Health Policy* 124: 919-931, 2020.
4. Lenkinski L. Unintended consequences. *Ontario Dentist* 95:16-16, 2018.
5. Maniadakis N, Kapaki V, Damianidi L, and Kourlaba G. A systematic review of the effectiveness of taxes on nonalcoholic beverages and high-in-fat foods as a means to prevent obesity trends. *Clinicoecon Outcomes Res* 5: 519-543, 2013.
6. Nakhimovsky SS, Feigl AB, Avila C, O'Sullivan G, Macgregor-Skinner E, and Spranca M. Taxes on sugar-sweetened beverages to reduce overweight and obesity in middle-income countries: a systematic review. *PLoS One* 11: e0163358, 2016.
7. Welsh JA, Lundeen EA, and Stein AD. The sugar-sweetened beverage wars: public health and the role of the beverage industry. *Curr Opin Endocrinol Diabetes Obes* 20: 401-406, 2013.
**Wrong study design (n = 9)**
8. Alhareky M, Bedi S, AlMulhim A, El Tantawi M, Farooqi FA, and AlHumaid J. Impact of sugar tax on sugar-sweetened beverage consumption among Saudi schoolchildren. *Oral Health Prev Dent* 19: 189-194, 2021.
9. Gortmaker SL, Wang YC, Long MW, Giles CM, Ward ZJ, Barrett JL, Kenney EL, Sonneville KR, Afzal AS, Resch SC, and Cradock AL. Three interventions that reduce childhood obesity are projected to save more than they cost to implement. *Health Aff (Millwood)* 34: 1932-1939, 2015.
10. Kirkpatrick SI, Raffoul A, Maynard M, Lee KM, and Stapleton J. Gaps in the evidence on population interventions to reduce consumption of sugars: a review of reviews. *Nutrients* 10, 2018.
11. Lee JY and Giannobile WV. Taxes on sugar-sweetened beverages: a strategy to reduce epidemics of diabetes, obesity, and dental caries? *J Dent Res* 95: 1325-1326, 2016.
12. Lee Y, Mozaffarian D, Sy S, Liu J, Wilde PE, Marklund M, Abrahams-Gessel S, Gaziano TA, and Micha R. Health impact and cost-effectiveness of volume, tiered, and absolute sugar content sugar-sweetened beverage tax policies in the United States: a microsimulation study. *Circulation* 142: 523-534, 2020.
13. Parr AM. Cariology: Why is the 2018 soft drinks levy important to dentistry?: George Warman Publications London, p. 8-11, 2021.
14. Sharma A, Hauck K, Hollingsworth B, and Siciliani L. The effects of taxing sugar-sweetened beverages across different income groups. *Health Econ* 23: 1159-1184, 2014.
15. Tebbutt J. Oral health: sugar tax doubts. *Br Dent J* 224: 200, 2018.
16. Thow AM, Downs SM, Mayes C, Trevena H, Waqanivalu T, and Cawley J. Fiscal policy to improve diets and prevent noncommunicable diseases: from recommendations to action. *Bull World Health Organ* 96: 201-210, 2018.
**Wrong intervention (n = 9)**

17. Alcaraz A, Pichon-Riviere A, Palacios A, Bardach A, Balan DJ, Perelli L, Augustovski F, and Ciapponi A. Sugar sweetened beverages attributable disease burden and the potential impact of policy interventions: a systematic review of epidemiological and decision models. *BMC Public Health* 21: 1460, 2021.
18. Barlow P, McKee M, Basu S, and Stuckler D. The health impact of trade and investment agreements: a quantitative systematic review and network co-citation analysis. *Global Health* 13: 13, 2017.
19. Mizdrak A, Scarborough P, Waterlander WE, and Rayner M. Differential responses to food price changes by personal characteristic: a systematic review of experimental studies. *PLoS One* 10: e0130320, 2015.
20. Olm M, Stark RG, Beck N, Röger C, and Leidl R. Impact of interventions to reduce overnutrition on healthcare costs related to obesity and type 2 diabetes: a systematic review. *Nutr Rev* 78: 412-435, 2020.
21. Pfinder M, Heise TL, Hilton Boon M, Pega F, Fenton C, Griebler U, Gartlehner G, Sommer I, Katikireddi SV, and Lhachimi SK. Taxation of unprocessed sugar or sugar-added foods for reducing their consumption and preventing obesity or other adverse health outcomes. *Cochrane Database Syst Rev* 4: Cd012333, 2020.
22. Ricomini Filho AP, Chávez BA, Giacaman RA, Frazão P, and Cury JA. Community interventions and strategies for caries control in Latin American and Caribbean countries. *Brazilian oral research* 35, 2021.
23. Valenzuela MJ, Waterhouse B, Aggarwal VR, Bloor K, and Doran T. Effect of sugar-sweetened beverages on oral health: a systematic review and meta-analysis. *Eur J Public Health* 31: 122-129, 2021.
24. von Philipsborn P, Stratil JM, Burns J, Busert LK, Pfadenhauer LM, Polus S, Holzapfel C, Hauner H, and Rehfuess E. Environmental interventions to reduce the consumption of sugar-sweetened beverages and their effects on health. *Cochrane Database of Systematic Reviews*, 2019.
25. von Philipsborn P, Stratil JM, Burns J, Busert LK, Pfadenhauer LM, Polus S, Holzapfel C, Hauner H, and Rehfuess EA. Environmental interventions to reduce the consumption of sugar-sweetened beverages: abridged cochrane systematic review. *Obes Facts* 13: 397-417, 2020.
**Wrong outcomes (n = 9)**
26. Bes-Rastrollo M, Sayon-Orea C, Ruiz-Canela M, and Martinez-Gonzalez MA. Impact of sugars and sugar taxation on body weight control: A comprehensive literature review. *Obesity (Silver Spring)* 24: 1410-1426, 2016.
27. Claudy M, Doyle G, Marriott L, Campbell N, and O'Malley G. Are sugar-sweetened beverage taxes effective? Reviewing the evidence through a marketing systems lens. *Journal of Public Policy & Marketing* 40: 403-418, 2021.
28. Emmert-Fees KMF, Karl FM, von Philipsborn P, Rehfuess EA, and Laxy M. Simulation modeling for the economic evaluation of population-based dietary policies: a systematic scoping review. *Adv Nutr* 12: 1957-1995, 2021.
29. Eykelenboom M, van Stralen MM, Olthof MR, Schoonmade LJ, Steenhuis IHM, and Renders CM. Political and public acceptability of a sugar-sweetened beverages tax: a mixed-method systematic review and meta-analysis. *Int J Behav Nutr Phys Act* 16: 78, 2019.
30. Jain V, Crosby L, Baker P, and Chalkidou K. Distributional equity as a consideration in economic and modelling evaluations of health taxes: a systematic review. *Health Policy* 124: 919-931, 2020.

31. Lobstein T, Neveux M, and Landon J. Costs, equity and acceptability of three policies to prevent obesity: a narrative review to support policy development. *Obes Sci Pract* 6: 562-583, 2020.
32. Mounsey S, Veerman L, Jan S, and Thow AM. The macroeconomic impacts of diet-related fiscal policy for NCD prevention: a systematic review. *Econ Hum Biol* 37: 100854, 2020.
33. Teng A, Snowdon W, Win Tin ST, Genç M, Na'ati E, Puloka V, Signal L, and Wilson N. Progress in the Pacific on sugar-sweetened beverage taxes: a systematic review of policy changes from 2000 to 2019. *Aust N Z J Public Health* 45: 376-384, 2021.
34. Wright A, Smith KE, and Hellowell M. Policy lessons from health taxes: a systematic review of empirical studies. *BMC Public Health* 17: 583, 2017.

b) Review 2 (Question 3)

**Wrong publication type (n = 2)**
1. Dereń K, Weghuber D, Caroli M, Koletzko B, Thivel D, Frelut ML, et al. Consumption of sugar-sweetened beverages in paediatric age: a position paper of the European academy of paediatrics and the European childhood obesity group. *Ann Nutr Metab* 74: 296-302, 2019.
2. Freeman R. Moderate evidence support a relationship between sugar intake and dental caries. *Evid Based Dent* 15: 98-9, 2014.
**Wrong intervention (n = 2)**
3. Hashem KM, He FJ, MacGregor GA. Effects of product reformulation on sugar intake and health-a systematic review and meta-analysis. *Nutr Rev* 77: 181-96, 2019.
4. Ricomini Filho AP, Chávez BA, Giacaman RA, Frazão P, Cury JA. Community interventions and strategies for caries control in Latin American and Caribbean countries. *Braz Oral Res* 35: e054, 2021.
**Wrong exposure (n = 7)**
5. Baghlaf K, Muirhead V, Moynihan P, Weston-Price S, Pine C. Free sugars consumption around bedtime and dental caries in children: a systematic review. *JDR Clin Trans Res* 3: 118-29, 2018.
6. Elamin A, Garemo M, Mulder A. Determinants of dental caries in children in the Middle East and North Africa region: a systematic review based on literature published from 2000 to 2019. *BMC Oral Health* 21:237, 2021.
7. Hancock S, Zinn C, Schofield G. The consumption of processed sugar- and starch-containing foods, and dental caries: a systematic review. *Eur J Oral Sci* 128: 467-75, 2020.
8. Lingström P, Holm AK, Mejàre I, Twetman S, Söder B, Norlund A, et al. Dietary factors in the prevention of dental caries: a systematic review. *Acta Odontol Scand* 61: 331-40, 2003.
9. Teshome A, Muche A, Girma B. Prevalence of dental caries and associated factors in East Africa, 2000-2020: systematic review and meta-analysis. *Front Public Health* 9: 645091, 2021.
10. von Philipsborn P, Stratil JM, Burns J, Busert LK, Pfadenhauer LM, Polus S, et al. Environmental interventions to reduce the consumption of sugar-sweetened beverages and their effects on health. *Cochrane Database Syst Rev* 6: Cd012292, 2019.
11. Zewdu T, Abu D, Agajie M, Sahilu T. Dental caries and associated factors in Ethiopia: systematic review and meta-analysis. *Environ Health Prev Med* 26: 21, 2021.
**Wrong outcomes (n = 3)**

12. Dooley D, Moultrie NM, Sites E, Crawford PB. Primary care interventions to reduce childhood obesity and sugar-sweetened beverage consumption: Food for thought for oral health professionals. *J Public Health Dent* 77: S104-s27, 2017.
13. Erickson J, Sadeghirad B, Lytvyn L, Slavin J, Johnston BC. The scientific basis of guideline recommendations on sugar intake: a systematic review. *Ann Intern Med* 166: 257-67, 2017.
14. Shakir A, Barngkgei I, Godson J, Joury E. Effectiveness of school-based behavioural interventions to improve children's oral health by reducing sugar intake and promoting oral hygiene: a rapid review of randomised controlled trials. *Community Dent Health* 38: 275-83, 2021.
**No quantitative data on sugars intake in relation to caries (n = 2)**
15. Anderson CA, Curzon ME, Van Loveren C, Tatsi C, Duggal MS. Sucrose and dental caries: a review of the evidence. *Obes Rev* 10: 41-54, 2009.
16. Burt BA, Pai S. Sugar consumption and caries risk: a systematic review. *J Dent Educ* 65: 1017-23, 2001.
