# Supplementary material for: Effect of sugar-sweetened beverage taxation on sugars intake and dental caries: an umbrella review of a global perspective
Source: BMC Public Health. 2023 May 27;23:986. doi: 10.1186/s12889-023-15884-5 (PMC10224604; doi:10.1186/s12889-023-15884-5)
Supplement: Supplementary file 3 — Additional file 3. Data extraction forms for included systematic reviews addressing Questions 1 and 2. [file 12889_2023_15884_MOESM3_ESM.pdf]

Additional file 3. Data extraction forms for included systematic reviews addressing Questions 1 and 2

**Study Details:**	
Author/year	**Afshin, A**., et al., The prospective impact of food pricing on improving dietary consumption: a systematic review and meta-analysis. PLoS One, 2017. 12(3): e0172277.
Objective	To quantify the prospective effect of changes in food prices on dietary consumption
Population (age/total number/gender proportions)	Adult/children, total number and gender are not specified
Setting/context (country)	US
SSB tax interventions (types and levels)	% price increase by taxes (10% - 35%)
Description of SSB consumption (amount/price)	% change in consumption (reduction in SSB consumption by 7%)
**Search details:**	
Databases searched	PubMed, Econlit, Embase, Ovid, Cochrane Library, Web of Science, and CINAHL
Inclusion/exclusion criteria	Inclusion: interventional (randomized or nonrandomized) and observational (prospective cohort) studies that (a) assessed the relationship between change in food price and change in dietary consumption or adiposity among generally healthy individuals (children or adults); (b) reported the estimated change in the price; and (c) provided an estimate of the change in dietary consumption or adiposity and a measure of uncertainty for the reported change. Exclusion: modelling studies, cross-sectional studies, and laboratory experiments (hypothetical situations). Studies were also excluded if (a) all price data were collected before 1990, due to the potential changes in the relation between food prices and consumption over time; (b) outcomes did not include diet or adiposity; or (c) for observational studies, only crude (not multivariable adjusted) effect measures were reported
Range of years of included studies	1990-2014
Number of included studies	5 on SSB
Design of studies included	A mix of nonrandomized interventions and prospective cohort studies
Country of origin of included studies	US
**Appraisal:**	
Quality assessment tool used/ratings	No specific tool
How authors considered the quality	5 criteria: study design, assessment of exposure, assessment of outcome, control for confounding, and evidence of selection bias

**Analysis:**	
Method of analysis	Study-specific effect sizes were pooled using inverse-variance-weighted random-effect models
Outcome assessed/type of measurement	% change in consumption of foods/beverages
Results/findings	Each 10% price increase reduced SSB intake by 7% (95%CI = 3-10%)
Significance/direction	
Heterogeneity	Statistically significant heterogeneity was seen for all I^2 values > 90% (Q-test p = 0.470), I^2 = 75% (Q-test p = 0.002), but not I^2 = 45% (Q-test p = 0.158) or I^2 = 0% (Q-test p ≥ 0.470)
Meta-analytic model used	Effect sizes were pooled using inverse-variance-weighted random-effect meta-analysis
RR/OR, 95% CI, P-value	Each 10% price increase reduced SSB intake by 7% (95%CI = 3-10%)
**Comments:**	
AMSTAR rating	High

**Study Details:**	
Author/year	**Andreyeva, T**., M.W. Long, and K.D. Brownell, The impact of food prices on consumption: a systematic review of research on the price elasticity of demand for food. Am J Public Health, 2010. 100(2): 216-22.
Objective	The aim was to provide a comprehensive summary of research on food demand and consumption behaviour in the United States over the past 7 decades, with particular attention to differences in price effects across income levels. The systematic review, reviewed 160 studies with data on the price elasticity of demand for major food categories including SSB to assess mean elasticities by food category and variations in estimates by study design.
Population (age/total number/gender proportions)	All ages
Setting/context (country)	USA only
SSB tax interventions (types and levels)	Original research articles with data on price elasticity of demand.
Description of SSB consumption (amount/price)	
**Search details:**	
Databases searched	PubMed, EconLit, JSTOR, and Google Scholar working papers, dissertations, and US Department of Agriculture (USDA) technical

	reports
Inclusion/exclusion criteria	Excluded estimates from laboratory experiments, which could change real-world price sensitivity among customers.
Range of years of included studies	The 7 decades prior to 2010.
Number of included studies	160
Design of studies included	Original studies working papers, dissertations, and US Department of Agriculture (USDA) technical reports
Country of origin of included studies	USA
**Appraisal:**	
Quality assessment tool used/ratings	Not assessed
How authors considered the quality	Not done
**Analysis:**	
Method of analysis	Narrative
Outcome assessed/type of measurement	Price elasticity
Results/findings	Absolute value of mean price elasticity and 95% CI 0.79 (0.33, 1.24) range 0.13-3.18 based on 14 estimates. A10% tax on SSB reduced consumption by 8-10%
Significance/direction	
Heterogeneity	Not formally assessed
Meta-analytic model used	Not used
RR/OR, 95% CI, P-value	
**Comments:**	
AMSTAR rating	Moderate
**Study Details:**	
Author/year	**Backholer, K.**, et al., The impact of a tax on sugar-sweetened beverages according to socio-economic position: a systematic review of the evidence. Public Health Nutr, 2016. 19(17): 3070-3084.
Objective	To clarify the differential impact(s) of SSB taxes on beverage purchases and consumption, weight outcomes and the amount paid in SSB taxes according to socio-economic position.
Population (age/total number/gender proportions)	Adult/children from low-income households, total number and gender are not specified
Setting/context (country)	Australia
SSB tax interventions (types and levels)	State-level SSB sales taxes in grocery stores and of 4.2% (one study)
Description of SSB consumption (amount/price)	Individual-level SSB consumed per week. Energy intake was reported in modelling studies
**Search details:**	

Databases searched	(Medline via OVID and EMBASE) and grey literature (System for Information on Grey Literature in Europe, the Virtual Library for Public Health, Google Scholar, plus websites and reports from relevant organizations, including those with a health equity focus)
Inclusion/exclusion criteria	If reported on the impact of a change in SSB price on beverage purchase or consumption, energy intake and/or body weight outcomes (or another marker of adiposity) according to any marker of SEP (individual-or area-based) within a high-income country
Range of years of included studies	Database inception - June 2015
Number of included studies	1 on SSB tax and consumption. One on SSB price elasticity.
Design of studies included	Not specified
Country of origin of included studies	USA
**Appraisal:**	
Quality assessment tool used/ratings	Used a checklist derived from two recent reviews of food and beverage pricing studies
How authors considered the quality	7 criteria: prospective study of observed behaviour, evaluation of an actual tax (rather than a hypothetical tax), price linked directly to purchase within the same Population, consideration of product compensation (cross-price elasticity; CPE), long-run input data across time with sufficient variation in prices used to estimate price elasticities (for experimental studies this included data collected over a period of at least one month, for studies using existing data sets on SSB price this included data collected at intervals no less than two months apart for at least 12 months), valid and appropriate country-specific data, reporting of uncertainty around
**Analysis:**	
Method of analysis	No statistical analysis across studies
Outcome assessed/type of measurement	
Results/findings	Only one included study reported SSB consumption (for other outcome was BMI). For the whole population, no association was observed between SSB taxes and overall SSB consumption or mean BMI change. However, among children from low-income families, a 1 percentage point increase in the SSB tax rate (in excess of other food items) resulted in a significant reduction in the total number of SSB consumed of 0.142 SSB/week.<br>In a price elasticity study: with OPE (SE) reported as −2.0 (1.16), −3.47 (0.99), −0.14

	(0.43), −2.95 (0.52) and −1.27 (0.44) for Q1 (lowest-income quintile) to Q5 (highest-income quintile), respectively; however, the difference between quintiles 1 and 5 was not statistically significant (difference in OPE was 0 07 (95% CI −4 71, 4 84)).
Significance/direction	
Heterogeneity	
Meta-analytic model used	
RR/OR, 95% CI, P-value	
**Comments:**	Based on the available evidence, a tax on SSB will deliver similar population weight benefits across socio-economic strata or greater benefits for over socioeconomic position groups. An SSB tax is shown to be consistently financially regressive, but to a small degree.
AMSTAR rating	Moderate

**Study Details:**	
Author/year	**Bergallo, P**., et al., Regulatory initiatives to reduce sugar-sweetened beverages (SSBs) in Latin America. PLoS One, 2018. 13(10): e0205694.
Objective	To describe the regulatory strategies that Latin American countries have adopted to reduce SSB consumption. To assess the available evidence on these new regulatory strategies, their enforcement challenges, and their impacts.
Population<br>(age/total number/gender proportions)	Latin American countries
Setting/context (country)	Argentina
SSB tax interventions<br>(types and levels)	Excise tax (fixed/% of price), value-added tax (VAT) (% of price)
Description of SSB consumption (amount/price)	Drop in consumption, SSB price change
**Search details:**	
Databases searched	SciElo, Redalyc, JSTOR, Medline, BVS and LILACS
Inclusion/exclusion criteria	Using only countries where initial research had shown relevant results, review was limited to academic articles published in peer-reviewed journals, experimental or modelling studies were intentionally excluded
Range of years of included studies	January 2007 - April 2017
Number of included studies	5
Design of studies included	Academic articles published in peer-reviewed journals about operation of new SSB regulations and policy initiatives, have not specified the design of included studies

Country of origin of included studies	Mexico, Barbados, Dominica, Ecuador, Chile
**Appraisal:**	
Quality assessment tool used/ratings	Did not independently evaluate the quality of the studies, because the search aimed at assessing the content and application of the new policy initiatives, experimental or modelling studies were intentionally excluded.
How authors considered the quality	
**Analysis:**	
Method of analysis	No statistical analysis has been performed
Outcome assessed/type of measurement	
Results/findings	One of the studies concluded that the tax had not been fully transferred to prices (at least through December 2014) in rural areas. The other three papers dealing with Mexico's tax policies ($1/litre) evaluate the effects of the tax on consumption of SSBs or junk food. As far as SSB consumption goes, both studies (by the same lead author) concluded that consumption decreased (7.3%) in the first and second year after the implementation of the tax and that the drop in consumption appears to be increasing. Another study (Barbados) showed that, following the implementation of 10% excise tax, the prices of SSBs increased 5.9% in average, whereas prices for non-SSBs remained mostly flat and sales of SSB decreased 4.3(3.6, 4.9)% after accounting for underlying trends.
Significance/direction	
Heterogeneity	
Meta-analytic model used	
RR/OR, 95% CI, P-value	
**Comments:**	
AMSTAR rating	Moderate

**Study Details:**	
Author/year	**Cabrera Escobar, M.A.**, et al., Evidence that a tax on sugar sweetened beverages reduces the obesity rate: a meta-analysis. BMC Public Health, 2013. 13: 1072.
Objective	To evaluate the published evidence for SSB tax or price increases, and their potential impact on consumption levels and effects on obesity, overweight and BMI.
Population (age/total number/gender proportions)	All ages, both men and women
Setting/context (country)	USA, Mexico, Brazil France
SSB tax interventions (types and levels)	Studies performed in the USA, two used tax data and four price data, while the studies

	performed in Mexico, Brazil and France used price data
Description of SSB consumption (amount/price)	Not specified amount or price specifically. It has been mentioned grams of soft drink consumption for one of the studies included.
**Search details:**	
Databases searched	Pubmed/Medline, The Cochrane database of systematic reviews, Google Scholar, EconLit (AEA), National Bureau of Economics Research (NBER), and Research Papers in Economics (RePEc).
Inclusion/exclusion criteria	Articles in English from any country, with original evidence on the quantitative impact of SSB price changes on the consumption of SSBs, consumption of other drinks, or weight loss, obesity or BMI. For the meta-analysis, we excluded articles that did not report standard error or confidence interval on the own-price elasticity and/or cross-price elasticity. Articles that did not clearly define the type of SSBs were also excluded.
Range of years of included studies	January 2000 - January 2013
Number of included studies	9
Design of studies included	Cross sectional or longitudinal studies. There were no intervention studies.
Country of origin of included studies	Six from the USA, and one each from Mexico, Brazil and France
**Appraisal:**	
Quality assessment tool used/ratings	No tool has been mentioned
How authors considered the quality	
**Analysis:**	
Method of analysis	Meta-analysis was preformed to synthesize results for own- and cross-price elasticities using the random effects model
Outcome assessed/type of measurement	
Results/findings	All the results show negative elasticity, which means that an increase in price was associated with a decrease in the demand for SSBs. OPE =-1.3 (-1.085, -1.509).<br>Brazil: two different own-price elasticities, for poor (−1.03) and non-poor (−0.63) (urban and rural area), which suggests that in Brazil, the poor are more price-sensitive than the more affluent.<br>OPE:<br>Mexico -1.085 (SE 0.195)<br>France -2.206 (SE 0.133)<br>Brazil -0.85 (SE 0.434)<br>See table 3 paper for 6 x USA.

	Overall -1.3 (-1.085 - -1.509).<br>The evidence from LMICs consistent with HIC countries
Significance/direction	
Heterogeneity	Has not been specified
Meta-analytic model used	Random effects model
RR/OR, 95% CI, P-value	The results from the meta-analysis show that the pooled elasticity estimate is -1.30 (95% CI: -1.089 – -1.509).
**Comments:**	
AMSTAR rating	Moderate

**Study Details:**	
Author/year	**Itria, A**., et al., Taxing sugar-sweetened beverages as a policy to reduce overweight and obesity in countries of different income classifications: a systematic review. Public Health Nutr, 2021. 24(16): 5550-5560.
Objective	To assess the effect of implemented SSB tax policies on reducing consumption, purchase and sales, overweight and obesity prevalence
Population<br>(age/total number/gender proportions)	All ages, men and women
Setting/context (country)	Brazil
SSB tax interventions<br>(types and levels)	A tax applied to at least one category of non-alcoholic beverages containing added sugars. Taxes could comprise excise taxes, import tariffs, sales taxes or any other taxes applied
Description of SSB consumption<br>(amount/price)	Changes in the consumption of SSB, including any alteration in taxed beverage sales/purchases (as a proxy for consumption data), or dietary intake following the implementation of an SSB tax and these could be reported by volume, energies or consumption frequency
**Search details:**	
Databases searched	Cochrane Library, Embase, LILACS (via Virtual Health Library) and MEDLINE (via PubMed), and Web of Science. Reference lists from eligible studies and systematic reviews were searched for additional relevant studies. Peer-reviewed publications and grey literature (reports and self-published research) were included.
Inclusion/exclusion criteria	Studies based on primary quantitative research, including modelling, non-experimental, quasi-experimental or experimental studies, full text published, written in English or Spanish.

	This review excluded other systematic reviews and meta-analyses as well as qualitative studies, case studies and reports, and letters to the editor.
Range of years of included studies	January 2009 – December 2019
Number of included studies	16 on SSB taxes on the consumption, purchase or sales
Design of studies included	
Country of origin of included studies	Barbados, UK, Ireland, USA, Chile, Australia, Mexico, South Africa, India
**Appraisal:**	
Quality assessment tool used/ratings	A critical appraisal tool established according to previously published reviews of food and beverage pricing studies
How authors considered the quality	Scores ranged from 4 to 5 in HIC, upper-income countries and MIC
**Analysis:**	
Method of analysis	No statistical analysis has been performed
Outcome assessed/type of measurement	
Results/findings	Sales and purchase were evaluated in five studies. Sales decrease of 8.6 ml/capita/week after applying a 10% ad valorem tax in Barbados (time series analysis) and 9.6% following a US$0.67¢/oz. price increase in California (comparison between pre-tax and first year post-tax) were found. Purchase decrease of 15% for a 20% sales tax in the United Kingdom (theoretical simulation models) and 22 % for 1.6 % price increase in Chile (comparison between pre-tax and post-tax) were found, but no statistically significant association was found in children's consumption of SSB for a 4.2 % (mean) state-level sales tax in grocery stores in the USA (comparison between pre-tax and post-tax).<br>According to three references, consumption was reduced by approximately 13% (20% added tax), 20% ($0.01 cent per fluid ounce excise tax) and 40% ($0.015 cents per fluid ounce excise tax), and all studies applied theoretical simulation models. Energy intake was evaluated in most of the studies and showed reductions of: a) 8.8 to 69.9 kJ/d/person; b) 25 kJ for each one-percentage point increase in the soft drink tax; and c) 150 kJ/d in different studies. These studies applied theoretical simulation models except for Silver et al. Three studies based on theoretical simulation models examined the effect of taxes from theoretical simulation models in upper-middle-income countries, and

	all of them showed an association between taxation and SSB purchase.
Significance/direction	
Heterogeneity	
Meta-analytic model used	
RR/OR, 95% CI, P-value	
**Comments:**	
AMSTAR rating	High

**Study Details:**	
Author/year	**Maniadakis, N**., et al., A systematic review of the effectiveness of taxes on nonalcoholic beverages and high-in-fat foods as a means to prevent obesity trends. Clinicoecon Outcomes Res, 2013. 5: 519-43.
Objective	To assess the impact of tax policies and price increases upon the consumption of SSBs on consumption, caloric intake and weight outcomes.
Population<br>(age/total number/gender proportions)	
Setting/context (country)	Greece
SSB tax interventions<br>(types and levels)	Excise or sales taxes
Description of SSB consumption<br>(amount/price)	Different descriptions in different study designs<br>SSB consumption (L/year/capita, per ounces per capita)<br>Energy intake (per capita change in calories per month, total calories per person per month, kcal/d per person)
**Search details:**	
Databases searched	PubMed, Web of Science, Cochrane Library, AgEcon, EconLit, and the National Agricultural Library databases and searches in other potentially relevant internet sources such as Google
Inclusion/exclusion criteria	Original studies including the four types of primary research methods – existing data, experiments, surveys, and observation – that focused on the association between SSBs and HFSSFs prices and taxes and their corresponding consumption or energy intake or obesity-related outcomes.<br>Systematic reviews, meta-analyses, qualitative studies, case studies, case reports, and letters to the editor were excluded. Moreover, only studies published in English with available full text and studies concerning human subjects were included.

Range of years of included studies	1990 – February 2013
Number of included studies	9 demand studies: association between prices and taxes with the consumption of SSBs<br>6 studies: association between beverage/food prices and taxes and energy
Design of studies included	Demand, longitudinal, cross-sectional, modelling, experimental and cohort studies
Country of origin of included studies	USA, UK, Norway, Italy, Denmark, Germany, France, the Netherlands, Mexico, Brazil, Taiwan, Singapore, and Australia
**Appraisal:**	
Quality assessment tool used/ratings	Not specified
How authors considered the quality	
**Analysis:**	
Method of analysis	No statistical analysis has been performed
Outcome assessed/type of measurement	
Results/findings	Demand studies indicated that the price elasticity of demand for beverages is in the range of -0.5 to -1.6 depending on the beverage considered, with most of them falling below 1.0. The studies also pointed out that the negative effects on the consumption by price increases and taxes depend on factors such as the income group, and are more regressive towards the lowest income categories.<br>The caloric effect of a 10% increase in prices or a corresponding imposition of a tax reduces energy intake by a maximum of 50 calories per day, 450 per month, and up to 0.3 kilograms or 1.5 pounds per year
Significance/direction	
Heterogeneity	
Meta-analytic model used	
RR/OR, 95% CI, P-value	
**Comments:**	
AMSTAR rating	Moderate

**Study Details:**	
Author/year	**Nakhimovsky, S.S.**, et al., Taxes on sugar-sweetened beverages to reduce overweight and obesity in middle-income countries: a systematic review. PLoS One, 2016. 11(9): e0163358.
Objective	To assess the effectiveness of an SSB tax in MICs, in comparison to the evidence from high-income countries. Specific objectives were to assess whether, in MICs 1) prices increase after governments impose an excise tax; 2) net intake of excess energy falls across the population, and

	whether the magnitude of change differs across socio-economic groups
Population<br>(age/total number/gender proportions)	Whole population
Setting/context (country)	US
SSB tax interventions<br>(types and levels)	tax pass-through rate, own-PE and cross-Pes (price elasticity)
Description of SSB consumption (amount/price)	kilojoules per person per day (kJ PPPD)<br>Two quasi-experimental studies and one non-experimental study reported change in consumption in volume (litres or millilitres) or kilocalories
**Search details:**	
Databases searched	PubMed,Web of Science,Cochrane Library, AgEcon, EconLit, the National Agricultural Library, and Google Scholar. Finally, the reference lists in the selected studies were checked and leading scholars contacted to identify any additional studies
Inclusion/exclusion criteria	All such studies—modelling, non-experimental, quasi-experimental, or experimental studies, and published journal articles, dissertations, or working papers meeting these criteria were included.<br>This review excluded other systematic reviews and meta analyses as well as qualitative studies, case studies and reports, and letters to the editor.
Range of years of included studies	March 2013 - March 2016
Number of included studies	9
Design of studies included	Observational or modelling studies,
Country of origin of included studies	Brazil, Ecuador, India, Mexico, Peru, South Africa
**Appraisal:**	
Quality assessment tool used/ratings	Using the quality checklist for food and beverage taxes and subsidies studies in a previous systematic review
How authors considered the quality	Based on study design, with the quasi-experimental studies ranked first and highest and the non-experimental studies and modelling studies ranked second and lowest
**Analysis:**	
Method of analysis	No statistical analysis has been performed
Outcome assessed/type of measurement	
Results/findings	Overall, these results showed a consistent negative correlation between price and SSB consumption. Own-PE estimates for SSB products from six studies range from -0.6 to -1.2. Two studies without own-PE estimates also demonstrated a negative relationship between

	the price and consumption of SSB products. The change in kJ/person/day given a 10% price increase, with estimates ranging from 5 to 39 kJ/person/day and a median estimate of 18 kJ/person/day. Changes in SSB product consumption is higher (21–39 kJ/person/day) among studies from Mexico and Ecuador than in other countries (5–18 kJ/person/day). Consumption of SSBs by Socio-economic Group. Of the six studies with relevant estimates, a quasi-experimental study estimated that the reduction in SSB products purchased relative to a counterfactual is higher (9.1%) among the lowest socio-economic third than among the highest (5.5%). Similarly, three non-experimental studies' OPE by quintile detected differences in sensitivity to price change by sub-population, with OPEs generally higher among more vulnerable and lower income groups. In contrast, a non-experimental study and a modelling study grouping socio-economic groups into thirds showed little difference in own-PE estimates across socio-economic groups. One of these studies noted under-sampling among the rural low-income group, which likely weakened its power to detect differences between sub-groups. Overall, these findings suggest that lower socio-economic groups are more responsive to price changes in SSB products compared to other groups in MICs.
Significance/direction	
Heterogeneity	
Meta-analytic model used	
RR/OR, 95% CI, P-value	
**Comments:**	An update of 2013 systematic review by Maniadakis
AMSTAR rating	High

**Study Details:**	
Author/year	**Niebylski, M.L**., et al., Healthy food subsidies and unhealthy food taxation: a systematic review of the evidence. Nutrition, 2015. 31(6): 787-95.
Objective	To explore the potential effectiveness of food subsidies and taxation on healthy population-wide dietary intake
Population (age/total number/gender proportions)	
Setting/context (country)	USA, Canada

SSB tax interventions (types and levels)	
Description of SSB consumption (amount/price)	
**Search details:**	
Databases searched	PubMed, Medline and Cochrane Library databases (June 2003–November 2013) and Google Scholar (June and November 2013)
Inclusion/exclusion criteria	Included are studies, reviews, and/or predictive models for adults and children in Western Europe, Canada, United States, Australia, and New Zealand that assessed the subsidy and/or tax effect on: (1) nutrition related health indicators to include blood pressure, body mass index (BMI), blood lipids or glucose, (2) healthy food purchases (fruits and vegetables) by consumers, and (3) increased consumption of healthier foods, and reduced consumption of unhealthy foods to include SSBs.<br>Those that were not in English, did not involve humans, were based on data previously published, focused strictly on dietary salt, or were not full reports (e.g., abstracts) were excluded. Articles that failed to document any outcomes of interest were also excluded.
Range of years of included studies	Mentioned above
Number of included studies	
Design of studies included	
Country of origin of included studies	
**Appraisal:**	
Quality assessment tool used/ratings	Grading of Recommendations Assessment, Development, and Evaluation (GRADE)
How authors considered the quality	
**Analysis:**	
Method of analysis	No statistical analysis has been performed
Outcome assessed/type of measurement	
Results/findings	
Significance/direction	
Heterogeneity	
Meta-analytic model used	
RR/OR, 95% CI, P-value	
**Comments:**	Reviews have been included in this study, there are data from some individual studies not identified in other reviews i.e. Wang et al 2010
AMSTAR rating	Low

**Study Details:**	

Author/year	**Powell, L.M.**, et al., Assessing the potential effectiveness of food and beverage taxes and subsidies for improving public health: a systematic review of prices, demand and body weight outcomes. Obes Rev, 2013. 14(2): 110-28.
Objective	To assess the price elasticity of demand for sugar-sweetened beverages (SSBs), fast food and fruits and vegetables as well as the direct associations of prices/taxes with body weight outcomes
Population (age/total number/gender proportions)	Retailers, national sample of households, children
Setting/context (country)	US
SSB tax interventions (types and levels)	Elasticity of demand (consumption or purchases) for SSBs
Description of SSB consumption (amount/price)	Sales per capita galloons, kcal, kcal/day, grams, oz./day, times/week
**Search details:**	
Databases searched	Medline, PubMed, Econlit, and PAIS, research reports from the Economic Research Service (ERS) of the US Department of Agriculture
Inclusion/exclusion criteria	(i) used US data; (ii) was a peer-reviewed study (exception for ERS studies); (iii) provided original quantitative evidence on the relationship between prices/taxes/subsidies and consumption (or weight outcomes); (iv) was not an intervention study; (v) was not a pilot study; (vi) assessed demand for product categories (i.e. regular carbonated soda) rather than brands (i.e. Coke or Pepsi); and, (vii) for weight outcomes, contained direct estimates and was not a modelling study that drew on price elasticity estimates to derive simulated impacts on weight.
Range of years of included studies	January 2007 - March 2012.
Number of included studies	14
Design of studies included	Demand system, cross-sectional, Rotterdam model,
Country of origin of included studies	US
**Appraisal:**	
Quality assessment tool used/ratings	Not specified
How authors considered the quality	
**Analysis:**	
Method of analysis	No statistical analysis has been performed
Outcome assessed/type of measurement	
Results/findings	The estimated overall mean price elasticity of demand for SSBs was -1.21. This estimate was based on all 12 available SSB elasticity estimates including those for the aggregated SSB measures and those estimates available for each of the three

	subcategories of SSBs (regular soft drinks, sports drinks, and fruit drinks) where each estimate was weighted by its relative consumption share of SSBs. The mean SSB price elasticity estimates of -1.21 implies that a tax that raises the price of SSBs by 20% would reduce overall consumption of SSBs by 24%.
Significance/direction	
Heterogeneity	
Meta-analytic model used	
RR/OR, 95% CI, P-value	
**Comments:**	Extending previous study by Andreyeva et al, 2010
AMSTAR rating	Moderate

**Study Details:**	
Author/year	**Redondo, M**., I. Hernández-Aguado, and B. Lumbreras, The impact of the tax on sweetened beverages: a systematic review. Am J Clin Nutr, 2018. 108(3): 548-563.
Objective	To synthesize existing evidence related to the impact of taxes on the consumption, purchase, or sales of SSBs.
Population<br>(age/total number/gender proportions)	
Setting/context (country)	Spain
SSB tax interventions<br>(types and levels)	
Description of SSB consumption (amount/price)	
**Search details:**	
Databases searched	MEDLINE through PubMed, the Cochrane Library, Web of Science and Scopus
Inclusion/exclusion criteria	Original evidence on the impact of taxes established either in isolation or in conjunction with other measures on the consumption, sales, or purchase of SSBs. Excluded were editorials, letters to the editor, and any other non-original study, research on other beverages or on the impact of other types of measures (educational, marketing, price variation, etc.), those that measured the impact of taxes through simulations or theoretical estimations (studies based on modelling analysis used to predict the effect of SSB taxes on a population without empirical data), or others that, although being related to taxes or subsidies, did not analyse the impact on the consumption, purchases, or sales of SSBs.

Range of years of included studies	1 January 2011 to 31 December 2017
Number of included studies	17 (naturalistic: 5, experimental: 12)
Design of studies included	Naturalistic experiments (interventions), Virtual or experimental conditions (randomised controlled studies, AB/ABA studies)
Country of origin of included studies	Naturalistic studies: Mexico, US<br>Experimental studies: Canada, US
**Appraisal:**	
Quality assessment tool used/ratings	The TREND (Transparent Reporting of Evaluations<br>with Nonrandomized Designs) statement for studies classified as naturalistic studies and those with an AB or ABA design and the CONSORT (Consolidated Standards of Reporting Trials) for randomized controlled studies
How authors considered the quality	
**Analysis:**	
Method of analysis	
Outcome assessed/type of measurement	Intervention studies: SSB sale, purchase<br>Randomised studies: purchase, energy selection<br>AB/ABA studies:
Results/findings	Naturalistic: purchases or sales of SSBs decreased significantly with taxation amounts of 8% (Berkeley, CA) and 10% (Mexico). One study found no effect on sales of SSBs in 2 states that enacted a 5.5% tax on sodas.<br>Experimental: purchasing behaviour or sales (6 studies; 50.0%) or behavioural intent (6 studies; 50.0%), resulting in a decrease in either purchasing behaviour or sales or intent behaviour with heterogeneity according to the tax rate.
Significance/direction	
Heterogeneity	
Meta-analytic model used	
RR/OR, 95% CI, P-value	
**Comments:**	Outcomes are described as SSB purchasing behaviour or sale.
AMSTAR rating	Moderate

**Study Details:**	
Author/year	**Sobhani, S.R**. and M. Babashahi, Taxation for reducing purchase and consumption of sugar-sweetened beverages: A systematic review. International Archives of Health Sciences, 2019. 6(2): 65-72.
Objective	To assess evidence about the tax impacts on the purchase and consumption

	of SSBs
Population<br>(age/total number/gender proportions)	
Setting/context (country)	
SSB tax interventions<br>(types and levels)	Excise tax, sale tax.
Description of SSB consumption<br>(amount/price)	
**Search details:**	
Databases searched	PubMed, Scopus, ScienceDirect, CENTERAL, and EMBASE
Inclusion/exclusion criteria	Studies were included if they reported on the impact of a change in SSB price on consumption/purchase/sales of high sugar beverages, calorie intake, weight, body mass index, and consumption/purchase/sales by empirical data. Repeated measure panel design-several pre-tests and post-test of the same group or randomized controlled trials were included. The study was based on empirical data, excluding reviews, commentaries, editorials, and modelling study. General food taxes were excluded, but if there are separated information, they were entered into this study. Furthermore, studies without English full text were excluded.
Range of years of included studies	2000 - May 2017
Number of included studies	7
Design of studies included	
Country of origin of included studies	Mexico, USA
**Appraisal:**	
Quality assessment tool used/ratings	
How authors considered the quality	The quality criteria assessed were as follows: (i) prospective study of observed behaviour; (ii) evaluation of an actual tax (rather than a hypothetical tax); (iii) price linked directly to purchase within same population; (iv) consideration of product compensation (cross-price elasticity); (v) long-run input data across time with sufficient variation in prices used to estimate price elasticities (for experimental studies this included data collected over a period of at least 1 month, for studies using existing data sets on SSB price this included data collected at intervals no <2 months apart for at least 12 months), (vi) valid and appropriate country-specific data, and (vii) reporting of uncertainty around price elasticity estimates. All quality criteria were reported for all studies and

	rate each study out of seven reflecting one point for each quality measure
**Analysis:**	
Method of analysis	No statistical analysis has been performed
Outcome assessed/type of measurement	Excise tax ($0.01/oz.) in low-income neighbourhoods in Berkeley versus in the comparison cities of Oakland and San Francisco, California: Consumption of SSBs decreased 21% in Berkeley and increased 4% in comparison cities (P = 0.046).
Results/findings	
Significance/direction	
Heterogeneity	
Meta-analytic model used	
RR/OR, 95% CI, P-value	
**Comments:**	
AMSTAR rating	Moderate

**Study Details:**	
Author/year	**Teng, A.M.**, et al., Impact of sugar-sweetened beverage taxes on purchases and dietary intake: systematic review and meta-analysis. Obes Rev, 2019. 20(9): 1187-1204.
Objective	To systematically identify quantitative studies that evaluated the impact of real world SSB taxes on SSB sales, purchases, and dietary intake before and after the tax, or in a taxed compared with an untaxed jurisdiction, and to combine results by meta-analysis
Population<br>(age/total number/gender proportions)	
Setting/context (country)	New Zealand
SSB tax interventions<br>(types and levels)	A tax applied to at least one category of non-alcoholic beverages containing added sugars. Taxes could comprise excise taxes, import tariffs, sales taxes, or any other taxes applied by a jurisdiction. SSB taxes were generally charged either at a local currency value per litre (i.e., specific or volumetric tax) or as a proportion of the product's value (i.e., ad valorem). Some taxes were based on the sugar content of SSBs and only apply above a defined threshold (e.g., grams of sugar per litre).
Description of SSB consumption<br>(amount/price)	Reported “consumption” and included any change in taxed beverage sales, purchases, or dietary intake following the implementation of a SSB tax and could be reported by volume, calories, or consumption frequency.

	The effect of SSB taxes could be reported as a ratio, difference, percentage change, or tax elasticity. Tax elasticity is the percentage change in consumption for a 1% change in tax.
**Search details:**	
Databases searched	Google Scholar, Medline, Scopus, and EconLit were searched for articles published in any language between database inception (<1980, except for EconLit, which was 1987) and June 8, 2018
Inclusion/exclusion criteria	Eligible inclusions were those that evaluated the impact of a real world SSB tax introduced in a distinct local or central government jurisdiction (e.g., city, region, or nation). Studies evaluating taxes that were applied within a limited setting such as supermarkets, airports, or schools were not eligible, simulation studies and experimental studies were excluded
Range of years of included studies	
Number of included studies	Systematic review (n=22)<br>meta-analysis (n=17)
Design of studies included	
Country of origin of included studies	US, Catalonia, Chile, France, Mexico, Finland, Hungary
**Appraisal:**	
Quality assessment tool used/ratings	Grading of Recommendations Assessment, Development and Evaluation (GRADE) risk of bias questions, and the Critical Appraisal Skills Programme (CASP) appraisal checklist for cohort studies.
How authors considered the quality	study design, inclusion of a control, untaxed beverage outcomes reported, representativeness of the taxed population, same outcome in comparison groups, objectivity of the outcome, correct classification of taxed and untaxed beverages, same individuals or stores over time, follow-up time points, adjustment for major confounders, accounts for changes in portion size, and reporting of any other health policies that were introduced with the SSB tax
**Analysis:**	
Method of analysis	
Outcome assessed/type of measurement	
Results/findings	Two-thirds of studies measured sales or purchasing outcomes (n = 15) and one-third measured dietary intake (n = 7). Of the outcomes included in the meta-analysis, 11 out of 17 reported significant reductions in SSB sales, purchases, or dietary intake. Results from

	Mexico reported significantly greater consumption declines in lower income households. The two studies from Chile reported greater consumption declines in high-income groups, although this association was statistically significant in only one study. In the main meta-analysis, the equivalent of a 10% increase in SSB tax was associated with a decline in purchases and dietary intake of 10.0% (95% CI: −5.0% to −14.7%, n = 17 studies/6 jurisdictions) based on pre–post intervention comparisons and/or comparisons to an untaxed control jurisdiction. This corresponded to a tax elasticity of −1.00 (95% CI: −0.50 to −1.47).
Significance/direction	
Heterogeneity	There was a large amount of heterogeneity in results between jurisdictions (I2 = 97%) but generally low levels of heterogeneity within jurisdictions (I2 ≤ 25% in Mexico, Berkeley, and other US jurisdictions) with the exception of Chile (I2 = 95%).
Meta-analytic model used	The summary measure was a risk ratio or rate ratio (RR) scaled for a 10% sized tax.
RR/OR, 95% CI, P-value	
**Comments:**	
AMSTAR rating	High

**Study Details:**	
Author/year	**Thow, A.M.**, S. Downs, and S. Jan, A systematic review of the effectiveness of food taxes and subsidies to improve diets: understanding the recent evidence. Nutr Rev, 2014. 72(9): 551-65.
Objective	To assess effectiveness, the effect of tax and subsidy policies on consumption, which is the basis for the effect of taxes and subsidies on body weight and chronic disease
Population (age/total number/gender proportions)	Adults, children
Setting/context (country)	Australia
SSB tax interventions (types and levels)	Tax as a percentage of price
Description of SSB consumption (amount/price)	
**Search details:**	
Databases searched	MEDLINE, Web of Knowledge, EconoLit, and Business Source Premier academic databases and Google Scholar

Inclusion/exclusion criteria	1) The study was based on empirical data, excluding reviews, commentaries, and editorials; 2) study examined a tax or subsidy targeted to influence the price of a specific food product or nutrient (i.e., general agricultural subsidies and general food taxes were excluded); and 3) study assessed the effect of the tax on food and/or nutrient consumption. Modelling and stated preference studies were included
Range of years of included studies	January 2009 - March 2012
Number of included studies	16
Design of studies included	Modelling studies
Country of origin of included studies	USA, Norway, Brazil, France
**Appraisal:**	
Quality assessment tool used/ratings	The assessment tool was based on the Cochrane hierarchy of evidence
How authors considered the quality	1) The strength of the methodology used to collect data, with respect to whether behaviour was observed prospectively rather than being observed retrospectively, self-reported, or imputed;<br>2) The strength of analysis, with respect to whether key variables are linked in the dataset – namely, purchasing behaviour or consumption, and price;<br>3) The completeness of the dataset, with respect to the inclusion of foods other than the target food (allowing measurement of substitution responses) and a high degree of specificity of foods and nutrients;<br>4) The feasibility of implementation, with respect to whether the fiscal intervention was an actual tax or subsidy (i.e., implemented by a governmental body).
**Analysis:**	
Method of analysis	No statistical analysis has been performed
Outcome assessed/type of measurement	
Results/findings	Sixteen studies modelled the effect on consumption of<br>SSB taxes that ranged from 5% to 30%. All showed a reduction in consumption of these beverages, ranging from 5% to 48%, demonstrating overall a response in consumption that was proportional to the taxes applied. Of these, four studies that modelled substitution between beverages in response to taxes of 5–20% suggested that consumers would reduce consumption of SSBs, reducing caloric intake from these beverages by 10–48% in

	adults and by 5–8% in children. Six studies that did not consider substitution with other beverages also found significant reductions in consumption of sugar-sweetened beverages or soft drinks of 10–25% in response to taxes of 10–30%. Three studies of existing state-based soft drink taxes in the United States showed little difference in consumption between states with small taxes (around 5%) and states without such taxes. One study based on data from the USA Coronary Artery Risk Development in Young Adults found that a tax that increased the price of SSB by 10% could reduce consumption by 7%. Similarly, a study that used longitudinal data from the Nurses' Health Study to estimate the effect of modelled reductions in soft drink consumption found that a penny-per-ounce tax could reduce soft drink consumption by 15%.
Significance/direction	
Heterogeneity	
Meta-analytic model used	
RR/OR, 95% CI, P-value	
AMSTAR rating	Moderate
