# Supplementary material for: Effect of sugar-sweetened beverage taxation on sugars intake and dental caries: an umbrella review of a global perspective
Source: BMC Public Health. 2023 May 27;23:986. doi: 10.1186/s12889-023-15884-5 (PMC10224604; doi:10.1186/s12889-023-15884-5)
Supplement: Supplementary file 4 — Additional file 4. Data extraction forms for included systematic reviews addressing Question 3. [file 12889_2023_15884_MOESM4_ESM.pdf]

Additional file 4: Data extraction forms for included systematic reviews addressing Question 3.

**Study Details:**	
Author/year	**Moynihan, P.J**. and S.A. Kelly, Effect on caries of restricting sugars intake: systematic review to inform WHO guidelines. J Dent Res, 2014. 93(1): 8-18.
Objective	The objectives were to systematically review all available published data relating to the amount of sugars consumption and levels of dental caries and to report the findings for both adults and children.
Population (age/total number/gender proportions)	Children and adults
Setting/context (country)	Worldwide
Exposure	Sugars including total, free (or any component of i.e. added sugars, sucrose, non-milk extrinsic sugars) measured in either g, Kg, ounces per day, month, year.
Outcomes	Dental caries measured as DMFT/dmft, DMFS. dmfs, deft, det, indices, prevalence of dental caries, OR for caries, ECC Severe ECC
**Search details:**	
Databases searched	MEDLINE, EMBASE, Cochrane Database of Systematic Reviews, Cochrane Central Register of Controlled Trials, LILACS (Latin American and Caribbean Health Sciences), CNKI (China National Knowledge Infrastructure), Wanfang (China), and the South African Department of Health
Inclusion/exclusion criteria	Eligible for inclusion were intervention or observational studies, including randomised and non-randomised controlled studies, quasi-experimental studies, cohort, case-control, cross-sectional, and ecological studies Reviews were eligible if they contained a new analysis of data. Unpublished studies, or non-peer review articles, e.g., theses, abstracts and preprints were excluded.Studies were included if they reported any intervention intended to alter sugars intake in one study arm compared with diet with a different sugars content in another study arm, and also included information on dental caries, change in caries prevalence, incidence and or severity, or comparisons of higher vs lower caries as an outcome, with a timescale of at least one year. Observational studies were included if they reported absolute sugars or change in sugars

	intake and included information on dental caries. All timescales were included.
Range of years of included studies	1950-November 2011
Number of included studies	55
Design of studies included	Quasi-experimental, cohort, cross sectional, ecological
Country of origin of included studies	Argentina, Brazil, Denmark, Finland, Germany, Iceland, Iraq, Japan, Norway, Philippines, South Africa, Sweden, Switzerland, Turkey, UK, USA.
**Appraisal:**	
Quality assessment tool used/ratings	The Grading of Recommendations Assessment Development and Evaluation (GRADE) system (GRADE Working Group, 2004) was used to assess the quality of the body of evidence in relation to each of the review questions
How authors considered the quality	The GRADE quality was used to set the strength of the evidence.
**Analysis:**	
Method of analysis	Crude Meta-analysis was performed by pooling data across studies that reported the dental outcome in the same way (included mixed study design, ages, dental outcomes).<br>Vote count of studies reporting a positive, null or negative association between sugars exposure and dental outcome.<br>GRADE profile was conducted on best available evidence in terms of study design i.e. cohort.
Outcome assessed/type of measurement	Difference in dental caries by sugars intake.<br>Difference in sugars intake by dental caries.
Results/findings	42 out of 50 of those in children and 5 out of 5 in adults reported at least one positive association between sugars and caries. There is evidence of moderate quality showing that caries is lower when free-sugars intake is < 10% E. With the < 5% E cut-off, a significant relationship was observed, but the evidence was judged to be of very low quality. Meta-analysis of the data indicated a large effect size for the impact of sugars intake on dental caries [e.g., SMD for DMFT 0.82 (CI 0.67-0.97)]. Evidence for dose response and large size effect in individual studies e.g. Rugg-Gunn et al 1984 average increase of 1.28 DMFS over 2 yrs. with each rise of 100 g of sugars intake.<br>Burt et al 1991 Each additional 5 g sugars was associated with a 1% increase in the probability of developing caries.

	Takahashi et al 1959: Log-linear relationship between sugars and caries increment between 0.2 kg and 5-7.5 kg/ person/yr. in teeth erupted for 7-8 yrs., r = 0.8.
Significance/direction	Higher sugars associated with higher caries. Evidence of dose response
Heterogeneity	Considerable heterogeneity between studies.
Meta-analytic model used	Crude model based on standard mean differences.
RR/OR, 95% CI, P-value	Meta-analysis of the data indicated a large effect size for the impact of sugars intake on dental caries [e.g., SMD for DMFT 0.82 (CI 0.67-0.97)].
**Comments:**	
AMSTAR rating	High

**Study Details:**	
Author/year	**SACN**. Carbohydrates and Health. London: TSO, 2015
Objective	
Population (age/total number/gender proportions)	Children and adults
Setting/context (country)	Not stated
Intervention/exposure	Intake of carbohydrates (total CHO, starch, non-starch polysaccharide, resistant starch, sugars (amount and frequency and sources) of mono- and di-saccharides.
Outcome (dental caries measure)	Clinically assessed measure of caries e.g. DMFT/dmft, DMFS/dmft RCI. Measured visually at level of dentine or visual and radiographic assessment
**Search details:**	
Databases searched	Medline, Embase, CINAHL.
Inclusion/exclusion criteria	RCT and PCS, children and adults, with minimum information necessary to estimate HR, RR OR and with a measure of uncertainty e.g CI SD P-value.<br>Excluded non-peer reviewed studies, case controlled, cross sectional and ecological studies.
Range of years of included studies	Lit search conducted in January/February 2011. No other date limits stated.
Number of included studies	3 with data on quantitatively amount of sugars and development of dental caries
Design of studies included	Cohort observational studies
Country of origin of included studies	Finland UK, USA
**Appraisal:**	

Quality assessment tool used/ratings	Not conducted on the 3 PCS with data relevant to this review. Only done for RCT.
How authors considered the quality	RoB not considered in appraisal of results.
**Analysis:**	
Method of analysis	Narrative account of included studies. For all outcome measured the data were insufficient comparable to allow quantitative synthesis (pooling). Data were tabulated and described narratively.
Outcome assessed/type of measurement	Quantitative or qualitative clinical assessment of the progression or regression o caries in the primary or permanent dentition
Results/findings	Rugg-Gunn et al: Total sugar intake was significantly related to fissure caries increment after adjustment for confounders. A separate adjustment for tooth brushing (only 54% of the cohort provided information on this, n=219) did not attenuate the correlation. The overall caries increments in the cohort were low. For an increase in total sugars of 30g/day caries rose 0.36 DMFS (95%CI, -0.07-0.80).<br>Szpunar et al 1995: Higher sugar (% energy and g/day) significantly increased the probability of caries on all surfaces, but only higher sugar (% energy) significantly increased the probability of pit and fissure and approximal caries<br>Ruottinen et al: high sucrose intake associated with significantly increased caries experience.<br>A higher total sugar consumption was associated with increased risk of dental caries in three cohort studies.
Significance/direction	All studies showed positive association
Heterogeneity	Not measured
Meta-analytic model used	None used
RR/OR, 95% CI, P-value	
**Comments:**	Caution needed when interpreting correlation coefficients between discrete data (dental indices) and continuous data (sugars intake) which may underestimate the dental counts.
AMSTAR rating	Moderate
**Study Details:**	
Author/year	**Mahboobi, Z**., et al., Dietary free sugar and dental caries in children: a systematic review on longitudinal studies. Health Promot Perspect, 2021. 11(3): 271-280.
Objective	To assess the association between dietary free sugars consumption and dental caries in 6- to 12-year-old children in the recent longitudinal evidence. What is the dental outcome (considering incidence and/or progression) of high intake

	of DFS in comparison with low intake of DFS in schoolchildren?
Population<br>(age/total number/gender proportions)	Children aged 6-12 years
Setting/context (country)	Not stated
Intervention/exposure	Intake of sugary items
Outcome (dental caries measure)	“dental caries’ not further defined
**Search details:**	
Databases searched	PubMed, Embase, ISI Web of Science and Scopus
Inclusion/exclusion criteria	
Range of years of included studies	January 1, 2004 and September 22, 2019
Number of included studies	2 with data on quantitative amount of sugars and development of dental caries
Design of studies included	Cohort observational studies
Country of origin of included studies	Not listed
**Appraisal:**	
Quality assessment tool used/ratings	Newcastle Ottawa scale for observational studies.
How authors considered the quality	Listed score in table but did not consider score in evidence synthesis or interpretation of results
**Analysis:**	
Method of analysis	Narrative account of included studies – but provided no details of how this was applied to studies.
Outcome assessed/type of measurement	Prevalence ratio and IRR
Results/findings	Presented the findings narratively of Ruottinen et al, Karjalainen et al (both showing positive association between sucrose and dental caries) and Chankanta et al (no relationship between added sugar and dental caries) (7 quality score) Narratively.<br>Ruottinen: 4 quality score<br>Sucrose intake (SI) [high vs. low] and mean ± SD of caries as dmft (1.4 ± 2.0 vs. 0.5 ± 1.1, *P* = 0.01); Sucrose intake (SI) [high vs. low] and mean ± SD of caries as dmft+DMFT (3.9± 3.9 vs. 1.9 ± 2.5, *P* = 0.03)<br>Karjaleinen et al: 7 quality score<br>The mean ± SE of caries in high SI-group was higher than low SI-group (*P* = 0.046)<br>High sucrose intake **(**≥10% E**)** at age 3 was associated with higher risk of caries at age 6, 9, 12, 16.
Significance/direction	2 positive
Heterogeneity	Not measured
Meta-analytic model used	None used
RR/OR, 95% CI, P-value	
**Comments:**	

AMSTAR rating	Moderate
**Study Details:**	
**Author/year**	**Turck, D.**, et al., Tolerable upper intake level for dietary sugars. Efsa j, 2022. 20(2): e07074.
Objective	To provide scientific advice on a Tolerable Upper Intake Level (UL) or a safe level of intake for dietary (total/added/free) sugars on the basis of available data on chronic metabolic diseases and dental caries.
Population<br>(age/total number/gender proportions)	All age groups
Setting/context (country)	Europe and high-income countries
Exposure	Sugars (total, added, free, sucrose)
Outcomes	Caries incidence and prevalence measured as counts (e.g. DMFT/dmft indices) and % population affected.
**Search details:**	
Databases searched	Cochrane Library, Embase, PubMed and Scopus
Inclusion/exclusion criteria	
Range of years of included studies	2010 – 16/10/2020
Number of included studies	11 publications reporting on seven cohorts met the inclusion criteria. One cohort included adults of both sexes (Finnish cohort, (Bernabe et al., 2016)), one was in adult and older adult men(VA-DLS, (Kaye et al., 2015)), two were in adolescents of both sexes (UK cohort (Rugg-Gunn et al.,1984; Rugg-Gunn et al., 1987); Michigan cohort (Burt et al., 1988) (Burt and Szpunar, 1994; Szpunar et al., 1995)) and three were in children, again of both sexes (IFS (Chankanka et al., 2011); STRIP-1 (Ruottinen et al., 2004); STRIP-2 (Karjalainen et al., 2001, 2015).
Design of studies included	RCT, non-randomised trial, PCS
Country of origin of included studies	8 studies reported in 12 papers
**Appraisal:**	
Quality assessment tool used/ratings	OHAT (Finnish cohort and Michigan cohort low RoB, rest moderate except Va-DLS, which had high RoB).
How authors considered the quality	Reported for each individual study
**Analysis:**	
Method of analysis	Individual data from Strip Studies and IFS were obtained from authors and re-analysed. Data from other studies were considered as reported.
Outcome assessed/type of measurement	Caries, counts and prevalence (change in) measured using dental indices DMFT/dmft DMFS/dmfs RCI

Results/findings	Positive linear dose-response relationships have been observed between the intake of total sugars and risk of dental caries in permanent dentition and between the intake of sucrose and risk of dental caries in primary dentition in individual PCs across a wide range of total sugars and sucrose intakes. Dose-response relationships could not be explored across the BoE owing to the high heterogeneity of the exposures and endpoints assessed. Therefore, the data did not allow conclusions on the shape of the relationship between the intake of dietary sugars and risk of dental caries for any age group, or to identify a level of sugar intake at which the risk of dental caries is not increased.
Significance/direction	
Heterogeneity	Not formally measured
Meta-analytic model used	NA
RR/OR, 95% CI, P-value	NA
**Comments:**	
AMSTAR rating	Moderate
**Study Details:**	
**Author/year**	**Moores, C.J**., S.A.M. Kelly, and P.J. Moynihan, Systematic review of the effect on caries of sugars intake: ten-year update. J Dent Res, 2022: 220345221082918.
Objective	The aim was to systematically review epidemiological data published from 2011-2020 on the amount of sugars consumption and levels of caries and to report the findings for adults and children.
Population<br>(age/total number/gender proportions)	All ages
Setting/context (country)	Worldwide
Exposure	Sugars including total, free (or any component of i.e. added sugars, sucrose, non-milk extrinsic sugars) measured in either g, Kg, ounces per day, month, and year.
Outcomes	Dental caries measured as DMFT/dmft, DMFS. dmfs, deft, det, indices, prevalence of dental caries, OR for caries, ECC Severe ECC
**Search details:**	
Databases searched	MEDLINE, EMBASE, Cochrane Database, Cochrane Central Register of Controlled Trials, Latin American and Caribbean Health Sciences, China National Knowledge Infrastructure, Scopus and Google Scholar

Inclusion/exclusion criteria	Eligible for inclusion were intervention or observational studies, including randomised and non-randomised controlled studies, quasi-experimental studies, cohort, case-control, cross-sectional, and ecological studies published since November 2011. Reviews were eligible if they contained a new analysis of data not considered in the original review. Unpublished studies, or non-peer review articles, e.g., theses, abstracts and preprints were excluded.Studies were included if they reported any intervention intended to alter sugars intake in one study arm compared with diet with a different sugars content in another study arm, and also included information on dental caries, change in caries prevalence, incidence and or severity, or comparisons of higher vs lower caries as an outcome, with a timescale of at least one year. Observational studies were included if they reported absolute sugars or change in sugars intake and included information on dental caries. All timescales were included.
Range of years of included studies	2011-2020
Number of included studies	23
Design of studies included	Observational – Cohort, Case controlled, cross sectional and ecological
Country of origin of included studies	Australia, Brazil, Denmark Finland, Japan, Kenya, Malaysia, Netherlands, Poland, Puerto Rico, Thailand UK, USA
**Appraisal:**	
Quality assessment tool used/ratings	OHAT tool (full details of assessment provided in appendix)<br>GRADE profile of body of evidence pertaining to research questions.
How authors considered the quality	In the evidence synthesis harvest plots where more weighting was given to higher vs lower quality studies.
**Analysis:**	
Method of analysis	Vote counting and harvest plots were used for evidence synthesis
Outcome assessed/type of measurement	Weighting of evidence towards a positive vs null or negative association between sugars and dental caries. Supported by narrative data on evidence pertaining to dose response and size of effect.
Results/findings	11/15 studies in children and 6/8 studies in adults reported at least one positive association between sugars and caries. In children, 6/7 studies, and in adults 3/3 studies, with data

	enabling comparison of caries levels with sugars intakes >10%E and <10%E, showed lower caries when sugars intake was <10%E.
Significance/direction	Data were weighted towards a positive association between amount of sugars and risk of dental caries. Intakes of free sugars below 10% compared with >10% E were associated with lower caries. Intakes of free sugars <5% compared with>5% E were associated with lower dental caries. Included studies showed evidence of a dose response between sugars and dental caries as low as 2%E.<br>Included study of Finnish adult (Bernabe et al 2016) showed a linear dose response between sugars intake from 13.7 g/d (~2% EI)) to 442 g/d) and caries increment. For every 10-g/d sugars intake, DMFT increased by 0.09 (95% CI, 0.02–0.15), *P* = 0.14.
Heterogeneity	Not formally assessed
Meta-analytic model used	Vote counting and harvest plots
RR/OR, 95% CI, P-value	
**Comments:**	An update of the systematic review of Moynihan and Kelly 2014.
AMSTAR rating	High
