# Supplementary material for: Effect of sugar-sweetened beverage taxation on sugars intake and dental caries: an umbrella review of a global perspective
Source: BMC Public Health. 2023 May 27;23:986. doi: 10.1186/s12889-023-15884-5 (PMC10224604; doi:10.1186/s12889-023-15884-5)
Supplement: Supplementary file 5 — Additional file 5. AMSTAR results for included systematic reviews and evidence syntheses. [file 12889_2023_15884_MOESM5_ESM.pdf]

Additional file 5: AMSTAR results for included systematic reviews and evidence syntheses

**Review**	Q1	Q2	Q3	Q4	Q5	Q6	Q7	Q8	Q9	Q10	Q11	total	class
Itria et al 2021	Y	Y	Y	Y	N	Y	Y	Y	N	N	Y	8	High
Sobham et al 2019	N	Y	Y	Y	N	Y	Y	N	N	N	Y	6	Moderate
Teng 2019	Y	Y	Y	Y	N	Y	Y	Y	Y	Y	Y	10	High
Bergallo 2018	N	CA	Y	Y	N	Y	N	N	N	N	Y	4	Moderate
Redondo et al 2018	N	Y	Y	Y	N	Y	N	N	N	N	Y	5	Moderate
Afshin et al 2017	Y	Y	Y	Y	N	Y	Y	Y	Y	Y	Y	10	High
Nakhimovsky et al 2016	Y	N	Y	Y	N	Y	Y	Y	Y	N	Y	8	High
Backholer et al 2016	N	Y	Y	Y	N	Y	Y	N	N	N	Y	6	Moderate
Niebylski et al 2015	N	N	Y	Y	N	N	N	N	N	N	N	2	Low
Thow 2014	N	CA	Y	Y	N	Y	Y	N	Y	N	Y	6	Moderate
Cabrera Escobar, 2013	N	N	Y	N	N	Y	N	N	Y	Y	Y	5	Moderate
Maniadakis et al 2013	N	CA	Y	Y	N	Y	N	N	N	N	Y	4	Moderate
Powell et al 2013	N	Y	Y	Y	N	Y	N	N	Y	N	Y	6	Moderate
Andreyeva et al 2010	N	Y	Y	Y	Y	N	N	N	Y	N	N	5	Moderate
Moynihan & Kelly 2014	Y	Y	Y	Y	Y	Y	Y	Y	Y	N	Y	10	High
SACN 2015	N	N	Y	Y	Y	Y	N	N	NA	N	N	4	Moderate
Mahboobi et al 2021	Y	Y	Y	Y	N	Y	Y	N	N	N	Y	7	Moderate
Turck et al 2022	Y	CA	Y	Y	N	Y	N	Y	N	NA	Y	6	Moderate
Moores et al 2022	Y	Y	Y	Y	Y	Y	Y	Y	Y	N	Y	10	High

Y: yes, N: no, C/A: cannot answer, N/A: not applicable, *not done for studies relevant to this review.

Q1. **Was an 'a priori' design provided?**
Q2. **Was there duplicate study selection and data extraction?**
Q3. **Was a comprehensive literature search performed?**
Q4. **Was the status of publication (i.e. grey literature) used as an inclusion criterion?**
Q5. **Was a list of studies (included and excluded) provided?**

Q6. **Were the characteristics of the included studies provided?**
Q7. **Was the scientific quality of the included studies assessed and documented?**
Q8. **Was the scientific quality of the included studies used appropriately in formulating conclusions?**
Q9. **Were the methods used to combine the findings of studies appropriate?**
Q10. **Was the likelihood of publication bias assessed?**
Q11. **Was the conflict of interest included?**
