# Supplementary material for: Effect of sugar-sweetened beverage taxation on sugars intake and dental caries: an umbrella review of a global perspective
Source: BMC Public Health. 2023 May 27;23:986. doi: 10.1186/s12889-023-15884-5 (PMC10224604; doi:10.1186/s12889-023-15884-5)
Supplement: Supplementary file 6 — Additional file 6. Summary of original studies identified in included reviews addressing Question 1. [file 12889_2023_15884_MOESM6_ESM.docx]

**Additional file 6: Summary of original studies identified in included reviews addressing Question 1**

| **Author, year**  **Country** | **Objective/s** | **Study population and SSB consumption data** | **Relevant outcome variables** | **Consumption related results** | **Systematic Reviews using data** | **Quality assessment** |
| --- | --- | --- | --- | --- | --- | --- |
| Brown et al 1994  USA | To determine how price and income impact juice consumption | Neilson ScanTrack data on juice, soda, tea, and milk consumption households national data 1998-1992 | OPE | OPE of juices -0.70 to1.81.  OPE Soda -1.56. Found drinks are price elastic and substitution effect high. | Maniadakis et al (2013). | Quality of papers not assessed in review |
| Yen et al 2004  USA | To determine impact of price change on purchase on beverages | National Food Stamp Programme 908 households from nationally representative sample | OPE | OPE for soft drinks -0.80, juice -0.52. Compensated elasticity soft drinks -0.52; juice -0.35. Demand for soft drinks responsive to price. | Maniadakis et al (2013) | Quality of papers not assessed in review |
| Pofahl et al 2005  USA  (abstract) | Price elasticity of demand for non-alcoholic beverages modelling | Neilsen Home Scan data beverage consumption 1998-2001. 26255 households from representative sample. | Compensated elasticities of demand | Carbonated soft drinks -0.64; isotonics -1.93, fruit juice – 0.67. Prominent substitution evidence. Soft drinks and juices inelastic. | Maniadakis et al (2013) | Quality of papers not assessed in review |
| Barquera et al 2008  Mexico | Investigated PE of beverages including SSB in Mexico | Data from adolescents and adults  Nationally representative household surveys conducted in 1989, 1998 and 2006 (repeat cross-sectional) n=416 adolescents and 2180 adults from the Mexican Nutrition Survey 1999. | OPE | OPE was -0.61 in 1989; -0.85 in 1998 and -1.085 in 2006. Based on 2006 this equates to a 50ml decrease per household/day with a 10% price increase. Cross-price elasticity indicates increases in other beverages is smaller than decrease in soda. | Maniadakis et al (2013); Nakhimovsky et al (2016) | Meets 4/6 criteria assessed by Nakhimovsky et al (2016):  1) Is prospective-No  2) Data includes all SSB – yes  3) Price and consumption data from same population - yes 4) Considers potential substitution – yes  5) Effects of each SSB product analysed separately- yes  6) Study assesses an actual tax – no.  Quality of papers not assessed by Maniadakis et al (2013) |
| Brown 2008  USA | Determined price elasticity of demand juices milk and soda | Neilson data on weekly retail sales of consumers panel data 2003-2006. | OPE and cross-price elasticity | OPE juices -1.42; Soda -1.57. Negative price elasticity, demand in response to price varied little by income. | Maniadakis et al (2013)  Powell et al (2013). | Quality of papers not assessed in either review |
| Gustavsen 2008  Norway  (abstract) | Impact of tax on price and consumption of carbonated soft drinks | Norwegian household consumption surveys (1989-1999) 1200-1400 households and cross-sectional sample of 908 households from National Food Stamp Program Survey (1996-1997) | % Price change. Per capita consumption in L/year. | 10.8% price increase due to tax resulted in -5.1 (-9.5%) L/yr. consumption.  7.3% price increase due to tax resulted in -12.9L/yr. (-24%) | Maniadakis et al (2013) | Quality of papers not assessed in review |
| Zheng et al 2008a  USA | To determine price elasticity of non-alcoholic beverages and effect of advertising on demand | US Bureau of Labour and statistics. Data from Economic Research Service of Agriculture Dept. 1974-2005 | OPE | Price elasticity of soft drinks -0.52; juices -0.27. | Maniadakis et al (2013)  Powell et al (2013). | Quality of papers not assessed in review |
| Zheng et al 2008b  USA |  | Price/gallon from CPI report and food availability data ERS 1974-2005 US national sample | OPE | Carbonated soft drinks -OPE -0.609. | Powell et al (2013) | Quality of papers not assessed in review |
| Dharmasena et al 2009 (conference paper)  USA | Determined the OPE and cross-price elasticity of a number of specific sub-categories of drinks. | Neilson HomeScan data 1998-2003 | Price elasticity of demand | OPE: isotonic drinks-5.97; soft drinks -2.19; fruit drinks -0.13; fruit juice-0.18. Compensated elasticities: isotonic drinks-5.94; soft drinks -1.90; fruit drinks -0.08; fruit juice -0.82. | Maniadakis et al (2013) | Quality of papers not assessed in review |
| Dharmasena et al 2010 (abstract)  USA | to estimate OPE and cross-price elasticity of  selected non-alcoholic beverages; and to estimate the direct and indirect effects of 20%  excise tax on SSB consumption | Neilson HomeScan data 1998-2003, | % change in consumption of selected soft drinks | A 20% tax would reduce intake of regular SD by -14.33%. Fruit drinks by -13.43; Fruit juices by 12.75% and isotonics by -79.05%. Overall tax had a negative effect on consumption (reduced intake). | Maniadakis et al 2(0130 | Quality of papers not assessed in review |
| Finkelstein et al 2010  USA | To estimate the changes in energy, fat and sodium purchases and impact on body weight from a 20% ad valorem SSB tax and to explore  substitutions that may arise with other beverages | Predicted/simulated effect of 20% and 40% tax based existing data on reported consumption and expenditure from Neilsen HomeScan consumer data | Carbonated SSB and all SSB | 20%/40% tax would decrease:  1) carbonated SSB by a mean (SE) of 4.2 (1.6) kcal and 7.8 (2.8) kcal/capita/day (equivalent to ~1g and ~2g/day sugars) respectively.  2) All SSB would be reduced by a mean (SE) of 7 (1.9) kcal and 12 (3.4) kcal/capita/day (equivalent to ~1.8 and 3g/capita/day sugars) respectively.  Changes were largely driven by middle-income households.  No evidence of substitution to high sugars-containing foods. | Cabrera-Escobar (2013); Maniadakis et al (2013);  Powell et al (2013);  Thow et al (2014). | Meets 2/6 criteria assessed by Thow et al (2014):  1) Is prospective- no  2) Data includes all SSB – no  3) Price and consumption data from same population - yes 4) Considers potential substitution – no  5) Based on individual food consumption- yes  6) Study assesses an actual tax – no.  Meets 2/6 criteria assessed by Thow et al (2014):  1) Is prospective- no  2) Data includes all SSB – no  3) Price and consumption data from same population - yes 4) Considers potential substitution – no  5) Based on individual food consumption- yes  6) Study assesses an actual tax – no.  (2013); Maniadakis et al (2013);  Powell et al (2013); |
| Sturm et al 2010  USA | To examine whether small taxes are likely to  change consumption and weight gain or whether larger tax increases would be needed | **Data from children**  Early childhood longitudinal study Kindergarten cohort (individual SSB consumption) and State Level soda tax data.  5^th^ grade children aged 10-11 years old | SSB consumption frequency (serves/day) | State level taxes averaged 4.2% (ranging from 0 to 7%). Reduced serves of SSB by 0.142 (low SES children only) no effect for all populations.  No significant difference in consumption by tax level overall (but higher tax reduced consumption in lower income and African American ethic groups. | Maniadakis et al (2013);  Powell et al (2013); Thow et al (2014);  Backholer et al (2016); Teng et al (2019);  Itria et al (2021) | Quality of papers not assessed by Maniadakis et al (2013); Backholer et al (2016).  Meets 2/6 criteria assessed by Thow et al (2014):  1) Is prospective- no  2) Data includes all SSB – no  3) Price and consumption data from same population -no 4) Considers potential substitution – no  5) Based on individual food consumption- yes  6) Study assesses an actual tax -yes.  Quality assessed as low by Teng et al (2019) *:  Meets 4/7 criteria assessed by Itria et al (2021): 1) Is the study prospective evaluation of observed behaviour within the same population? - No  2) Do price and consumption data come from the same population? – Yes.  3). Does the study assess an actual tax or subsidy rather than hypothetical measures? –No.  4) Does the data include all SSB consumed? Yes.  5) Does the study report the effect of SSB intake on overweigh or obesity within the same population? - Yes  6) Does the study consider potential substitution to other products? - Yes  7). Does the study consider a long-run input data across time with sufficient variation in prices used to estimate price elasticities? (For experimental studies: a period of at least 1 month; for studies using existing data sets on SSB price: intervals no less than 2 months apart for at least 12 months. No.  Quality of papers not assessed by Maniadakis et al (2013);  Powell et al (2013);  Backholer et al (2016); |
| Fletcher et al 2010a; 2010b  USA | To explore impact of State soft drink tax rates (most <5%) on consumption of carbonated SSB | Data on children  NHANES III and IV (1998-1994/ 1996-2006) diet data 3-18 years. N=21040 (2010a) and 20968 (2010b). Price/tax data from Book of the States (1990-2007) States Dept. revenue websites | Energy intake (Kcal) and BMI | Mean sales tax was 2.7%. Level of tax led to modest reduction in consumption (some papers stated no reduction) but due to compensation, no impact on BMI.  20% tax led to a reduction in energy intake of 36kcal (equivalent to 4 grams sugars) but substitute may mean no overall impact on energy intake (but would be dental benefits) | Cabrera-Escobar (2013); Maniadakis et al (2013);  Powell et al 2(013); Thow et al 2014  Teng et al (2019);  Itria et al (2021) | Meets 2/6 criteria assessed by Thow et al 2014:  1) Is prospective- no  2) Data includes all SSB – no  3) Price and consumption data from same population - yes 4) Considers potential substitution – no  5) Based on individual food consumption- yes  6) Study assesses an actual tax – no.  Quality assessed by Teng et a; (2019) as High *  Addresses 4/7 criteria assessed by Itria et al (2021).  1) Is the study prospective evaluation of observed behaviour within the same population? No  2) Do price and consumption data come from the same population? No  3). Does the study assess an actual tax or subsidy rather than hypothetical measures? Yes.  4) Does the data include all SSB consumed? No  5) Does the study report the effect of SSB intake on overweigh or obesity within the same population? Yes.  6) Does the study consider potential substitution to other products? Yes.  7). Does the study consider a long-run input data across time with sufficient variation in prices used to estimate price elasticities? (For experimental studies: a period of at least 1 month; for studies using existing data sets on SSB price: intervals no less than 2 months apart for at least 12 months. Yes.  Quality of papers not assessed by Cabrera-Escobar 2013; Maniadakis et al 2013;  Powell et al 2013; |
| Andreyeva et al 2011  USA | Modelled (based on sales data) impact of tax on generated revenues and consumption and energy intake from SSB | Sales data from 2008 (state and city data) combined with prices elasticities from Andreyeva et al 2010 and Census population projections 2007-2015. | Consumption (% change) and impact on energy intake. | Chang in SSB consumption from a cent/oz.tax. SSB consumption reduced by 24%. Energy from SSB would reduce from 190-200 kcal to 145-150 kcal per person per day if there is no substitution i.e., 50 kcal change (50kcal is equivalent to 12.5g sugar/person/day). | Maniadakis et al (2013); Thow et al (2014). | Meets 3/6 criteria assessed by Thow et al (2014):  1) Is prospective- no  2) Data includes all SSB – no  3) Price and consumption data from same population - yes 4) Considers potential substitution – yes  5) Based on individual food consumption- yes  6) Study assesses an actual tax – no.  Quality of papers not assessed by Maniadakis et al (2013). |
| Brown 2011  USA | Determined price elasticity of demand juices milk and soda | Neilson data on weekly retail sales of consumers panel data 2007-2010. | OPE and cross-price elasticity | OPE Juice -1.56, soda -0.61. showed beverages have price elastic demands with relatedly large own and cross promotion affect. | Maniadakis et al (2013) | Quality of papers not assessed in review |
| Gustavsen & Rickertsen 2011  Norway | Modelled impact of increased VAT from 13%-25% on SSB (carbonated) | Household expenditure survey from 16000 households. 1989-2001 classified by low, medium and high consumption. | Volume of SSB purchased | Low consumers reduced by 5L. High consumers by 20L. Price increase of 10.6% reduced purchase by 10.8%. The % effect was highest in low-consumption households, but the absolute effect was highest among high-consuming households. | Thow et al (2014) | Meets 2/6 criteria assessed by Thow et al (2014):  1) Is prospective- no  2) Data includes all SSB – no  3) Price and consumption data from same population - yes 4) Considers potential substitution – no  5) Based on individual food consumption- yes  6) Study assesses an actual tax - no |
| Lin et al 2011  (Smith et al 2010 (grey literature)  USA | Determined price elasticity of demand and effects of 20% excise tax. Modelled the effect of a 20% tax on SSB on energy intake and body weight in children and adults | Children and Adults.  Price data from Nielson consumer panel (NCP) data 1998-2007 for price elasticities. NHANES data (7291 children and 8322 adults) (1998-2007; 2003-2006) and price data (NCP) 1998-2007. | Price elasticity and energy intake. | Price elasticity of SSB -0.95 to 0.05 in high income and -1.20 to -0.06 in low income.  Overall, 20% tax reduced energy intake by 36kcal/day (equivalent to 9g sugars). Energy intake from beverages decreased by 11% in both low- and high-income adults. And 12% in low- and 8% in high- income children. Took substitution into account.  A 20% tax would result in a decrease energy intake of 37kcal/adult/day and 43 kcal/child/day (equivalent to 9.25g and 10.7g added sugars respectively)  This varied by SES: Adults low income -38Kcal, High income – 36 Kcal,  Children low-income -36, high-income -50 all -44. | Itria et al (2021)  Thow et al (2014)  Cabrera-Escobar (2013)  Maniadakis et al (2013)  Powell et al (2013) | Meets 4/6 criteria assessed by Thow et al (2014):  1) Is prospective- no  2) Data includes all SSB – yes  3) Price and consumption data from same population - yes 4) Considers potential substitution – yes  5) Based on individual food consumption- yes  6) Study assesses an actual tax – no.  Meets 5/7 criteria assessed by Itria et al (2021):  1) Is the study prospective evaluation of observed behaviour within the same population? No  (2) Do price and consumption data come from the same population? Yes  3). Does the study assess an actual tax or subsidy rather than hypothetical measures? No  4) Does the data include all SSB consumed? Yes  5) Does the study report the effect of SSB intake on overweigh or obesity within the same population? Yes  6) Does the study consider potential substitute ion to other products? Yes  7). Does the study consider a long-run input data across time with sufficient variation in prices used to estimate price elasticities? (For experimental studies: a period of at least 1 month; for studies using existing data sets on SSB price: intervals no less than 2 months apart for at least 12 months. Yes.  Quality of papers not assessed by Cabrera-Escobar (2013)  Maniadakis et al (2013)  Powell et al 2(013) |
| Strum and Datar 2011  USA | Examined the association between regional prices and consumption frequency of fruit/vegetables and snack items among elementary school children in the USA. | Price per gallon regional food prices. Data from ECKS-K a nationally representative cohort of 1000 kindergarten 1000 schools in the USA followed up in 5^th^ grade (food diary) n=4896. | OPE | OPE for SSB 0.10 (0.17) (NS)  The absence of a price effect for sugar-sweetened beverages may indicate no consumer sensitivity to small price variations. | Powell et al (2013) | Quality of papers not assessed in review. |
| Bonnet & Requillart 2011  France | Determined the anticipated impact of an EU reform in sugar price of a 36% decrease on consumption of soft drinks and added sugars. | Used Kantar Wordpanel household purchase data 2003-5 from 19000 French households. And a 36% decrease in sugar price from European Union reforms | Soft drink consumption. Added sugars consumption | The 36% decrease in sugar price led to a 3% reduction in price of soft drinks and a 7.5% increase in consumption equivalent to 1L/capita/year or 124g added sugars/capita/year. | Cabrera-Escobar (2013); Thow et al (2021) | Meets 3/6 criteria assessed byThow et al (2014):  1) Is prospective-no  2) Data includes all SSB – no  3) Price and consumption data from same population - yes 4) Considers potential substitution – no  5) Based on individual food consumption- yes  6) study assesses an actual tax – yes.  Quality of papers not assessed by Cabrera-Escobar (2013); |
| Bonnet & Requillart 2012 (grey literature)  France | Modelled impact of 0.0716 euro per litre (~10%) price increase/tax on soft drinks | Kantar Wordpanel household purchase data 2003-5 from 19,000 French households | Consumption of carbonated soft drinks with SSB as subset | Soft drink consumption reduced by 3L/person/year (~15% reduction in consumption). SSB decreases more with SSB tax only. | Thow et al (2014) | Meets 3/6 criteria assessed by Thow et al 2(014):  1) Is prospective- no  2) Data includes all SSB – no  3) Price and consumption data from same population - yes 4) Considers potential substitution – no  5) Based on individual food consumption- yes  6) Study assesses an actual tax - yes |
| Claro et al 2012  Brazil | Quantify the effect of price of SSB on consumption | Individual (7-day food purchase record) cross-sectional data from 48,470 households, representative sample. Household Budget Survey 2002-2003. | Mean price of SSB per 1000Kcal and mean income per person per month. To estimate OPE for SSB. | OPE was -0.85 overall and -1.03 for lowest income group and -0.63 for other income groups.  Implies a 10% tax would reduce intake by 6.5% in non-poor and 10.3% in poor. | Cabrera-Escobar et al (2013); Maniadakis et al (2013);  Thow et al (2014);  Nakhimovsky et al (2016); | Meets 2/6 criteria assessed by Thow et al 2(014):  1) Is prospective- no  2) Data includes all SSB – no  3) Price and consumption data from same population - yes 4) Considers potential substitution – no  5) Based on individual food consumption- yes  6) Study assesses an actual tax – no.  Meets 1/6 criteria assessed by Nakhimovsky et al (2016):  1) Is prospective- no  2) Data includes all SSB – no  3) Price and consumption data from same population - yes 4) Considers potential substitution – no  5) Effects of each SSB product analysed separately- no  6) Study assesses an actual tax – no.  Quality of papers not assessed by Cabrera-Escobar et al (2013); Maniadakis et al (2013); |
| Dharmasena & Capps 2012 (Dharmasena et al 2011 abstract)  USA | Modelling study of impact of a 20% tax on energy intake from SSB (and other drink categories) | Nielsen HomeScan household consumer panel data 1998-2003 | Energy (kcal) from drink categories and other potential substitutes | 20% tax results in 49% decrease taking substitution to other products into account. Kcal from regular SSB decreased by 552/capita/month (equivalent to 4.5g/capita/day sugars). | Cabrera-Escobar (2013);  Powell et al (2013);  Thow et al (2014) | Meets 3/6 criteria assessed by Thow et al (2014):  1) Is prospective- no  2) Data includes all SSB – no  3) Price and consumption data from same population - yes 4) Considers potential substitution – yes  5) Based on individual food consumption- yes  6) Study assesses an actual tax – no.  Quality of papers not assessed by Cabrera-Escobar (2013;)  Powell et al (2013); |
| Lopez & Fantuzzi 2012  USA | Modelled impact of 10% tax on carbonated soft drinks on sales and energy intake. | Infoscan database on sales, volume sold and % volume with promotion. BRFSS (Behavioural Risk Factor Surveillance Survey) n=40000. | Sales and energy (kcal) | Regular carbonated soft drinks decreased 5.8%. Cross price within brand elasticities low compared with OPE. | Thow et al (2014) | Meets 2/6 criteria assessed by Thow et al (2014):  1) Is prospective- no  2) data includes all SSB – no  3) Price and consumption data from same population - yes 4) considers potential substitution – no  5) Based on individual food consumption- yes  6) Study assesses an actual tax – no. |
| Wang et al 2012  USA | Determine impact of $0.01/oz. tax on consumption of SSB | NHANES data 25-64 years (2003-2006) and published price elasticities from Andreyeva et al 2010 and average price. | Consumption (%). Energy intake | A cent (penny) per oz. would decrease SSB consumption by 15% (95% CI: 6, 24%) or 9 kcal/day (equivalent to 2.25g sugars). | Maniadakis et al (2013); Thow et al (2014); Niebylski et al (2015) | Meets 3/6 criteria assessed by Thow et al (2014):  1) Is prospective- no  2) Data includes all SSB – yes  3) Price and consumption data from same population – no  4) Considers potential substitution – yes  5) Based on individual food consumption- yes  6) Study assesses an actual tax – no.  Quality of papers not assessed by Maniadakis et al (2013) Niebylski et al (2015). |
| Briggs et al 2013  UK | To model the overall and income-specific effect of 20% sales SSB tax on prevalence of overweight and obesity in the UK | Adults aged 16+ years. National Diet and Nutrition Survey (2008-10)  Living Costs and Food Survey, 2010 | Energy intake. | 20% sales tax was modelled to lead to 15% decrease in purchase and 16.7kJ (95% CI: 11.3, 21.7) reduction in EI = 1g (95% CI: 0.67, 1.29) g sugar equivalent.  This reduction was 3.35 (95% CI 2.85, 3.83) g/d in 16-29 yrs.  Accounting for substitution, the changes in sugars were:  Low income: -4.4 (95% CI: -7, -1.8) g/d  Middle income -3.2 (95% CI: -5,1, - 1.1) g/d  High income -5.5 (-7,5 - -3.6) g/d. Differences NS.  There was higher substitution in lower and middle income SES. | Itria et al (2021) | Meets 4/7 criteria assessed by Itria et al (2021):  1) Is the study prospective evaluation of observed behaviour within the same population? No  (2) Do price and consumption data come from the same population? Yes  3). Does the study assess an actual tax or subsidy rather than hypothetical measures? No  4) Does the data include all SSB consumed? Yes  5) Does the study report the effect of SSB intake on overweigh or obesity within the same population? Yes  6) Does the study consider potential substitute ion to other products? Yes  7). Does the study consider a long-run input data across time with sufficient variation in prices used to estimate price elasticities? (For experimental studies: a period of at least 1 month; for studies using existing data sets on SSB price: intervals no less than 2 months apart for at least 12 months. No. |
| Briggs et al 2013b  Ireland | To model the impact of a 10% excise sugars tax on obesity. | Adults.  Price elasticity of demand for soft drinks in Ireland from 1980s data (−1.10.) and SLAN 2007 soft drink consumption data | Energy intake | Ireland Modelled 10% excise tax reduced energy intake by 2.1 kcal/person per day.  By SES:  Low – 2.2 (female) – 1.9 (male), average -1.9  Middle – 1.9 (female) -1.9( male) average – 1.9  High – 1.9 (female), -2.6 (male) average 2.3  Age Mean (range) reduction in daily energy intake (kcal/person/day) Female, Male, Overall  **18-24** 3.7 (2.9 to 4.5) 4.7 (3.7 to 5.8) 4.2 (3.3 to 5.2)  **25-34** 2.7 (2.1 to 3.3) 3.1 (2.4 to 3.8) 2.9 (2.3 to 3.5)  **35-44** 2.2 (1.7 to 2.7) 2.2 (1.7 to 2.7) 2.2 (1.7 to 2.7)  **45-54** 1.3 (1.0 to 1.6) 1.6 (1.3 to 2.0) 1.5 (1.1 to 1.8)  **55-64** 1.0 (0.8 to 1.2) 1.3 (1.0 to 1.6) 1.2 (0.9 to 1.4)  **65-74** 0.5 (0.4 to 0.7) 1.3 (1.0 to 1.6) 0.9 (0.7 to 1.1)  **75+** 0.7 (0.6 to 0.9) 0.9 (0.7 to 1.1) 0.8 (0.6 to 1.0)  **Overall** 1.9 (1.5 to 2.3) 2.3 (1.8 to 2.9) 2.1 (1.7 to 2.6)  Estimates are based on a tax pass on rate of 90%, price elasticity of −0.9 (range is for 80% and −0.8 respectively, and 100% and −1.0). Estimates for each age group were weighted for sex; and the overall estimate is weighted for age and sex of the Irish population. | Itria et al (2021) | Meets 4/7 criteria assessed by Itria et al (2021):  1) Is the study prospective evaluation of observed behaviour within the same population? No  (2) Do price and consumption data come from the same population? Yes  3). Does the study assess an actual tax or subsidy rather than hypothetical measures? No  4) Does the data include all SSB consumed? Yes  5) Does the study report the effect of SSB intake on overweigh or obesity within the same population? Yes  6) Does the study consider potential substitute ion to other products? Yes  7). Does the study consider a long-run input data across time with sufficient variation in prices used to estimate price elasticities? (For experimental studies: a period of at least 1 month; for studies using existing data sets on SSB price: intervals no less than 2 months apart for at least 12 months. No. |
| Finkelstein et al 2013  USA | Following on from 2010 paper (above) To estimate the impact of a 20% tax on store bought energy intake | Nielsen Homescan Consumer panel data 2006 on nationally representative sample | Energy intake from store purchased SSB (carbonated and all) | SSB tax decreased store-bought energy by 21.1kcal or 13.2 kcal/person/day according to model used) (this is equivalent to 5.3g/3.3g/sugar/day). Decreased SSB consumption was associated with only small substitution to other drinks and no substitution with other foods. | Cabrera-Escobar (2013);  Maniadakis et al (2013); | Quality of papers not assessed in either review. |
| Ni Mhurchu et al 2013  New Zealand | Estimated price elasticity (PE) values for major commonly consumed food groups in New Zealand, by income and ethnicity. | food expenditure data from national household economic surveys in 2007/08 and 2009/10 and Food Price Index data from 2007 and 2010 | OPE and cross-PE by quintile of household income. | Price elasticity (OPE) (SE) reported for Q1 (lowest income) to Q5 (highest income):  −2.20 (1.16); −3.47 (0 99); −0.14 (0.43); −2.95 (0.52); −1.27 (0 44) the difference between  quintiles 1 and 5 was not statistically significant | Backholer et al (2016) | Quality of papers not assessed in the review. |
| Basu et al 2014  India | to estimate the potential health effects of SSB tax in India | The Indian National Sample Surveyconsumption and price variations data from a nationally representative survey of 100,855 Indian households | OPE | Each 1% price increase led to 0.94% decrease consumption (OPE). | Itria et al (2021)  Nakhimovsky et al (2016) | Meets 4/6 criteria assessed by Nakhimovsky et al (2016):  1) Is prospective-No  2) Data includes all SSB – yes  3) Price and consumption data from same population - yes 4) Considers potential substitution – yes  5) Effects of each SSB product analysed separately- yes  6) Study assesses an actual tax – no.  Meets 5/7 criteria assessed by Itria et al (2021):  1) Is the study prospective evaluation of observed behaviour within the same population? No  (2) Do price and consumption data come from the same population? Yes.  3). Does the study assess an actual tax or subsidy rather than hypothetical measures? No.  4) Does the data include all SSB consumed? Yes.  5) Does the study report the effect of SSB intake on overweigh or obesity within the same population? Yes.  6) Does the study consider potential substitution to other products? Yes.  7). Does the study consider a long-run input data across time with sufficient variation in prices used to estimate price elasticities? (For experimental studies: a period of at least 1 month; for studies using existing data sets on SSB price: intervals no less than 2 months apart for at least 12 months. Yes. |
| Manyema et al 2014  South Africa | To estimate the effect of a  20% SSB tax on obesity among adults in South Africa. | Data from the 2012 SA National Health and Nutrition Examination Survey on consumption by persons aged 15+. Data from meta-analysis on OPE and cross-price elasticities of SSBs to estimate change in energy intake due to tax. | Energy intake | A 20% tax reduced energy intake by 36 (95% CI: 9.0, 68.0) kJ/day (equivalent to 2.1 (95% CI 0.5, 4.0) g sugars/day). | Itria et al (2021)  Nakhimovsky et al (2016) | Meets 2/6 criteria assessed by Nakhimovsky et al (2016):  1) Is prospective-No  2) Data includes all SSB – yes  3) Price and consumption data from same population – no 4) considers potential substitution – yes  5) Effects of each SSB product analysed separately- no  6) Etudy assesses an actual tax – no.  Meets 4/7 criteria assessed by Itria et al 2021:  1) Is the study prospective evaluation of observed behaviour within the same population? No  (2) Do price and consumption data come from the same population? Yes  3). Does the study assess an actual tax or subsidy rather than hypothetical measures? No  4) Does the data include all SSB consumed? Yes  5) Does the study report the effect of SSB intake on overweigh or obesity within the same population? Yes  6) Does the study consider potential substitute ion to other products? Yes  7). Does the study consider a long-run input data across time with sufficient variation in prices used to estimate price elasticities? (For experimental studies: a period of at least 1 month; for studies using existing data sets on SSB price: intervals no less than 2 months apart for at least 12 months. No. |
| Waterlander et a 2014  Holland | To examine the effects of 19% VAT on SSBs on beverage and snack purchases in an RCT within virtual web-based supermarket. | N=102 university staff/student randomly assigned to 1=) Test = 19% tax on SSBs (to reflect an increase in Dutch value added tax from 6% to 19%); or 2) control condition with regular prices. | Purchase of SSB on a single occasion at a three-dimensional Virtual Supermarket | 19% tax group purchased -0.9 (95% CI: -1.70, -0.10) L/household/week compared with control. There were no significant effects on purchases in other beverage or snack food categories. | Sobhani et al (2019) | Meets 5/7 criteria assessed:  1) Prospective study of observed behaviour; Yes.  2) Evaluation of an actual tax (rather than a hypothetical tax);Yes.  3) Price linked directly to purchase within same population; Yes.  4) Consideration of product compensation (cross-price elasticity); Yes.  5) Long‑run input data across time with sufficient variation in prices used to estimate price elasticities (for experimental studies this included data collected over a period of at least 1 month, for studies using existing data sets on SSB price this included data collected at intervals no <2 months apart for at least 12 months): Yes.  6) Valid and appropriate country‑specific data; No  7) Reporting of uncertainty around price elasticity estimates. No |
| Long et al 2015  USA | To quantify the anticipated health (BMI) and economic (DALYs/QALYs) benefits of $0.01/ounce SSB excise over 10 years. | Benefits estimated using a Markov cohort model to simulate the 2015 U.S. population aged 2+ years at baseline and followed them for 10 years until death or age 100 years. Baseline SSB data from 2011-2012 NHANES. Data on SSB Price and price elasticity from Powell et al 2014. | Price change and impact of consumption (%). | Baseline consumption of kcal from SSB 150 kcal/capita/day. Tax of $0.01/ounce in 2014 would increase prices by approximately 16%. Based on OPE of –1.21 (range, –0.69 to –3.87).  A 0.01c tax led to 20 (95% CI: 11, 43) % reduction in consumption (modelled). | Itria et al (2021) | Meets 5/7 criteria assessed by Itria et al (2021):  1) Is the study prospective evaluation of observed behaviour within the same population? No  (2) Do price and consumption data come from the same population? Yes  3). Does the study assess an actual tax or subsidy rather than hypothetical measures? No  4) Does the data include all SSB consumed? Yes  5) Does the study report the effect of SSB intake on overweigh or obesity within the same population? Yes  6) Does the study consider potential substitute ion to other products? Yes  7). Does the study consider a long-run input data across time with sufficient variation in prices used to estimate price elasticities? (For experimental studies: a period of at least 1 month; for studies using existing data sets on SSB price: intervals no less than 2 months apart for at least 12 months. Yes. |
| Batis et al 2016  Mexico | To examine  changes in purchase (volume) of taxed/ untaxed packaged food in response to tax, by SES | Data on packaged food purchases of 6248 households, representative of the Mexican urban population from The Nielsen Company’s Mexico Consumer Panel Services. Used a longitudinal, fixed-effects model that adjusted for pre-existing trends. Compared pre and post-tax trends. | Volume of SSB purchased as proxy for consumption | Low SES households 10.2% less taxed foods purchased (-44 (95% CI: –72, –16) g per capita per month); Medium SES households purchased 5.8% less taxed foods (-28 (range –46.0 to –11.0) g per capita per month). High SES households no change. Tax not fully transferred to prices | Bergallo (2018) | Quality of papers not assessed in the review. |
| Paraje et al 2016  Ecuador | To estimate OPE for SSB (Carbonated, energy and concentrated juices) to predict effect of tax | National Urban and Rural Household Income and Expenditure survey sample of 394334 households with sub analysis by income group.  Cross sectional data. | Expenditure per litre (price) and average quantity of SSB | OPE was -1.2 overall. -1.3 for lowest income group and -1.2 for other groups. | Nakhimovsky et al (2016) | Meets 3/6 criteria assessed by Nakhimovsky et al (2016):  1) Is prospective-no  2) Data includes all SSB – no  3) Price and consumption data from same population - yes 4) Considers potential substitution – no  5) Effects of each SSB product analysed separately- yes  6) Study assesses an actual tax - no |
| Veerman et al 2016  Australia | To estimate the impact of an additional 20% SSB tax on health and health care expenditure | Data from the Australian Health Survey (AHS) 2011–20. Australia-specific  price elasticities  based on  Sharma et al 2014 | Consumption as % and energy intake. | Modelling showed 20% tax reduced consumption by 12.6% and energy intake by 16 kJ/day in men and 9 kJ/day women (equivalent to 0.95g and 0.54g sugar respectively). | Itria et al (2021) | Meets 4/7 criteria assessed by Itria et al (2021):  1) Is the study prospective evaluation of observed behaviour within the same population? No  (2) Do price and consumption data come from the same population? Yes  3). Does the study assess an actual tax or subsidy rather than hypothetical measures? No  4) Does the data include all SSB consumed? Yes  5) Does the study report the effect of SSB intake on overweigh or obesity within the same population? Yes  6) Does the study consider potential substitute ion to other products? no  7). Does the study consider a long-run input data across time with sufficient variation in prices used to estimate price elasticities? (For experimental studies: a period of at least 1 month; for studies using existing data sets on SSB price: intervals no less than 2 months apart for at least 12 months. Yes. |
| Barrientos-Gutierrez et al 2017  Mexico | To estimate effect on body mass index, obesity and diabetes of a 1-peso/l  SSB tax. Also modelled a 2-peso/l tax scenario | The 2012 National Health and Nutrition  Survey: ‘ENSANUT-2012’ a nationally representative survey of 45,000 households (96,031 individuals). Percent reductions in SSB consumption attributable to the  tax were obtained from Colchero et al 2016b | Energy intake. | A 10% tax (1 peso/L) reduced SSB consumption by 21.6ml/person/day and 16.75 kcal (equivalent to 4.2g sugar). 20% tax (2 peso/L) reduced consumption by 43.2ml/person/d or 8.38 kcal (equivalent to 2.1g sugars). | Itria et al (2021) | Meets 4/7 criteria assessed by Itria et al (2021):  1) Is the study prospective evaluation of observed behaviour within the same population? No  (2) Do price and consumption data come from the same population? Yes  3). Does the study assess an actual tax or subsidy rather than hypothetical measures? No  4) Does the data include all SSB consumed? Yes  5) Does the study report the effect of SSB intake on overweigh or obesity within the same population? Yes  6) Does the study consider potential substitute ion to other products? No.  7). Does the study consider a long-run input data across time with sufficient variation in prices used to estimate price elasticities? (For experimental studies: a period of at least 1 month; for studies using existing data sets on SSB price: intervals no less than 2 months apart for at least 12 months. Yes. |
| Zhong et al 2018  USA | To determine immediate impact of $0.015/oz. tax on SSB and diet drinks on consumption implemented in Philadelphia. | A repeat cross-sectional study using data from a random-digit-dialling phone survey during a pre-tax period (December 2016) and a tax period (January -February 2017) among 899 respondents in Philadelphia and 878 in control cities. | Consumption (%) of soda, fruit drinks, energy drinks, and bottled water. | 0.015c tax led to 40% reduced consumption (modelled). | Itria et al (2021) | Meets 5/7 criteria assessed by Itria et al (2021):  1) Is the study prospective evaluation of observed behaviour within the same population? Yes  (2) Do price and consumption data come from the same population? Yes  3). Does the study assess an actual tax or subsidy rather than hypothetical measures? Yes  4) Does the data include all SSB consumed? No  5) Does the study report the effect of SSB intake on overweigh or obesity within the same population? Yes  6) Does the study consider potential substitute ion to other products? Yes  7). Does the study consider a long-run input data across time with sufficient variation in prices used to estimate price elasticities? (For experimental studies: a period of at least 1 month; for studies using existing data sets on SSB price: intervals no less than 2 months apart for at least 12 months. No. |
| **Naturalistic studies** | | |  |  |  |  |
| Bahl et al 2003  Ireland | To report on the impact of imposition and removal of excise tax on soft drinks in the Republic of Ireland | Natural experiment based on Republic of Ireland during periods of soft drink tax imposition and removal to investigate the revenue implications of reducing a discriminatory excise tax | Consumption (%) change. | SSB intake increased 6.8% following a decrease in tax | Niebylski et al (2015) | Niebylski et al (2015) cite the use of GRADE methodology to assess quality of studies but no information provided on individual studies. |
| Brown et al 2009  USA | Reduced the % of sugared drinks sold to <50% total drinks in vending machines (100% juice, water and lower sugar sports drinks were ‘healthful’ drinks increased the price of sugared by 10-25%. | Vending machines in US schools | Units of drinks sold. | Carbonated sugars soft drinks sales decreased by average of 45%. | Afshin et al (2017) | Quality of studies based on 5 criteria: study design, assessment of  exposure, assessment of outcome, control for confounding, and evidence of selection bias.  Score= 3/5 |
| Block et al 2010  USA (Boston) | To test whether a price increase on regular (sugary) soft drinks and an educational intervention would reduce their sales | Women’s hospital café 35% tax on regular soft drinks.  Then this tax plus educational campaign | Sales | 35% ($0.45 per drink) tax led to 26% sales reduction all soft drinks and 20% decrease in regular drinks.  Further 18% sales reduction with educational campaign. | Maniadakis et al (2013)  Afshin et al (2017) | Afshin et al 2017 assessed quality of studies based on 5 criteria: study design, assessment of  exposure, assessment of outcome, control for confounding, and evidence of selection bias.  Score= 5/5.  Quality of papers not assessed by Maniadakis et al (2013) |
| Duffey et al 2010 | Modelled effect of 10% price increase in SSB. | Cohort study n=5115 18–30-year-olds representative of population in 4 cities (FFQ) over 20 years diet data from 1985-1986; 1992, 1993; 2005-2006 compared with national food price data | Energy intake from soda (carbonated drinks). | 10% increase in price led to reduction of 7.12% (95% CI: -63.5, -10.7%) energy from soda. $1 increase in price of 2L bottle was associated with -124 (95% CI: -198, -50.0) kcal per day. (Equivalent to 31(95% CI: 49.5, 12.5) g sugar). | Maniadakis et al (2013); Thow et al (2014); Afshin et al (2017) | Quality of papers not assessed by Maniadakis et al (20-13).  Meets 1/6 criteria assessed by Thow et al (2014):  1) Is prospective- no  2) Data includes all SSB – yes  3) Price and consumption data from same population – no  4) Considers potential substitution – no  5) Based on individual food consumption- no  6) Study assesses an actual tax – no.  Afshin et al (2017) assessed quality of studies based on 5 criteria: study design, assessment of  exposure, assessment of outcome, control for confounding, and evidence of selection bias.  Score= 4/5. |
| Yang et al 2010  Taiwan | Impact of changes in soft drink price on purchase | Individual data from students aged 18-22 years | OPE and cross price elasticity | ‘Unhealthy’ beverage OPE -0.91. Cross price elasticity 0.69. | Maniadakis et al (2013) | Quality of papers not assessed in review |
| Gordon-Larsen et al 2011 | Determine how community-level food price variation was associated with individual-level fast food intake across waves II (1996) and III (2001–2002) of the National Longitudinal Study of Adolescent Health | Longitudinal data from nationally representative sample of US schools grades 7-12. And nationally representative price data (Council for the Community and economic research)  (n=11,088) from 158 baseline and 363 follow-up US counties | Visits to fast food outlets to consume soda. | A 20% Soda tax resulted in a decrease in visits to fast food outlets to buy soda with strongest effect being a -0.24 visits/ week. | Maniadakis et al (2013); Afshin et al (2017) | Quality of papers not assessed by Maniadakis et al (2013).  Afshin et al (2017) assessed quality of studies based on 5 criteria: study design, assessment of  exposure, assessment of outcome, control for confounding, and evidence of selection bias.  Score= 4/5. |
| Zhen et al 2011 | Estimated price elasticity of demand of soft drinks. Modelled impacts of 0.5cent per oz. tax | Neilsen NCP data 1998-2007. Sub-groups of low and high income Home created. 6161 low and 27045 high-income households. | Consumption and OPE | OPE: Regular carbonated soft drinks -1.06 to -1.54; Sports and energy drinks -0.53 to -1.52; Juice drinks -1.44 to -2.65. 0.5-cent tax decreased SSB by 118-135 cans/year in low and 110-128 cans/year in high-income households. | Powell et al (2013); Thow et al 2(014). | Meets 3/6 criteria assessed by Thow et al (2014):  1) Is prospective- no  2) Data includes all SSB – no  3) Price and consumption data from same population – yes  4) Considers potential substitution – yes  5) Based on individual food consumption- yes  6) Study assesses an actual tax – no.  Quality of papers not assessed by Powell et al (2013). |
| Zhen et al 2014 | Estimated impact of 0.5C/0z tax on price elasticity of demand, consumption and EI and compared low and high income households | Nielsen NCP data. analysis used the utility-theoretic Exact  Affine Stone Index (EASI) demand system, | Consumption, OPE and EI | OPE in low- and high-income households was-1.03 and -1.04 respectively. Impact on consumption was -65.8 and -49.3 oz./capita/quarter respectively. Impact on energy intake was -13.2 kcal and -5.6 kcal/capita/day for low and high-income households respectively (this is equivalent to 3.3g and 1.4g sugar/capita/day respectively). | Backholer et al (2016) | Quality of papers was not assessed in review. |
| Fletcher et al 2015 | To estimate non-linear effects of taxes using the range of current rates. To use, data on large soda tax increase in two states during the early 1990s combined with new synthetic control methods useful for comparative case studies. | NHANES data on adults 18+ and soda tax rates (average 5% (1%-12%) | Energy intake. | A 1% pt. increase in soda tax rates resulted in an increase in energy intake of 27.8 kcal per day (NS). Mean energy from soda was 130 kcal (5% total E) – 200 kcal from other soft drinks. 59% consumed soda.  No evidence of non-linear or threshold effects. | Teng et al (2019) | Quality assessed as High* |
| Grogger 2015 | Before/after analysis of Mexico’s CPI data on taxed and untaxed drinks to estimate how prices responded to the 2014 1 peso/Litre tax (~9/10%) on non-dairy SSB | Mexico consumer price index and retail prices from cities in Mexico | Price. | A tax of 9% raised price of regular soda by 12% (compared with prices pre-tax in December 2013). Little evidence of substitution to other caloric drinks. | Nakhimovsky et al (2016) | Meets 4/6 criteria assessed by Nakhimovsky et al (2016):  1) Is prospective-No  2) Data includes all SSB –no  3) Price and consumption data from same population - yes 4) Considers potential substitution – yes  5) Effects of each SSB product analysed separately- yes  6) Study assesses an actual tax – yes. |
| Colantuoni and Rojas 2015  USA | Measured effect of 5.5% sales tax on soft drinks in Maine in 1991 and a 5% sales tax on soft drinks in Ohio in 2003 on consumption | Sales data collected by scanner devices in the two states – before after tax data. | % change in sales and consumption | Neither 5.5% tax in Maine of 5% tax in Ohio had an impact on consumption of drinks.  [Redondo report this paper shows an 8% production tax in Berkeley compared with San Francisco and Oaklands. 21% reduced sales in Berkeley and 4% increase in control areas. P=0.046 – does not match citation] | Teng et al (2019).  Sobhani et al (2019).  Redondo et al (2018) | Quality assessed as Medium by Teng et al 2019*  Meets 4/7 criteria assessed by Sobhani et al 2019:  ) Prospective study of observed behaviour; Yes.  2) Evaluation of an actual tax (rather than a hypothetical tax);Yes.  3) Price linked directly to purchase within same population; Yes.  4) Consideration of product compensation (cross-price elasticity); No.  5) Long‑run input data across time with sufficient variation in prices used to estimate price elasticities (for experimental studies this included data collected over a period of at least 1 month, for studies using existing data sets on SSB price this included data collected at intervals no <2 months apart for at least 12 months), No.  6) Valid and appropriate country‑specific data: yes.  7) Reporting of uncertainty around price elasticity estimates. No.  6/58 quality criteria assessed by Redondo et al (2018) were not met, 38 were met (rest N/A).. |
| Falbe et al 2016  USA | To evaluate the impact of 1cent/oz. excise tax on SSB implemented in Berkeley, California, in March 2015. | Interrupted time-series study of sales of SSBs pre- and post-tax A beverage frequency  questionnaire Berkeley—873 low-income adults: 328 before and 545  4 months after the tax  Comparison cities—1806  adults: 662 pre-tax and 1144 post-tax. | Intake of SSB Regular soft drinks  Sports drinks  Energy drinks  Fruit drinks  Coffee or tea sweetened with  sugar | Consumption of SSB decreased by 21% and increased in comparison with 4% increase (p=0.046) states. Water consumption increased 63% (compared with 19% in comparison cities P<0.01)). | Teng et al (2019).  Sobhani et al (2019).  Redondo et al (2018). | Meets 5/7 criteria assessed by Sobhani et al (2019):  1) Prospective study of observed behaviour; Yes.  2) Evaluation of an actual tax (rather than a hypothetical tax); Yes.  3) Price linked directly to purchase within same population; Yes.  4) Consideration of product compensation (cross-price elasticity); No.  5) Long‑run input data across time with sufficient variation in prices used to estimate price elasticities (for experimental studies this included data collected over a period of at least 1 month, for studies using existing data sets on SSB price this included data collected at intervals <2 months apart for at least 12 months), Yes.  6) Valid and appropriate country‑specific data: yes.  7) Reporting of uncertainty around price elasticity estimates. No.  Quality assessed as Low by Teng et al 2019* |
| Ritter- Burga 2016  Peru | To determine if price decrease in soda increases consumption (and obesity) | Nationally representative household survey n-19658. Repeat cross sectional data | Soda price (6 month average)  Soda litres/person/month | The 10% decrease in soda price led to 90ml/person/month increase for all households (also associated with increased obesity). | Nakhimovsky et al (2016) | Meets 2/6 criteria assessed by Nakhimovsky et al (2016):  1) Is prospective-No  2) Data includes all SSB –no  3) Price and consumption data from same population - yes 4) Considers potential substitution – yes  5) Effects of each SSB product analysed separately- No  6) Study assesses an actual tax –No. |
| Colchero et al 2016a  2016b  Mexico | To estimate changes in sales of (SSB) and plain water after a 1-peso/ excise tax was implemented in Mexico in January 2014. | sales data from the Monthly Surveys of the Manufacturing Industry from January  2007 to December 2015 | changes  in per capita sales adjusting for season and the global indicator  of economic activity | Per capita sales of SSB decreased 7.3% and water sales increased by 5.2% in 2014 2015 compared to the pre-tax period (2007 2013). | Teng et al (2019).  Sobhani et al (2019).  Bergallo (2018). | Meets 4/7 criteria assessed by Sobhani et al (2019):  1) Prospective study of observed behaviour; Yes.  2) Evaluation of an actual tax (rather than a hypothetical tax);Yes.  3) Price linked directly to purchase within same population; Yes.  4) Consideration of product compensation (cross-price elasticity); No.  5) Long‑run input data across time with sufficient variation in prices used to estimate price elasticities (for experimental studies this included data collected over a period of at least 1 month, for studies using existing data sets on SSB price this included data collected at intervals no <2 months apart for at least 12 months), No.  6) Valid and appropriate country‑specific data: No.  7) Reporting of uncertainty around price elasticity estimates. Yes  Teng et a; (2019) rated quality of Colchero et al (2016a) as High*  Quality was not assessed by Bergallo et al (2018). |
| Aguilar et al 2017  Mexico | To measure the effects of two federal taxes in Mexico aimed at reducing obesity by taxing sugary drinks (SDs) and high caloric foods (HCFs | Kantar world panel weekly scanner panel dataset of 9953 households, with more than 58,721 product barcodes | % change in consumption | 6.3% decrease in SSB consumption with SE of 0.0006. | Teng et al (2019) | Quality assessed as Medium* |
| Colchero et al 2017a | Estimates changes in beverage purchases two years after (1 peso/litre) tax introduced | Household store purchase data for 6,645 households from January 2012 to December 2015. |  | Purchases of taxed (1 peso/l on non-dairy SSB) beverages decreased by 8.2% over the two years on average (-5.5% in 2014; -9.7% in 2015). With respect to reduced consumption in lowest SES -13.9ml/day (11.7%), Middle income 13.9ml (8.8%), highest income 7.6ml (5.1%).  Overall, relative decrease was 13.9ml/day (7.6%). | Itria et al (2021); Teng et al (2019)  Bergallo et al (2018) | Meets 5/7 criteria assessed by Itria et al (2021):  1) Is the study prospective evaluation of observed behaviour within the same population? No  (2) Do price and consumption data come from the same population? Yes  3). Does the study assess an actual tax or subsidy rather than hypothetical measures? Yes  4) Does the data include all SSB consumed? Yes  5) Does the study report the effect of SSB intake on overweigh or obesity within the same population? No  6) Does the study consider potential substitute ion to other products? Yes  7). Does the study consider a long-run input data across time with sufficient variation in prices used to estimate price elasticities? (For experimental studies: a period of at least 1 month; for studies using existing data sets on SSB price: intervals no less than 2 months apart for at least 12 months. Yes.  Teng et al (2019) rated as High*  Quality was not assessed by Bergallo et al (2018). |
| Colchero et al 2017b | Examined the heterogeneity of changes in SSB purchases after 1 peso/L tax introduced, by household income, urban vs. and rural location and household composition | Data from 4 rounds of the National Income and Expenditure Surveys: 2008, 2010, 2012, and 2014 | Changes in  purchases in litres/person/week and % change | Change in L/week (%)  Low income -0.09 (10.3)  Mid income -0.04 (3.7)  High income – 0.07 (5.8)  Urban – 0.08 (6.9)  Rural – 0.03 (3.9)  Adults only – 0.03 (2.4)  -plus children – 0.1 (11.0)  -plus adolescents – 0.11 (10.4)  all ages - -0.10 (11.6) | Teng et al (2019) | Teng et al (2019) rated as Medium* |
| Silver et al 2017  USA | examines the association of $0.01/oz. SSB excise tax Berkeley, California, with beverage prices, sales, store revenue/consumer spending, and usual beverage intake | Pre (before 1/1/2015) and first-year post taxation  (March 2015-end February 2016) measures of (1) beverage prices at 26 Berkeley stores; (2) point-of-sale scanner data on 15.5 million checkouts for beverage prices, sales,  for two supermarket chains covering three Berkeley and six control  supermarkets in adjacent cities; and (3) a representative telephone survey of 957 adult Berkeley residents | Change in sales. Change in energy intake from SSB | 0.67c/oz. led to decrease sales 9.6% (p<0.01) which rose in comparison stores by 6.9%.  Daily intake decreased by 19.8% equivalent to 6.4 kcal/person/day (4.2g sugar/person/day) NS. | Itria et al (2021).  Sobhani et al (2019).  Teng et al (2019) | Meets 5/7 criteria assessed by Itria et al (2021):  1) Is the study prospective evaluation of observed behaviour within the same population? Yes  (2) Do price and consumption data come from the same population? Yes  3). Does the study assess an actual tax or subsidy rather than hypothetical measures? Yes  4) Does the data include all SSB consumed? Yes  5) Does the study report the effect of SSB intake on overweigh or obesity within the same population? No  6) Does the study consider potential substitute ion to other products? Yes  7). Does the study consider a long-run input data across time with sufficient variation in prices used to estimate price elasticities? (For experimental studies: a period of at least 1 month; for studies using existing data sets on SSB price: intervals no less than 2 months apart for at least 12 months. No.  Meets 7/7 criteria assessed by Sobhani et al (2019):  Teng et al (2019) rated as High* |
| Alvarado et al 2017  Barbados |  |  | Price. | 10% tax led to 5.9% price increase. | Bergallo (2018) | Quality was not assessed by Bergallo et al (2018). |
| Caro et al 2018  Chile | Assessed impact on price and purchase of the Chilean government  increasing the tax rate from 13% to 18% on beverages with  > 6.25g/100ml sugars and decreasing the tax rate from 13% to 10% on beverages with <6.25 g/100ml sugar/100 mL). | Kantar WorldPanel Chile, longitudinal data collected between January 1, 2013, and December 31, 2015, from 2,000 households. | A pre-post analysis of change (%) in purchase and energy intake | 3.4% (95% CI -5.9, -0.9%) decrease per capita household purchase. 4% (95% CI -6.3, -1.9%) decrease in energy intake. | Teng et al (2019) | Quality rated as High* |
| Vall Castello and Cassanovas 2018 | Assessed impact of a tiered volumetric tax on SSB in Catalonia Spain | Before (2016) after (2017) comparison of sales data of drinks with 8g+/L sugar (taxed at 0.12 euro/L and drinks with 5-<8g/L sugars taxed at 0.08 euro/l, from a major supermarket chain (10% of market) | Volume of sales of SSB (including juice and milk based, excluding drinking yogurt and alcoholic) | Full pass to consumer (by law). Sales reduced by 7.7% SSB. OPE of drinks with 8+g/100ml sugar is -0.509 (10% tax would decrease consumption by 5.09%). 21% substitution to zero sugar brands.  22% of Catalan population consume drinks and decrease equates to a decrease of 42kcal/week (equivalent to 10.2g sugar/capita/week). | Teng et al (2019) | Quality rated as Medium* |
| Nakamura et al 2018  Chile | To evaluate Chile’s SSB tax implemented Oct 2014 | household-level grocery-purchasing data from 2011 to  2015 for 2,836 households living in cities representative of the urban population of Chile | Before/after comparison SSB purchase controlling for confounders | Little change in volume of all soft drinks purchased but a highly significant decrease in purchased volume of the higher-taxed (~1.6% price increase) sugary soft drinks by 21.6%. | Itria et al (2021);  Teng et al (2019) | Meets 5/7 criteria assessed by Itria et al (2021):  1) Is the study prospective evaluation of observed behaviour within the same population? No  (2) Do price and consumption data come from the same population? Yes  3). Does the study assess an actual tax or subsidy rather than hypothetical measures? Yes.  4) Does the data include all SSB consumed? Yes  5) Does the study report the effect of SSB intake on overweigh or obesity within the same population? Yes  6) Does the study consider potential substitute ion to other products? Yes  7). Does the study consider a long-run input data across time with sufficient variation in prices used to estimate price elasticities? (For experimental studies: a period of at least 1 month; for studies using existing data sets on SSB price: intervals no less than 2 months apart for at least 12 months. No.  Teng et al (2019) rated as High. |
| Alvarado et al 2019  Barbados | To assess whether and to what extent there has been a change in sales of SSBs (soda, sweetened juices and sports/energy drinks) following implementation of the 10% *ad valorem* SSB tax. | Price data provided by a major supermarket chain for sodas, sugar-sweetened juices, and sugar-sweetened sports and energy drinks (tax not applied to100% juices, sugar-free (diet) sodas, or sugar-free flavoured water) in Barbados over the period 2014-2016. | Sales of drinks. | 10% *ad valorem* tax led to 4.3 (95% CI: 3.6, 4.9) % decreased SSB sales (accounting for underlying trends). | Itria et al (2021). | Meets 4/7 criteria assessed by Itria et al (2021):  1) Is the study prospective evaluation of observed behaviour within the same population? No  (2) Do price and consumption data come from the same population? Yes  3). Does the study assess an actual tax or subsidy rather than hypothetical measures? Yes.  4) Does the data include all SSB consumed? No.  5) Does the study report the effect of SSB intake on overweigh or obesity within the same population? Yes  6) Does the study consider potential substitute ion to other products? No  7). Does the study consider a long-run input data across time with sufficient variation in prices used to estimate price elasticities? (For experimental studies: a period of at least 1 month; for studies using existing data sets on SSB price: intervals no less than 2 months apart for at least 12 months. Yes. |
| Capacci et al 2018  Capacci et al 2019 | estimate the price and consumption effects of the 2012 French tax on sweetened non-alcoholic drinks using a difference-in-difference approach | French CPI purchase prices and quantities from the 2011 and 2012 Kantar and GfK home-scan surveys 2011 and 2012 | Purchase | French tax €0.0716/L results in a small reduction in soft drink purchases (~500ml/capita/year). Analysis byTeng et al 2019 scaled these data to show a 10% tax would result in a 15.3% reduction in drinks consumed at home. | Teng et al (2019) | Teng et al (2019) rated as High. |

* Quality assessment by Teng et al based on 12 criteria: Study design, Inclusion of a control, Untaxed beverage outcomes reported, Representativeness of the taxed population, Same outcome in comparison groups,

Objectivity of the outcome, Correct classification of taxed and untaxed beverages, Same individuals or stores over time, Follow-up time points, Adjustment for major confounders, Accounts for changes in portion size, Reporting of any other health policies that were introduced with the SSB tax.

BMI, body mass index. CPI, consumer price index, kJ, kilojoules, NHANES, National Health and Nutrition Examination Survey. OPE, own price elasticity. SE, standard error, SES, socioeconomic status, SSB sugar sweetened beverage.

**References**

Afshin A, Penalvo JL, Del Gobbo L, Silva J, Michaelson M, O'Flaherty M, et al. The prospective impact of food pricing on improving dietary consumption: A systematic review and meta-analysis. Plos One. 2017; 12(3). e0172277. <https://doi.org/10.1371/journal.pone.0172277>

Aguilar A, Gutierrez E, Seira E. The effectiveness of sin food taxes: evidence from Mexico. The Latin American and Caribbean Economic Association (LACEA); 2017. Available from: http://vox.lacea.org/files/Working_Papers/lacea_wps_0010_aguilar_gutierrez_seira.pdfhttp://www.enriqueseira.com/uploads/3/1/5/9/31599787/obesidad_24jul17_esb.pdf. Accessed 15^th^ Feb 2022.

Alvarado M, Kostova D, Suhrcke M, Hambleton I, Hassell T, Samuels TA, et al. Trends in beverage prices following the introduction of a tax on sugar- sweetened beverages in Barbados. Prev Med. 2017;105S:S23–5.

Alvarado M, Unwin N, Sharp SJ et al. Assessing the impact of the Barbados sugar-sweetened beverage tax on beverage sales: an observational study. Int J Behav Nutr Phys Act 2019; 16, 13.

Andreyeva T, Chaloupka F J, Brownell K D. Estimating the potential of tax on sugar-sweetened beverages to reduce consumption and generate revenue. Prev Med 2011; 52(6): 413-416.

Backholer K, Sarink D, Beauchamp A, Keating C, Loh V, Ball K, et al. The impact of a tax on sugar-sweetened beverages according to socio-economic position: a systematic review of the evidence. Public Health Nutr. 2016;19(17):3070-84.

Bahl R, Bird R, Walker MB. The uneasy case against discriminatory excise taxation: Soft drink taxes in Ireland. Public Finance Rev 2003;31:510–33.

Barquera S, Hernandez-Barrera L, Tolentino ML, Espinosa J, Ng SW, Rivera JA, et al. Energy Intake from Beverages Is Increasing among Mexican Adolescents and Adults. Journal of Nutrition. 2008; 138

(12):2454 61. doi: 10.3945/jn.108.092163 PMID: 19022972

Barrientos-Gutierrez T, Zepeda-Tello R, Rodrigues ER et al. Expected population weight and diabetes impact of the 1-peso/l tax to sugar sweetened beverages in Mexico. PLoS One 2017; 12, e0176336.

Basu S, Vellakkal S, Agrawal S et al. Averting obesity and type 2 diabetes in India through sugar-sweetened beverage taxation: an economic-epidemiologic modelling study. PLoS Med 2014; 11, e1001582.

Batis C, Rivera JA, Popkin BM, Taillie LS. First-year evaluation of Mexico’s tax on nonessential energy dense foods: an observational study. PLoS Med. 2016 Jul 5; 13(7). https://doi.org/10.1371/journal.pmed.1002057 PMID: 27379797

Bergallo P, Castagnari V, Fernandez A, Mejia R. Regulatory initiatives to reduce sugar-sweetened beverages (SSBs) in Latin America. PLoS One. 2018;13(10):e0205694.Block JP, Chandra A, McManus KD, Willett WC. Point-of-purchase price and education intervention to reduce consumption of sugary soft drinks. Am J Public Health. 2010; 100(8):1427 33. doi: 10.2105/AJPH.2009.175687 PMID: 20558801

Bonnet C, Requillart V: Does the EU sugar policy reform increase added sugar consumption? An empirical evidence on the soft drink market. Health Econ 2011, 20(9):1012–1024.

Bonnet C, Requillart V. Sugar Policy Reform, Tax Policy and Price Transmission in the Soft Drink Industry. Working Paper No. 4, Transparency of Food Pricing (TRANSFOP) project. Brussels: European Commission; 2012.

Briggs ADM, Mytton OT, Kehlbacher A et al. Overall and income specific effect on prevalence of overweight and obesity of 20 % sugar sweetened drink tax in UK: econometric and comparative risk assessment modelling study. BMJ 2013a 347, f6189.

Briggs AD, Mytton OT, Madden D et al. The potential impact on obesity of a 10 % tax on sugar-sweetened beverages in Ireland, an effect assessment modelling study. BMC Public Health 2013b; 13, 860.

Brown GM. Impact of income on price and income responses in the differential demand system. Journal of Agricultural and Applied Economics. 2008; 40(2): 593-608.

Brown MG, Lee JY, Seale JL, Demand relationships among juice beverages: a differential demand system approach. Journal of Agricul­tural and Applied Economics. 1994; 26(2): 417-429.

Brown DM, Tammineni SK. Managing sales of beverages in schools to preserve profits and improve children's nutrition intake in 15 Mississippi schools. J Am Diet Assoc. 2009; 109(12):2036 42. doi: 10.1016/j.jada.2009.09.008 PMID: 19942021.

Brown MG, Jauregui CE. Conditional Demand System for Beverages. Gainesville, FL: Florida Department of Citrus; 2011. Available from: http://ageconsearch.umn.edu/bitstream/104335/2/RP%202011–1.pdf. Accessed Feb 2022 (grey literature).

Cabrera Escobar MA, Veerman JL, Tollman SM, Bertram MY, Hofman KJ. Evidence that a tax on sugar sweetened beverages reduces the obesity rate: a meta-analysis. BMC Public Health. 2013;13(1):1072. https://doi.org/10.1186/1471-2458-13-1072

Capacci S, Allais O, Bonnet C, Mazzocchi M. The impact of the French soda tax on prices, purchases and tastes: an ex post evaluation. Toulouse: University of Bologna; June 7–8; 2018.

Capacci S, Allais O, Bonnet C, Mazzocchi M. The impact of the French soda tax on prices and purchases. An ex post evaluation. PLoS ONE 2019; 14(10): e0223196. https://doi.org/10.1371/journal.pone.0223196

Caro JC, Corvalan C, Reyes M, Silva A, Popkin B, Taillie LS. Chile's 2014 sugar‐sweetened beverage tax and changes in prices and purchases of sugar‐sweetened beverages: an observational study in an urban environment. PLoS Med. 2018; 15(7):e1002597. <https://doi.org/10.1371/journal.pmed.1002597>

Castello J V, Lopez‐Casasnovas G. Impact of SSB taxes on consumption. Barcelona: Universitat Pompeu Fabra; April 2018. Available at: <https://www.tse‐fr.eu/publications/impact‐ssb‐taxes‐consumption>. Accessed Feb 18^th^ 2022.

Claro RM, Levy RB, Popkin BM, Monteiro CA. Sugar-Sweetened Beverage Taxes in Brazil. American Journal of Public Health. 2012; 102(1):178 83. doi: 10.2105/ajph.2011.300313.

Colantuoni F, Rojas C. The impact of soda sales taxes on consumption: evidence from scanner data. Contemp Econ Policy 2015;33:714–34.

Colchero MA, Salgado JC, Unar-Munguia M, Hernandez-Avila M, Rivera-Dommarco JA. Price elasticity of the demand for sugar sweetened beverages and soft drinks in Mexico. Econ Hum Biol. 2015;19:129 37. Epub 2015/09/20. doi: 10.1016/j.ehb.2015.08.007 PMID: 26386463.

Colchero MA, Guerrero-Lopez CM, Molina M, Rivera JA. Beverages sales in Mexico before and after implementation of a sugar sweetened beverage tax. PLOS one. 2016a Sep 26; 11(9). https://doi.org/10.1371/journal.pone.0163463 PMID: 27668875

Colchero MA, Popkin BM, Rivera JA, Ng SW. Beverages purchases from stores in Mexico under the excise tax on sugar sweetened beverages: observational study. BMJ 2016b; 352: h6704.19.

Colchero MA, Rivera-Dommarco J, Popkin BM et al. In Mexico, evidence of sustained consumer response 2 years after implementing a sugar-sweetened beverage tax. Health Aff 2017a; 36, 564–571.

Colchero MA, Molina M, Guerrero‐L Pez CM. After Mexico implemented a tax, purchases of sugar‐sweetened beverages decreased and water increased: difference by place of residence, household composition,and income level. J Nutr. 2017b 147(8): 1552‐1557.

Dharmasena S, Capps O Jr. Demand interrelationships of at home non-alcoholic beverage consumption in the United States. Poster presented at: Agricultural and Applied Economics Association and American Council on Consumer Interests Joint Annual Meeting; July 28, 2009; Milwaukee, WI.

Dharmasena S, Capps O Jr. On taxing sugar-sweetened beverages to combat the obesity problem. Poster presented at: Agricultural and Applied Economics Association, Canadian Agricultural Economics Society, and Western Agricultural Economics Association Joint Annual Meeting; July 27, 2010; Denver, CO.

Dharmasena S, Davis GC, Capps O Jr. Partial versus general equilibrium calorie and revenue effects of a sugar-sweetened beverage tax. Paper presented at: Joint Annual Meeting of the Agricultural and Applied Economics Association and the North eastern Agricultural and Applied Economics Association; July 24–26, 2011; Pittsburgh, PA.

Dharmasena S, Capps O: Intended and unintended consequences of a proposed national tax on sugar-sweetened beverages to combat the U.S. obesity problem. Health Econ 2012, 21(6):669–694.

Duffey KJ, Gordon-Larsen P, Shikany JM, Guilkey D, Jacobs DR Jr., Popkin BM. Food price and diet and health outcomes: 20 years of the CARDIA Study. Arch Intern Med. 2010; 170(5):420 6. PubMed Central PMCID: PMC3154748. doi: 10.1001/archinternmed.2009.545 PMID: 20212177

Falbe J, Thompson HR, Becker CM, Rojas N, McCulloch CE, Madsen KA. Impact of the Berkeley excise tax on sugar-sweetened beverage consumption. Am J Public Health 2016; 106:1865–71.

Fletcher JM, Frisvold DE & Tefft N. The effects of soft drink taxes on child and adolescent consumption and weight outcomes. J Public Econo 2010a; 94, 967–974.

Fletcher JM, Frisvold D & Tefft N. Taxing soft drinks and restricting access to vending machines to curb child obesity. Health Aff 2010b; 29, 1059–1066.

Fletcher JM, Frisvold DE, Tefft N. Non-linear effects of soda taxes on consumption and weight outcomes. Health Econ. 2015 May;24(5):566-82. doi: 10.1002/hec.3045. Epub 2014 Mar 10. PMID: 24615758; PMCID: PMC6047515.Finkelstein EA, Zhen C, Nonnemaker J, Todd JE: Impact of targeted beverage taxes on higher-and lower-income households. Arch Intern Med 2010, 170(22):2028–2034.

Finkelstein EA, Zhen C, Bilger M, Nonnemaker J, Farooqui AM, Todd JE Implications of a sugar-sweetened beverage (SSB) tax when substitutions to non-beverage items are considered. J Health Econ 2013, 32:219–239.

Gordon-Larsen P, Guilkey DK, Popkin BM. An economic analysis of community-level fast food prices and individual-level fast food intake: a longitudinal study. Health Place. 2011; 17(6):1235 41. PubMed Central PMCID: PMC3190083. doi: 10.1016/j.healthplace.2011.07.011 PMID: 21852178.

Grogger J. Soda Taxes and the Prices of Sodas and Other Drinks: Evidence from Mexico. National Bureau of Economic Research, Inc, NBER Working Papers: 21197; 2015.

Gustavsen G W. Public policies and the demand for carbonated soft drinks: a censored quantile regression approach. Poster presented at 11^th^ Congress of the European Association of Agricultural Economists. 2005 August 23-27 Copenhagen Denmark.

Gustavsen GW, Rickertsen K. The effects of taxes on purchases of sugar sweetened carbonated soft drinks: a quantile regression approach. Appl Econ.2011;43:707–716.

Itria A, Borges SS, Rinaldi AEM, Nucci LB, Enes CC. Taxing sugar-sweetened beverages as a policy to reduce overweight and obesity in countries of different income classifications: a systematic review. Public Health Nutr. 2021;24(16):5550-60.

Lin BH, Smith TA, Lee JY et al. Measuring weight outcomes for obesity intervention strategies: the case of a sugar-sweetened beverage tax. Econ Hum Biol 2-11; 9,329–341.

Long MW, Gortmaker SL, Ward ZJ et al. Cost effectiveness of a sugar-sweetened beverage excise tax in the US. Am J Prev Med 2015; 49, 112–123.

Lopez RA, Fantuzzi KL. Demand for carbonated soft drinks: implications for obesity policy. Appl Econ. 2012; 44:2859–2865.

Maniadakis N, Kapaki V, Damianidi L, Kourlaba G. A systematic review of the effectiveness of taxes on nonalcoholic beverages and high-in-fat foods as a means to prevent obesity trends. Clinicoeconomic Outc. 2013;5:519-43.

Manyema M, Veerman LJ, Chola L et al. The potential impact of a 20 % tax on sugar-sweetened beverages on obesity in South African adults: a mathematical model. PLoS One 2014; 9, e105287.

Nakamura R, Mirelman AJ, Cuadrado C et al. Evaluating the 2014 sugar-sweetened beverage tax in Chile: an observational study in urban areas. PLoS Med 2018; 15, e1002596.

Nakhimovsky SS, Feigl AB, Avila C, O'Sullivan G, Macgregor-Skinner E, Spranca M. Taxes on Sugar-Sweetened Beverages to Reduce Overweight and Obesity in Middle-Income Countries: A Systematic Review. PloS One. 2016;11(9) e0163358. Ni Mhurchu C, Eyles H, Schilling C et al. Food prices and consumer demand: differences across income levels and ethnic groups. PLoS One 2-13; 8, e75934.

Niebylski ML, Redburn KA, Duhaney T, Campbell NR. Healthy food subsidies and unhealthy food taxation: A systematic review of the evidence. Nutrition. 2015;31(6):787-95.

Paraje G. The Effect of Price and Socio-Economic Level on the Consumption of Sugar-Sweetened Beverages (SSB): The Case of Ecuador. PLoS One. 2016; 11(3): e0152260. Epub 2016/03/31. doi: 10.1371/journal.pone.0152260 PMID: 27028608.

Pofahl GM, Capps O jr, Clauson A. Demand for non-alcoholic beverages evidence from the AC Neilsen Home Scan Panel. Poster presentation at: American Agricultural Economics Association Annual Meeting July 24-17 2005. Providence RI.

Powell LM, Chriqui JF, Khan T, Wada R, Chaloupka FJ. Assessing the potential effectiveness of food and beverage taxes and subsidies for improving public health: a systematic review of prices, demand and body weight outcomes. Obes Rev. 2013;14(2):110-28.

Powell LM, Isgor Z, Rimkus L, Chaloupka FJ. Sugar-Sweetened Beverage Prices: Estimates from a National Sample of Food Outlets. Chicago, IL: Bridging the Gap Program, Health Policy Center, Institute for Health Research and Policy, University of Illinois at Chicago, 2014. [www.bridgingthegapresearch.org/_asset/ww9rpz/btg_SSB_price_brief_FINAL_Jan_2014.pdf](http://www.bridgingthegapresearch.org/_asset/ww9rpz/btg_SSB_price_brief_FINAL_Jan_2014.pdf) [accessed 1/4/2022].

Redondo M, Hernandez-Aguado I, Lumbreras B. The impact of the tax on sweetened beverages: a systematic review. Am J Clin Nutr. 2018;108(3):548-63.

Sharma A, Hauck K, Hollingsworth B, Siciliani L. The effects of taxing sugar-sweetened beverages across different income groups. Health economics. 2014; 23(9):1159–84. doi: 10.1002/hec.3070 PMID: 24895084.

Silver LD, Ng SW, Ryan-Ibarra S et al. Changes in prices, sales, consumer spending, and beverage consumption1 year after a tax on sugar-sweetened beverages in Berkeley, California, US: a before-and-after study. PLoS 2017; Med 14, e1002283.

Smith TA, Lin B-H, Lee J-Y. Taxing Caloric Sweetened Beverages: Potential Effects on Beverage Consumption, Calorie Intake, and Obesity. Washington DC: United States Department of Agriculture Economic Research Service; 2010.

Sobhani S, Babashahi M. Taxation for reducing purchase and consumption of sugar-sweetened beverages: A systematic review. Int Arch Health Sci. 2019;6(2):65-72.

Sturm R, Powell LM, Chriqui JF et al. Soda taxes, soft drink consumption, and children’s body mass index. Health Aff 2010; 29, 1052–1058.

Sturm R, Datar A. Regional price differences and food consumption frequency among elementary Schoolchildren. Public Health. 2011; 125:136–141.

Teng A, Snowdon W, Tin STW, Genc M, Na'ati E, Puloka V, et al. Progress in the Pacific on sugar-sweetened beverage taxes: a systematic review of policy changes from 2000 to 2019. Aust NZ J Publ Heal. 2021;45(4):376-84

Thow AM, Downs S, Jan S. A systematic review of the effectiveness of food taxes and subsidies to improve diets: Understanding the recent evidence. Nutr Rev. 2014;72(9):551-65.

Veerman JL, Sacks G, Antonopoulos N et al. The impact of a tax on sugar-sweetened beverages on health and health care costs: a modelling study. PLoS One 2016;11, e0151460.

Wang YC, Coxson P, Shen Y, Goldman L, Bibbins-Domingo K. A penny-per ounce tax on sugar-sweetened beverages would cut health and cost burdens of diabetes. Health Aff 2012; 31:199–207.

Waterlander WE, Ni Mhurchu C, Steenhuis IH. Effects of a price increase on purchases of sugar sweetened beverages. Results from a randomized controlled trial. Appetite 2014; 78:32-9.

Yang CC, Chiou WB. Substitution of healthy for unhealthy beverages among college students. A health-concerns and behavioural economic perspective. Appetite 2010; 54(3): 512-516.

Yen ST, Lin BH, Smallwood D M, Andrews M. Demand for non-alcoholic beverages: the case of low-income households. Agribusiness 2004; 20 (3): 309-312.

Zhen C, Wohlgenant MK, Karns S, Kaufman P. Habit Formation and Demand for Sugar-Sweetened Beverages. American Journal of Agricultural Economics. 2011; 93:175–193.

Zhen C, Finkelstein EA, Nonnemaker J et al. Predicting the effects of sugar-sweetened beverage taxes on food and beverage demand in a large demand system. Am J Agric Econ 2014; 96, 1–25.

Zheng Y, Kaiser H M. Estimating asymmetric advertising response: an application to US non-alcoholic beverage demand. Journal of Agricultural and Applied Economics 2008a; 40(3): 837-849.

Zheng Y, Kaiser HM. Advertising and U.S. Non-alcoholic Beverage Demand. Agricultural and Resource Economics Review. 2008b; 37:147–159.

Zhong Y, Auchincloss AH, Lee BK et al. The short-term impacts of the Philadelphia beverage tax on beverage consumption. Am J Prev Med 2018; 55, 26–34.
