# Supplementary material for: Effect of sugar-sweetened beverage taxation on sugars intake and dental caries: an umbrella review of a global perspective
Source: BMC Public Health. 2023 May 27;23:986. doi: 10.1186/s12889-023-15884-5 (PMC10224604; doi:10.1186/s12889-023-15884-5)
Supplement: Supplementary file 7 — Additional file 7. Details of original studies with data on sugars intake. [file 12889_2023_15884_MOESM7_ESM.docx]

**Additional file 7: Details of original studies with data on sugars intake.**

**Additional file 7 Table A. Original studies with data allowing for estimation of the impact of taxation on intake of sugars (Question 2).**

| **Author (year)/**  **Country** | **Objectives** | **Study population and SSB consumption data** | **Data on change in intake of sugars** |
| --- | --- | --- | --- |
| Finkelstein et al (2010)  USA | To estimate the changes in energy, fat and sodium purchases and impact on body weight from a 20% ad valorem SSB tax and to explore  substitutions that may arise with other beverages | Predicted/simulated effect of tax based existing data on reported consumption from Neilsen HomeScan consumer data from 2006. Energy content for each product was merged at the universal product code level using data available from Gladson Interactive Services, Lisle, Illinois, or via Web searches in which matches were not available. Mean daily kilocalories purchased per household member was estimated. | A 20% tax would decrease energy intake from carbonated SSB by a mean (SE) of 4.2 (1.6) kcal/capita/day (equivalent to ~1.2 (0.4) g /day sugars).  20% tax would reduce energy from all SSB by a mean (SE) of 7(1.9) kcal/ capita/day (equivalent to ~1.8 (0.48) g/ capita/day sugars).  No evidence of substitution to high sugars-containing foods. |
| Andreyeva et al (2011)  USA | Modelled (based on sales data) impact of tax on generated revenues and consumption and energy intake from SSB | Sales data from 2008 (state and city data) combined with prices elasticities from Andreyeva et al (2010) and Census population projections 2007-2015. Used the energy value of 190 kcal/15.8oz to derive impact on consumption | With a $0.01/ounce tax, energy intake from SSB would reduce from 190-200 kcal to 145-150 kcal per person per day (24% reduction in consumption) if there is no substitution. This averages 50 kcal/person/day decrease (50 kcal is equivalent to 12.5g sugar/person/day). |
| Lin et al 2011  (Smith et al 2010 (grey literature)  USA | Determined price elasticity of demand and effects of 20% tax. Modelled the effect of a 20% tax on SSB on energy intake and body weight in children and adults | Price data from Nielson consumer panel (NCP) data 1998-2007 for price elasticities. NHANES data (7291 children and 8322 adults) (1998-2007; 2003-2006) and price data (NCP) 1998-2007. Energy intake derived from NHANES data. | A 20% tax would result in a decrease energy intake of:  Adults: High income -35kcal (8.75g sugar), Low income -38 kcal (9.5 g sugar), All -36 kcal/day (9g sugar).  Children: High-income -50 kcal (12.5g sugar) Low income -36 Kcal (9g sugar) All -44 kcal (11g sugar).  Took substitution into account. |
| Bonnet & Requillart 2011  France | Determined the anticipated impact of an EU reform in sugar price of a 36% decrease on consumption of soft drinks and added sugars. | Used Kantar WorldPanel household purchase data 2003-5 from 19000 French households. And a 36% decrease in sugar price from European Union reforms | The 36% decrease in sugar price led to a 3% reduction in price of soft drinks and a 7.5% increase in consumption equivalent to 1L/capita/year or 124g added sugars/capita/year (0.34g/sugars/person/day). |
| Dharmasena & Capps 2012; Dharmasena et al 2011 abstract)  USA | Modelling study of impact of a 20% tax on energy intake from SSB (and other drink categories) | Nielsen HomeScan household consumer panel data 1998-2003. Energy content of drinks derived from Smith et al (2010). | A 20% tax results in 49% decrease, taking substitution to other products into account. Energy intake from regular SSB decreased by 552 kcal/capita/month (equivalent to 4.5g/capita/day sugars). |
| Wang et al 2012  USA | Determine impact of $0.01/Oz tax on consumption of SSB | NHANES data 25-64 years (2003-2006) and published price elasticities from Andreyeva et al 2010 and average price. Assumed 40% of energy compensated for, and used a net reduction of 60 kcals for every 100 kcal of SSB not consumed. | A cent (penny) per ounce would decrease SSB consumption by 15% (95% CI 6, 24%) or 9 kcal/day – based on 40% of kcal being compensated (equivalent to 2.25g sugars with compensation or 3.75 kcal without compensation). |
| Briggs et al 2013  UK | To model the overall and income-specific effect of 20% SSB tax on prevalence of overweight and obesity in the UK | Adults aged 16+ National Diet and Nutrition Survey (2008-10)  Living Costs and Food Survey, 2010 | A 20% sales tax was modelled to lead to a reduction in energy equivalent to 1g (0.67, 1.29) g sugar. This reduction was 3.35 (95% CI: 2.85, 3.83) g/d in 16-29 years age group. |
| Briggs et al 2013b  Ireland | To model the impact of a 10% sugars tax on obesity. | Price elasticity of demand for soft drinks in Ireland from 1980s data (−1.10.) and SLAN (National Survey of Lifestyles, Attitudes and Nutrition) 2007 soft drink consumption data. | Overall reduction in energy intake (kcal/person./day) was:  Female 1.9 (1.5 to 2.3)  Male 2.3 (1.8 to 2.9);  All 2.1 (1.7 to 2.6).  Effects were greater in young. |
| Finkelstein et al 2013  USA | Following on from Finkelstein et al 2010 paper (above), to estimate the impact of a 20% tax on store bought EI | Nielsen Homescan Consumer panel data 2006 on nationally representative sample.  Energy data as for Finkelstein et al 2010. | A 20% SSB tax decreased store-bought energy by 21.1 kcal or 13.2 kcal/person/day according to model used). (This is equivalent to 5.3g/3.3g/sugar/day). Decreased SSB consumption was associated with only small substitution to other drinks and no substitution with other foods. |
| Manyema et al 2014  South Africa | To estimate the effect of a  20% SSB tax on obesity among adults in South Africa. | Data from the 2012 South African National Health and Nutrition Examination Survey on consumption by persons aged 15+. Data from  meta-analysis on OPE and cross-price elasticities of SSBs to estimate change in energy intake due to tax.  SSBs were assumed to have an energy density  of 1800 kJ/litre (average of Coca Cola SSBs), | AA 20% tax reduced energy intake by 36 (95% CI 9, 68) kJ/day (equivalent to 2.1 (0.5, 4.0) g sugars/day). |
| Veerman et al 2016  Australia  mod | To estimate the impact of an additional 20% SSB tax on health and health care expenditure | Modelling study with data from the Australian Health Survey (AHS) 2011–20. Australia-specific price elasticities. Energy content of SSB derived from Australian food tables (NUTTAB). | A 20% tax reduced consumption by 12.6% and energy intake by 16 kJ/day in men and 9 kJ/day women (equivalent to 0.95g and 0.54g sugar respectively). |
| Barrientos-Gutierrez et al 2017  Mexico | To estimate effect on body mass index, obesity and diabetes of a 1-peso/l  SSB tax. Also modelled a 2-peso/l tax scenario | The 2012 National Health and Nutrition  Survey: ‘ENSANUT-2012’ a nationally  representative survey of 45,000 households (96,031 individuals). Percent reductions in SSB consumption attributable to the  tax were obtained from Colchero et al 2016. | A 20% tax (1 peso/L) reduced consumption by 43.2ml/person/day and 16.75 (SD 27.3) Kcal (equivalent to 4.2g sugar).  10 % tax (2 peso/L) reduced consumption by 21.6/person/d or 8.38 (SD 13.7) kcal (equivalent to 2.1g sugars).  No conflicts of interest declared.  Source of funding not declared. |
| **Naturalistic studies** | | |  |
| Barquera et al 2008 | Investigated the price elasticity of SSB, and impact of SSB tax on consumption in adults and adolescents | Nationally representative data from household surveys collected in 1989 and 2006 form 416 adolescents and 2180 adults from Mexican Nutrition Survey 1999. Data from the Mexican Health and Nutrition Survey-06 were used to derive energy intake from SSB. | A 10% tax (price increase) would reduce SSB intake by 50ml/household/day overall which was equivalent to 23 kcal (equating to 5.8g sugar per day). In low SES the decrease was -53ml and in high SES -46ml. |
| Duffey et al 2010  USA | Modelled effect of 10% price increase in SSB. | Cohort study n=5115 18–30-year-olds representative of population in four cities (FFQ) over 20 years diet data from 1985-1986; 1992, 1993; 2005-2006 compared with national food price data. | A one US dollar increase in price of 2L bottle was associated with -124 (95% CI -198, -50) kcal per day (equivalent to 31(49.5, 12.5) g sugar/day). |
| Zhen et al 2014  USA | Estimated impact of 0.5C/ounce tax on price elasticity of demand, consumption and EI and compared low- and high-income households | Nielsen NCP data. analysis used the utility-theoretic Exact Affine Stone Index (EASI) demand system. Used Gladson nutrient data for ~30% of Homescan products. For unmatched calculated mean Kcal/oz. using similar products or used values for similar products in the U.S. Department of Agriculture (USDA) National Nutrient Database for Standard Reference (USDA 2009). | Impact of 0.5 cents/ounce tax, on consumption in low- and high-income households was -65.8 and -49.3 per ounce/capita/quarter respectively. Impact on EI was -13.2 kcal and -5.6 kcal/capita/day for low- and high-income households respectively (this is equivalent to 3.3g and 1.4g sugar/capita/day respectively). |
| Fletcher et al (2010a; 2010b)  USA | Naturalistic study to explore impact of State soft drink tax rates (most <5%) on consumption | NHANES III and IV (1998-1994/ 1996-2006) diet data 3-18 years. N=21040 (in Fletcher et al 2010a) and 20968 (in Fletcher et al 2010b). Price/tax data from Book of the States (1990-2007) Department Revenue websites. | Mean sales tax was 2.7% level of tax led to modest reduction in consumption.  A 20% tax led to a reduction in energy intake of 36 (SE 14.6) kcal/person/day (equivalent to 9 (3.7) g sugars/person/day) but substitute to milk may mean no overall impact on energy intake (but would be reduced free sugars and therefore dental benefits) |
| Fletcher et al 2015  USA | To estimate non-linear effects of taxes using the range of current rates. To use, data on large soda tax increase in two states during the early 1990s combined with new synthetic control methods useful for comparative case studies. | NHANES data on adults 18+ (including data on energy intake and energy intake from soft drinks) and soda tax rates (average 5% (1%-12%) | Mean energy intake from soda was 130 kcal (5% total EI) – 200 kcal from other soft drinks. 59% consumed soda. Each 1% increase in soda tax rates resulted in an *increase* in energy intake of 1.6 kcal from soda per day (NS) (~0.4g sugar).  Researchers declare no conflicts of interest. |
| Silver et al 2017  USA | Examined the association of $0.01/oz. SSB excise tax Berkeley, California, with beverage prices, sales, store revenue/consumer spending, and usual beverage intake | Pre (before 1/1/2015) and first-year post taxation (March 2015-end February 2016) measures of (1) beverage prices at 26 Berkeley stores; (2) point-of-sale scanner data on 15.5 million checkouts for beverage prices, sales, for two supermarket chains covering three Berkeley and six control supermarkets in adjacent cities; and (3) a representative telephone survey of 957 adult Berkeley residents. NHANES data on energy intake from SSB. | One cent per fluid ounce tax led to 0.67c/ounce price increase which led to decrease sales 9.6% (p<0.01) which rose in comparison stores by 6.9%.  Daily intake decreased by 19.8% equivalent to 6.4 kcal/person/day (4.2g sugar/person/day) NS. However, baseline intake in Berkeley (45 kcal/day vs 131 kcal/d nationally). |
| Caro et al 2018  Chile | Assessed impact on price and purchase of the Chilean government  increasing the tax rate from 13% to 18% on beverages with > 6.25g/100ml sugars and decreasing the tax rate from 13% to 10% on beverages with <6.25 g/100ml sugar. | Kantar Worldpanel Chile, longitudinal data collected between January 1, 2013, and December 31, 2015, from 2,000 households. Information on EI from SSB obtained from nutrition labels on products. | 3.4% (-5.9, -0.9%) decrease per capita household purchase. A 4% (-6.3, -1.9%) decrease in energy intake. For a 2000 kcal/day diet, this is equivalent to 2g sugars/day. |
| Castello and Cassanovas 2018 | Assessed impact of a tiered volumetric tax on SSB in Catalonia Spain | Before (2016) after (2017) comparison of sales data of drinks with 8g+/L sugar (taxed at 0.12 euro/L and drinks with 5-<8g/L sugars taxed at 0.08 euro/l, from a major supermarket chain (10% of market). Collected data on sugars/L and on portion size to derive energy intake from SSB. | Full pass to consumer (by law). 10% tax would decrease consumption by 5.09%. 21% substitution to zero sugar brands.  22% of Catalan population consume drinks and decrease equates to a decrease of 42 kcal/week (equivalent to 10.2g sugar/capita/week). |

FFQ, food frequency questionnaire. Kcal, kilocalorie. kJ, kilojoule. NHANES, National Health and Nutrition Examination Survey. SSB, sugar sweetened beverage, USDA, US Department of Agriculture,

**References**

Andreyeva T, Long M, Brownell K. The Impact of Food Prices on Consumption: A Systematic Review of Research on the Price Elasticity of Demand for Food. American Journal of Public Health 2010; 100 (2): 216-222.

Andreyeva T, Chaloupka FJ, Brownell KD. Estimating the potential of taxes on sugar-sweetened beverages to reduce consumption and generate revenue. Prev Med. 2011;52(6):413-6.

Barquera S, Hernandez-Barrera L, Tolentino ML, Espinosa J, Ng SW, Rivera JA, et al. Energy Intake from Beverages Is Increasing among Mexican Adolescents and Adults. J Nutr. 2008;138(12):2454-61

Barrientos-Gutierrez T, Zepeda-Tello R, Rodrigues ER, Colchero MA, Rojas-Martinez R, Lazcano-Ponce E, et al. Expected population weight and diabetes impact of the 1-peso-per-litre tax to sugar sweetened beverages in Mexico. PLoS One. 2017;12(5):e0176336.

Bonnet C, Requillart V: Does the EU sugar policy reform increase added sugar consumption? An empirical evidence on the soft drink market. Health Econ 2011, 20(9):1012–1024.

Briggs ADM, Mytton OT, Kehlbacher A, Tiffin R, Rayner M, Scarborough P. Overall and income specific effect on prevalence of overweight and obesity of 20% sugar sweetened drink tax in UK: econometric and comparative risk assessment modelling study. BMJ. 2013;347:f6189

55. Briggs ADM, Mytton OT, Madden D, O'Shea D, Rayner M, Scarborough P. The potential impact on obesity of a 10% tax on sugar-sweetened beverages in Ireland, an effect assessment modelling study. BMC Public Health. 2013b;13. 860 https://doi.org/10.1186/1471-2458-13-860

Caro JC, Corvalan C, Reyes M, Silva A, Popkin B, Taillie LS. Chile's 2014 sugar-sweetened beverage tax and changes in prices and purchases of sugar-sweetened beverages: An observational study in an urban environment. Plos Med. 2018;15(7). e1002597.

Castello JV, Lopez-Casasnovas G. Impact of SSB taxes on consumption. Barcelona: Universitat Pompeu Fabra; 2018.

Colchero MA, Guerrero-Lopez CM, Molina M, Rivera JA. Beverages Sales in Mexico before and after Implementation of a Sugar Sweetened Beverage Tax. PLoS One. 2016;11(9):e0163463.

Dharmasena S, Capps O. Intended and unintended consequences of a proposed national tax on sugar-sweetened beverages to combat the U.S. obesity problem. Health Econ. 2012;21(6):669-94.

Dharmasena S, Davies GC, Capps OJ, editors. Partial versus genearl equilibrium calorie and revenue effects of a sugar-sweetened beveraged tax. Joint Annula Meeting of the Agricultural and Applied Economics Association and the North-eastern Agricultural and Applied Economics Association; 2011 July 24-26 2011; Pittsburgh P A.

Duffey KJ, Gordon-Larsen P, Shikany JM, Guilkey D, Jacobs DR, Popkin BM. Food Price and Diet and Health Outcomes 20 Years of the CARDIA Study. Arch Intern Med. 2010;170(5):420-6.

Finkelstein EA, Zhen C, Nonnemaker J, Todd JE. HEALTH CARE REFORM Impact of Targeted Beverage Taxes on Higher- and Lower-Income Households. Arch Intern Med. 2010;170(22):2028-34.

Finkelstein EA, Zhen C, Bilger M, Nonnemaker J, Farooqui AM, Todd JE Implications of a sugar-sweetened beverage (SSB) tax when substitutions to non-beverage items are considered. J Health Econ 2013, 32:219–239.

Fletcher JM, Frisvold DE, Tefft N. The effects of soft drink taxes on child and adolescent consumption and weight outcomes. J Public Econ. 2010;94(11-12):967-74.

Fletcher JM, Frisvold D, Tefft N. Taxing soft drinks and restricting access to vending machines to curb child obesity. Health Aff (Millwood). 2010b;29(5):1059-66.

Fletcher JM, Frisvold DE, Tefft N. Non-linear effects of soda taxes on consumption and weight outcomes. Health Econ. 2015 May;24(5):566-82. doi: 10.1002/hec.3045. Epub 2014 Mar 10. PMID: 24615758; PMCID: PMC6047515.Lin et al 2011

Manyema M, Veerman LJ, Chola L, Tugendhaft A, Sartorius B, Labadarios D, et al. The Potential Impact of a 20% Tax on Sugar-Sweetened Beverages on Obesity in South African Adults: A Mathematical Model. Plos One. 2014;9(8).e105287.

Silver LD, Ng SW, Ryan-Ibarra S, Taillie LS, Induni M, Miles DR, et al. Changes in prices, sales, consumer spending, and beverage consumption one year after a tax on sugar-sweetened beverages in Berkeley, California, US: A before-and-after study. Plos Med. 2017;14(4) e1002283. doi: 10.1371/journal.pmed.1002283.

Smith TA, Lin BH, Lee JY. Taxing Calorie Sweetened Beverages: Poteintal Effects on Beverage Consumption, Calorie Intake, and Obesity. Department of Agriculture Economic Research Service; Washington DC: 2010.

Veerman JL, Sacks G, Antonopoulos N, Martin J. The Impact of a Tax on Sugar-Sweetened Beverages on Health and Health Care Costs: A Modelling Study. Plos One. 2016;11(4).

Wang YC, Coxson P, Shen YM, Goldman L, Bibbins-Domingo K. A Penny-Per-Ounce Tax On Sugar-Sweetened Beverages Would Cut Health And Cost Burdens Of Diabetes. Health Affair. 2012;31(1):199-207.

Zhen C, Finkelstein EA, Nonnemaker JM, Karns SA, Todd JE. Predicting the Effects of Sugar-Sweetened Beverage Taxes on Food and Beverage Demand in a Large Demand System. Am J Agr Econ. 2014;96(1):1-25.

**Additional file 7 Table B: Quality ratings of original studies with data relating to the impact of SSB tax on intake of sugars (Question 2).**

| **Study** | **Itria et al 2020** | **Teng et al 2019** | **Afshin et al 2017** | **Nakhimovsky et al 2016** | **Backholer et al 2016** | **Thow et al 2014** | **Average % score across studies** |
| --- | --- | --- | --- | --- | --- | --- | --- |
| Finkelstein et al 2010 | - | - | - | - | 4/7  (57%) | 2/6  (33%) | 45% |
| Andeyeva et al 2011 | - | - | - | - |  | 1/6  (17%) | 17% |
| Lin et al 2011 | 5/7  (71%) | - | - | - | 5/7  (71%) | 4/6  (67%) | 70% |
| Bonnet & Requillart | - | - | - | - | - | - | Quality not assessed |
| Dham and Capps Dharmasena & Capps | - | - | - | - | - | 3/6  (50%) | 50% |
| Wang et al 2012 | - | - | - | - | - | - | Quality not assessed |
| Briggs et al 2013a | 4/7 (57%) | - | - | - | 4/7  (57%) | - | 57% |
| Briggs et al 2013b | 4/7  (57%) | - | - | - | 2/7  (29%) | - | 43% |
| Finkelstein et al 2013 | - | - | - | - | - | - | Quality not assessed |
| Manyema et al 2014 | 4/7  (57%) | - | - | 2/7  (29%) | - | - | 43% |
| Veerman et al 2016 | 4/7  (57%) | - | - | - | - |  | 57% |
| Barrientos-Gutierrez et al 2017 | 4/7  (57%) | - | - | - | - | - | 57% |
| Duffey et al 2010 |  |  | 4/5  (80%) |  |  | 1/6  (17%) | 48% |
| Zhen et al 2014 | - | - | - | - | 5/7  (71%) | - | 71% |
| Fletcher et al 2010 | 4/7  (57%) |  | 3/5  (60%) | - | - | 2/6  (33%) | 50% |
| Fletcher et al 2014 | 4/7  (57%) | 7/10  (70%) | - | - | - | - | 64% |
